# Benefits of Hormonal Contraception Across the Lifespan: A Case-Based, Interactive Curriculum

**DOI:** 10.15766/mep_2374-8265.11512

**Published:** 2025-04-04

**Authors:** Aisvarya Panakam, Andrea Pelletier, Natasha R. Johnson, Celeste S. Royce, Trevin C. Lau, Deborah Bartz

**Affiliations:** 1 Fourth-Year Medical Student, Harvard Medical School; 2 Research Associate, Obstetrics, Gynecology, and Reproductive Biology, Brigham and Women's Hospital; 3 Clerkship Director, Obstetrics and Gynecology Principal Clerkship Experience, Harvard Medical School and Brigham and Women's Hospital; 4 Clerkship Director, Obstetrics and Gynecology Principal Clerkship Experience, Harvard Medical School and Beth Israel Deaconess Medical Center; 5 Clerkship Director, Obstetrics and Gynecology Principal Clerkship Experience, Harvard Medical School and Massachusetts General Hospital; 6 Associate Clerkship Director, Obstetrics and Gynecology Principal Clerkship Experience, Harvard Medical School and Brigham and Women's Hospital

**Keywords:** Hormones, Contraception, Counseling, OB/GYN, LGBTQIA, Trauma-Informed Care, Diversity, Equity, Inclusion, Gender Equity, LGBTQ+ Health, Preventive Medicine, Flipped Classroom

## Abstract

**Introduction:**

Exogenous hormones found in birth control methods have contraceptive and noncontraceptive benefits, yet few educational resources exist for medical students to learn about use of hormonal contraceptives in varied patient scenarios.

**Methods:**

We designed a curriculum for second-year medical students on the use of hormonal contraceptives in patients with diverse identities and clinical conditions. Students enrolled in a transition to clerkship course participated in an interactive 40-minute large-group didactic, followed by case-based collaborative learning small-group discussions. Cases included a reproductive-aged female patient with a new chronic medical condition, a patient identifying as queer with menstrual irregularities, and a perimenopausal patient with systemic symptoms. Students provided immediate postcourse quantitative feedback, followed by longitudinal qualitative feedback during six consecutive OB/GYN clerkship cycles, regarding the value of the curriculum as preparation for hormone counseling patient encounters. We performed thematic analysis of all qualitative responses.

**Results:**

Of 137 students enrolled, 50 (36%) completed end-of-course evaluation, with 70% evaluating the intervention as *excellent* or *good*. For thematic analysis, of the 103 eligible clerkship students, 59 (57%) completed the survey. We identified four themes: value of the curriculum as an introduction to hormone counseling; value in recognizing one's own biases in hormone counseling; emphasis on patient-centered counseling; and introduction to value-neutral and nondirective counseling.

**Discussion:**

Our case-based curriculum introduces hormonal contraception across diverse patient identities and medical indications, resulting in improved student comfort with patient-centered counseling on hormone options. This expansive contraception curriculum can be replicated for other medical trainees across multiple subspecialties.

## Educational Objectives

By the end of this activity, learners will be able to:

1. Identify the need for and the considerations involved in providing family planning as part of the medical care plan for pregnancy-capable individuals at time of a new diagnosis of a chronic medical condition.
2. Using a trauma-informed care approach, describe assumptions, language, and gynecology health care practices that may contribute to medical mistrust.
3. Describe practice improvements to mitigate medical mistrust for a diversity of patients across the gender identity spectrum.
4. Describe the approach to history taking for perimenopausal patients.
5. Describe the role of exogenous hormones in perimenopausal symptom management.

## Introduction

Exogenous hormones found in modern contraceptive methods are the most common class of medications used; approximately 65% of the 60 million US pregnancy-capable people in their fecund years (ages 15 to 44 years) use these medications.^1^ Given this high prevalence of use coupled with the physical and social implications of unintended pregnancy, especially following the US Supreme Court 2022 overturn of the *Roe v. Wade* decision that previously protected the federal right to abortion, contraceptive counseling is an important skill set for professionals in a wide array of medical specialties and disciplines.^2^ Reliable, efficacious contraception is especially important for patients within medical subspecialty practices who have comorbid conditions that could worsen with pregnancy or in whom the primary medical condition may affect the health of the pregnancy.^3^ Furthermore, medical professionals should be aware of the numerous noncontraceptive, even nongynecologic, benefits of hormones, including treatment of catamenial mood disorders and seizures, migraines, anemia, gender dysphoria, and acne.^4–6^

Despite the widespread use and benefits of exogenous hormones, medical practitioners are insufficiently prepared to utilize and prescribe these medications for different indications. Primary care physicians affiliated with an urban academic center reported that medical school and residency inadequately prepared them to address teratogenic medications and perform contraceptive counseling. Furthermore, these physicians’ objective knowledge of teratogenic medications and contraceptive failure rates was lower than their self-perceived ability.^7^ Similarly, in a survey of US residents in family medicine, internal medicine, and OB/GYN programs, only 7% felt equipped to clinically manage menopausal symptoms.^8^

Although there is a clearly demonstrated need for improved, nuanced teaching on the many uses of hormonal contraception, we are unaware of curricula focused on the broad relevance of hormone and contraceptive counseling across the reproductive lifespan, as often seen in medical subspecialty clinics. Prior studies have described curricular innovations dedicated to the use of gender-affirming hormones to alleviate gender-dysphoric events,^9,10^ but to our knowledge there are no published education curricula dedicated to hormone use in non-trans LGBQIA+ individuals. Curricula focused on menopause have been developed for residents and for clerkship students,^11,12^ but we can find no prior educational work dedicated specifically to the perimenopausal phase.

We therefore developed a curriculum for preclinical medical students that presents an expanded framework regarding the benefits of the hormones within many modern contraceptive methods including pregnancy prevention in patients with medical comorbidities as well as noncontraceptive uses, such as menstrual suppression and perimenopausal symptom management. Our institution's preclinical curriculum is standardized in a daily pedagogy of cooperative learning, called case-based collaborative learning (CBCL), grounded in the social interdependence theory that recognizes the value of collaborative group learning over independent, siloed knowledge acquisition.^13,14^ The CBCL small-group discussions are based on situated learning theory, which posits that interactive discussion and activities with peer learners drives knowledge acquisition, through shared reflections, probing questions, and shared critical thinking.^15^ Our hormone curriculum is situated at the end of the preclinical (second) year of medical school, in the transition from preclinical to clinical education, as we aimed to educate medical students on the broad range of indications for hormonal contraceptives with a focus on three common patient populations, immediately prior to students beginning clinical care. Given the breadth of CBCL cases presented, we intended to provide teaching relevant throughout all clinical rotations, beyond a typical gynecology-only approach.

## Methods

We developed a 2-hour preclinical medical school curriculum dedicated to the use and benefit of the hormones in many modern contraceptive methods for patient populations that commonly present in clinical practices but are often missed in introductory contraception teaching: those with a new diagnosis of a chronic medical condition, a sex- and gender-minority status, and in perimenopause. Students completed preparatory readings, attended an interactive 40-minute large-group didactic, and engaged in three 35-minute CBCL small-group discussions. We assessed immediate satisfaction with the curriculum through quantitative course surveys and longitudinal experience of applying the learning during their OB/GYN clerkship 3 to 12 months after they had participated in the curriculum.

### Learners

Our target learners were second-year preclinical Harvard Medical School (HMS) Pathways students enrolled in a mandatory, 5-week transition to clerkship course in September 2020.^16^ This course bridges foundational pathophysiology from the preclinical year to the clinical reasoning skills utilized in clerkships. Learners received prior reproductive health content on endogenous female hormone physiology, both during and independent of pregnancy, in the first-year endocrinology block in two didactic sessions. Learners previously participated in a 3-hour CBCL session on the medical management of abnormal uterine bleeding, which included a 30-minute lecture on the pharmacology of modern hormonal birth control methods.

### Case Development

We developed the objectives and resulting cases to build on prior hormonal contraceptive pharmacology learning students had received, and to contribute to the course goals of ensuring that students achieve the knowledge, skills, and behaviors needed to enter the clerkship year. One of us, a faculty member specializing in complex family planning (Deborah Bartz), developed the content based on her knowledge of challenging concepts for medical students, OB/GYN residents, and peer faculty colleagues outside of OB/GYN. The selected CBCL cases were designed to extend beyond gynecology to primary care (Appendix A), to patients with marginalized identities (Appendix B), and across the reproductive life course (Appendix C). We piloted the curriculum in the preceding year's transition course (September 2019) and solicited immediate feedback from learners and small-group faculty. The only change between the pilot year and the subsequent research year was to shorten the cases and questions to allow full completion of all cases in the allotted 2 hours.

### Preparatory Assignments

Prior to starting the curriculum, we asked students to review as homework the three cases that would be discussed in the CBCL sessions (Appendices A–C). We provided students with resources to review, including the Centers for Disease Control and Prevention 2024 Summary Chart of US Medical Eligibility Criteria for Contraceptive Use (with recommendations to look for updates every 2 years since this resource was modified in 2024 from its original 2016 version; Appendix D), Bedsider Birth Control Network infographic (Appendix E), and Birth Control Across the Gender Spectrum infographic from the Reproductive Health Access Project (Appendix F). These materials provided content relevant to the CBCL curriculum, and students were encouraged to save these materials as references for use in clinical patient care. Materials were available to the students 2 weeks ahead of the curriculum through their web-based learning management system, but, given other learning commitments, most students likely reviewed this content the night before the class.

### Interactive Didactic Portion

In a 40-minute interactive lecture to the entire class, one of us (Deborah Bartz, a complex family planning physician) introduced students to contraception decision-making and counseling (Appendix G). Content covered included (1) public health topics, such as usage patterns of contraceptive methods in the US,^17^ (2) the efficacy of different methods of contraception,^18^ (3) the importance of social networks on patient's preference for specific contraceptives,^19^ (4) risk and eligibility determination for birth control methods for patients with medical conditions,^20^ (5) contraception coercion and bias within the medical community, especially as it relates to long-acting reversible contraceptives (LARCs),^21^ and (6) contraceptive and hormone replacement therapy for individuals in perimenopause and early menopause.^22,23^ This lecture is intended to introduce foundational principles in contraceptive and exogenous hormone counseling to guide case-based discussions.

### Case-Based Collaborative Learning

After the didactic portion, students were dispersed into their preexisting smaller learning communities, each consisting of 35–40 students and each with one OB/GYN faculty facilitator. Within these four learning communities, learners discussed cases in small groups of six or fewer students. These smaller groups underpin the HMS Pathways curriculum, as they allow learners to actively engage with their peers while learning course material and to receive more focused time with faculty.^16,24^ Within this setting, learners worked through the three cases and their associated questions (Appendices A–C) over 2 hours, with approximately 35 minutes devoted to each case. One faculty per learning community used the prepared small-group slides (Appendix H) to help facilitate discussion in the class. The faculty also had a faculty guide (Appendix I) with complete responses to each of the discussion questions to ensure standardization of teaching between small groups.

### Learner Feedback Collection and Analysis

We presented the curriculum on a single day in September 2020 for the whole HMS Pathways class in the course, immediately prior to students starting the clerkship year. A one-time email request was sent to participating students in October 2020 to complete the end-of-course quantitative evaluation with one question that asked learners to “Rate the Benefits of Hormonal Contraception Session in terms of its contribution to your learning” evaluated on a 5-point Likert-type scale (1 = *poor*, 5 = *excellent*) Once the clerkship year started, students in six OB/GYN clerkship cohorts received a separate, printed qualitative survey consisting of two free-response questions administered midway through their clerkship rotation, within 3 to 12 months after they had participated in the curriculum, to determine the longitudinal impact of the curriculum (Appendix J). No reminders or further recruitment of data were solicited. The first question asked students if their approach to contraceptive and exogenous hormone patient counseling during their clinical clerkships was guided by the preclerkship curricular intervention. The second question asked what hormone counseling, if any, students felt ill-prepared to do and wished they had received better prior instruction on.

Two investigators (Aisvarya Panakam and Andrea Pelletier) independently reviewed students’ free-text responses and identified themes using content analysis. To identify thematic categories, we searched for patterns and illustrative quotations based on analytical approaches of qualitative data analysis, including counting, comparison and contrast, and partitioning. These two reviewers convened to discuss, finalize, and define each theme, and then independently coded responses using these predetermined themes. This project was reviewed and approved by the HMS Program in Medical Education Educational Scholarship Review Committee in January 2021.

## Results

Among 137 learners who participated in the curriculum in September 2020, 50 (36%) completed the end-of-course quantitative survey, 70% of whom reported the curriculum as *excellent* or *good* in contribution to learning.

Over the longitudinal curriculum assessment period from January to September 2021, there were six cohorts of OB/GYN clerkship students at each of the three HMS-affiliated teaching hospitals Beth Israel Deaconess Medical Center (*n* = 33), Brigham and Women's Hospital (*n* = 36), and Massachusetts General Hospital (*n* = 34), for a total of 103 learners eligible to provide longitudinal qualitative assessment of the curriculum. Fifty-nine learners (57%) completed the qualitative survey during their OB/GYN clerkship.

Qualitative analysis of the impact of the curriculum through free-response answers given during the OB/GYN clerkship identified four themes (Table). More than half of students (71%) indicated that the curriculum served as a useful introductory framework to set the stage for further learning during the clinical year. Several learners (10%) reported that the curriculum helped them recognize their own biases as it relates to pregnancy prevention, contraceptive access, and contraceptive efficacy. Because of the emphasis on patient-centered contraceptive and hormonal counseling, some learners (23%) expressed that the curriculum enabled them to better engage in shared decision-making with their patients. Lastly, others (7%) expressed that the curriculum emphasized the importance of providing value-neutral and nondirective contraceptive counseling to patients.

**Table.**
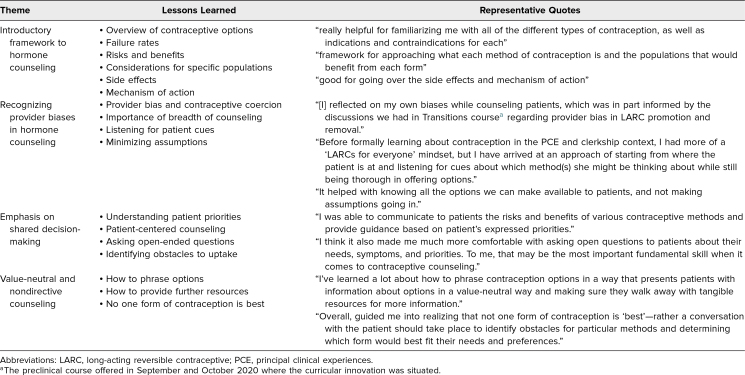
Learner Qualitative Responses Regarding the Influence of the Birth Control Hormone Curriculum on Contraceptive Counseling With Patients During the OB/GYN Clerkship

The second qualitative question asked learners what they felt ill-prepared to do during their clerkships and what hormone content they wished they had received better prior instruction on. For contraceptive-specific information, learners (59%) expressed unfamiliarity with the cost, side effect profile, contraindications, and generic versus brand names of hormonal contraceptives. Some learners (42%) indicated that the curriculum was too far removed from their current clerkship and that it was difficult to remember information from the session. Other learners (23%) expressed a desire for a hormone content refresher session or increased opportunities to practice counseling skills through role-playing or standardized patients. Learners (14%) also struggled to provide counseling regarding progestin-only contraceptives, fertility-based methods, and contraceptive options during the postpartum period. Some learners (9%) also reported that the amount of time spent on learning about hormones in the preclinical curriculum was minimal and that two sessions in the transition course were not sufficient to cover the subject.

## Discussion

To expand hormonal contraception education into patient populations and clinical scenarios often underrepresented in medical student learning, we developed a case-based curriculum for preclinical students that is dedicated to contraception for medically complicated patients and to noncontraceptive uses of birth control hormones. During end-of-course feedback, the majority of respondents found this curriculum to be *good* or *excellent* in contribution to their learning. Learners valued the curriculum as an introductory framework to hormone counseling, contraceptive coercion, shared decision-making, and nondirective counseling. In longitudinal assessment of the curriculum, we discovered that learners on the OB/GYN clerkship expressed lack of knowledge of other key factors in patients’ contraceptive and hormone decision making, including financial considerations, less commonly used methods, and method use postpartum. To address some of those systems-level modifiers of hormone and contraception provision, we have developed another postclerkship curriculum, required for all students, focused on reproductive justice.^25^

Our work builds upon the framework established by Ottolenghi and colleagues in 2019, in which a case-based flipped classroom model was used to teach preclinical medical students contraceptive pharmacology and risk communication.^26^ Our curriculum naturally segues from that work, as we focus on clinical applications rather than basic science, elicit participation of second-year medical students rather than first-year students; use an expanded framework for when hormones and contraception should be considered beyond pregnancy prevention in a healthy, straight, young person; and place further emphasis on noncontraceptive uses of hormonal contraceptives, such as menstrual suppression and perimenopausal symptom management. Prior curricula focusing on contraceptive counseling for medically complex female patients with focus on side effects, contraindications, and teratogenicity or on the clinical presentation of menopause exist for the resident level learners, but no equivalent body of work exists for medical students.^12,27^ Furthermore, Objective Structured Clinical Examinations focused on contraceptive counseling for LARCs and menopause management have been described in the literature; however, these curricula utilize standardized patients rather than the team-based learning approach centered in our curriculum.^11,28^

Strengths of this educational innovation include data collection from multiple cohorts of students throughout the clerkship year and across different sites, thereby increasing generalizability of our findings. Additionally, we piloted the didactic and CBCL courses for 1 year prior, which allowed fine-tuning of the curriculum to fit into our allotted time of 2 hours. This curriculum was well received at our institution and remains a part of the curriculum, now in its fifth year.

This educational innovation does have some limitations. First, because medical students are a vulnerable population within the hierarchy of medical training, our survey data are anonymous, to protect our learners. The feedback provided immediately after the intervention had a poor response rate of 36%, though this is consistent with the amount of course feedback we receive overall for our preclinical learning sessions. The longitudinal feedback response rate was much higher, at close to 60%. From a project perspective, because the survey data were anonymous, this negates our ability to comment on population-specific data of our responders versus nonresponders, and how these data might affect our results and conclusions. Furthermore, a notable weakness in the curriculum mentioned by respondents is the variable time interval between the curriculum and solicitation of qualitative feedback; for those students who did not have their OB/GYN clerkship in the first few blocks of the clerkship year, their ability to remember the details of the curriculum may have been compromised. This limitation emphasizes the importance of repeated, iterative teaching within the medical school curriculum. All three hospital clerkship sites do have a contraceptive didactic and a menopause didactic within the OB/GYN clerkship to once again review many of these hormone concepts. Furthermore, we developed a different curriculum dedicated to reproductive justice, as mentioned above, which is now required learning for all students in either their third or fourth year and where, once again, some of these hormone counseling concepts are reviewed.^25^ Therefore, although we only studied outcomes between preclinical learning and the clinical clerkship experience, it is possible that students’ overall knowledge of contraceptives and retention of this knowledge would be stronger when they graduate than at the learning time point we captured in this study, which takes into account their experience between only two learning touchpoints. Otherwise, developing other learning touchpoints, such as asynchronous learning modules or multidisciplinary contraception learning on other services, such as pediatrics or primary care, could both be employed to develop a more robust longitudinal curriculum. Lastly, while this curriculum was developed largely around the synthesis of hormonal contraceptive science and applying that to patient counseling, a portion of this curriculum may need adaptation from one patient population to the next or from one health policy environment to the next. For example, a religiously affiliated learning environment may need to adapt some of this content specific to their institution's restrictions.

It must be noted that this study did not evaluate the degree to which this curriculum met the learning objectives set out by the course directors. Future study could be employed to investigate this line of inquiry. Also, future work should investigate knowledge gaps in clinical counseling and optimal ways of introducing social and systems-level modifiers of patients’ hormone and contraception use. Additionally, we plan to study the efficacy of iterative, longitudinal hormonal contraception teaching as a whole, investigating the long-term impact of each of these curricula within a 4-year medical school family planning teaching program, to evaluate the impact of multiple touchpoints on contraceptive and hormone counseling. Only with concerted efforts in medical education will we achieve a comprehensive hormonal and contraceptive curriculum that familiarizes future generations of doctors to these ubiquitous and versatile medications used within a diverse array of patient identities and clinical indications.

## Appendices


Student Guide and Case 1.docxCase 2.docxCase 3.docxCDC Eligibility Criteria for Contraceptive Use.pdfBN How Well Does Birth Control Work.pdfRHAP Birth Control Across the Gender Spectrum.pdfCounseling for the Hormones Found in Contraceptives.pptxCase-Based Collaborative Learning.pptxFaculty Guide.docxLongitudinal Assessment Questions.docx
*All appendices are peer reviewed as integral parts of the Original Publication.*

## References

[1] Daniels K, Amba JC. Current Contraceptive Status Among Women Aged 15–49: United States, 2017–2019. Centers for Disease Control and Prevention; 2020. NCHS Data Brief No. 388. Accessed February 20, 2025. https://www.cdc.gov/nchs/data/databriefs/db388-H.pdf 33151146

[2] Eckhaus LM, Ti AJ, Curtis KM, Stewart-Lynch AL, Whiteman MK. Patient and pharmacist perspectives on pharmacist-prescribed contraception: a systematic review. Contraception. 2021;103(2):66–74. 10.1016/j.contraception.2020.10.012 33130109 PMC11283818

[3] Lathrop E, Jatlaoui T. Contraception for women with chronic medical conditions: an evidence-based approach. Clin Obstet Gynecol. 2014;57(4):674–681. 10.1097/GRF.0000000000000068 25264695

[4] Bahamondes L, Bahamondes V, Shulman LP. Non-contraceptive benefits of hormonal and intrauterine reversible contraceptive methods. Hum Reprod Update. 2015;21(5):640–651. 10.1093/humupd/dmv023 26037216

[5] Roden RC. Reversible intervention curriculums for menstrual management in adolescents and young adults with gender incongruence. Ther Adv Reprod Health. 2023;17:26334941231158251. 10.1177/26334941231158251 36938373 PMC10017940

[6] Williams NM, Randolph M, Rajabi-Estarabadi A, Keri J, Tosti A. Hormonal contraceptives and dermatology. Am J Clin Dermatol. 2021;22(1):69–80. 10.1007/s40257-020-00557-5 32894455

[7] Eisenberg DL, Stika C, Desai A, Baker D, Yost KJ. Providing contraception for women taking potentially teratogenic medications: a survey of internal medicine physicians’ knowledge, attitudes and barriers. J Gen Intern Med. 2010;25(4):291–297. 10.1007/s11606-009-1215-2 20087677 PMC2842551

[8] Kling JM, MacLaughlin KL, Schnatz PF, et al. Menopause management knowledge in postgraduate family medicine, internal medicine, and obstetrics and gynecology residents: a cross-sectional survey. Mayo Clin Proc. 2019;94(2):242–253. 10.1016/j.mayocp.2018.08.033 30711122

[9] Zheng C, D'Costa Z, Zachow RJ, Lebeau R, Bachmann GA. Teaching trans-centric curricular content using modified jigsaw. MedEdPORTAL. 2022;18:11257. 10.15766/mep_2374-8265.11257 35692604 PMC9127030

[10] Ellaway RH, Thompson NL, Temple-Oberle C, et al. An undergraduate medical curriculum framework for providing care to transgender and gender diverse patients: a modified Delphi study. Perspect Med Educ. 2022;11(1):36–44. 10.1007/S40037-021-00692-7 34792753 PMC8600495

[11] Reid HW, Branford K, Reynolds T, Baldwin M, Dotters-Katz S. It's getting hot in here: piloting a telemedicine OSCE addressing menopausal concerns for obstetrics and gynecology clerkship students. MedEdPORTAL. 2021;17:11146. 10.15766/mep_2374-8265.11146 33937522 PMC8079425

[12] Ng P, Kranz K, Abeles R, Schwartz D, Lane S. Using the jigsaw teaching method to enhance internal medicine residents’ knowledge and attitudes in managing geriatric women's health. MedEdPORTAL. 2020;16:11003. 10.15766/mep_2374-8265.11003 33117889 PMC7586752

[13] Johnson DW, Johnson RT. Cooperative, competitive, and individualistic learning. J Res Dev Educ. 1978;12(1):3–15.

[14] Johnson DW, Johnson RT. An educational psychology success story: social interdependence theory and cooperative learning. Educ Res. 2009;38(5):365–379. 10.3102/0013189X09339057

[15] Durning SJ, Artino AR. Situativity theory: a perspective on how participants and the environment can interact: AMEE Guide no. 52. Med Teach. 2011;33(3):188–199. 10.3109/0142159X.2011.550965 21345059

[16] Sullivan AM, Krupat E, Dienstag JL, et al. The Harvard Medical School Pathways curriculum: a comprehensive curricular evaluation. Med Teach. 2022;44(11):1268–1276. 10.1080/0142159X.2022.2081142 35764442

[17] Diep K, Long M, Salganicoff A. Oral contraceptive pills: access and availability. KFF. Updated March 20, 2024. Accessed February 20, 2025. https://www.kff.org/womens-health-policy/issue-brief/oral-contraceptive-pills-access-and-availability/

[18] Trussell J. Contraceptive failure in the United States. Contraception. 2011;83(5):397–404. 10.1016/j.contraception.2011.01.021 21477680 PMC3638209

[19] Yee L, Simon M. The role of the social network in contraceptive decision-making among young, African American and Latina women. J Adolesc Health. 2010;47(4):374–380. 10.1016/j.jadohealth.2010.03.014 20864007 PMC2945601

[20] Jensen JT, Creinin MD. Speroff & Darney's Clinical Guide to Contraception. 6th ed. Lippincott Williams & Wilkins; 2019.

[21] Dehlendorf C, Ruskin R, Grumbach K, et al. Recommendations for intrauterine contraception: a randomized trial of the effects of patients’ race/ethnicity and socioeconomic status. Am J Obstet Gynecol. 2010;203(4):319.e1–319.e8. 10.1016/j.ajog.2010.05.009 PMC3012124 20598282

[22] Freeman EW, Sammel MD, Sanders RJ. Risk of long term hot flashes after natural menopause: evidence from the Penn Ovarian Aging Cohort. Menopause. 2014;21(9):924–932. 10.1097/GME.0000000000000196 24473530 PMC4574289

[23] Rödström K, Bengtsson C, Lissner L, Milsom I, Sundh V, Björkelund C. A longitudinal study of the treatment of hot flushes: the population study of women in Gothenburg during a quarter of a century. Menopause. 2002;9(3):156–161. 10.1097/00042192-200205000-00003 11973438

[24] Krupat E, Richards JB, Sullivan AM, Fleenor TJ Jr., Schwartzstein RM. Assessing the effectiveness of case-based collaborative learning via randomized controlled trial. Acad Med. 2016;91(5):723–729. 10.1097/ACM.0000000000001004 26606719

[25] Ojo A, Singer MR, Morales B, et al. Reproductive justice: a case-based, interactive curriculum. MedEdPORTAL. 2022;18:11275. 10.15766/mep_2374-8265.11275 36310568 PMC9592687

[26] Ottolenghi J, Athauda G, Stumbar SE, Kashan SB, Lupi C. Contraceptive pharmacology and risk communication: a case-based flipped classroom exercise. MedEdPORTAL. 2019;15:10790. 10.15766/mep_2374-8265.10790 30800990 PMC6354791

[27] Worthington RO, Oyler J, Pincavage A, Baker NA, Saathoff M, Rusiecki J. A novel contraception counseling and shared decision-making curriculum for internal medicine residents. MedEdPORTAL. 2020;16:11046. 10.15766/mep_2374-8265.11046 33324751 PMC7727611

[28] Lupi CS, Mechaber AJ. Objective structured clinical examination: contraceptive counseling for long acting reversible methods. MedEdPORTAL. 2011;7:9021. 10.15766/mep_2374-8265.9021

