# Supplementary material for: Benefits of Hormonal Contraception Across the Lifespan: A Case-Based, Interactive Curriculum
Source: MedEdPORTAL. 2025 Apr 4;21:11512. doi: 10.15766/mep_2374-8265.11512 (PMC11968450; doi:10.15766/mep_2374-8265.11512)
Supplement: Supplementary file 1 — Student Guide and Case 1.docxCase 2.docxCase 3.docxCDC Eligibility Criteria for Contraceptive Use.pdfBN How Well Does Birth Control Work.pdfRHAP Birth Control Across the Gender Spectrum.pdfCounseling for the Hormones Found in Contraceptives.pptxCase-Based Collaborative Learning.pptxFaculty Guide.docxLongitudinal Assessment Questions.docx [file mep_2374-8265.11512-s001.zip › C. Case 3.docx]

**Small Group Case 3**

Appendix C: This document serves as the learner guide to the third case-based collaborative learning case dedicated to the benefits of the hormones in modern contraceptive methods in the setting of peri-menopause. Learners should have access to this case and accompanying questions in time to review in advance of the session. We provide this to our preclinical second year medical students two weeks in advance.

**You are an internal medicine provider about to see Cora, a 47-year-old cis-woman who has been your patient for several years. She is here for her annual visit with you. When talking to her, while she doesn’t endorse any specific complaints or reason for her visit besides her annual check-in, she doesn’t seem to be herself—she is less energetic, provides short answers, has trouble concentrating, and is less engaged overall. While you are thinking about medical conditions that may be contributing to her change in behavior, such as thyroid disorders, anemia, depression, you are also considering social contributors, or a new safety concerns in one of her relationships.**

**Upon doing a thorough review of symptoms and asking her about her social situation outside of your office and her feelings about being in the office with you today you discover that her sleep has been disrupted significantly both because of worry about her dissolving sexual relationship with her husband and because of feeling hot with some regularity. You also learn that her most recent menstrual cycle length was 60 days and that for the last 18 months she’s had her period every 3-6 months, lasting anywhere from 2-9 days. She seems to have more episodes of feeling hot when she has longer intervals between her cycles.**

**What is the differential diagnosis for her regular sensation of feeling hot, particularly at night?**

**What other aspects of her history may help you prioritize one particular diagnosis on the differential?**

**What further work up would you want to do to confirm your suspicion and rule out other diagnoses?**

**Once you make your diagnosis, how might you want to treat her underlying diagnosis?**

**What aspects of a patient’s past medical history may limit your therapies?**

**For Cora specifically, what therapies might we prioritize given her specific symptoms?**

**When a patient, such as Cora, as a multitude of symptoms that are interconnected (hot flashes contributing to sleep disturbances; sleep disturbances and sexual symptoms contributing to depressed mood) how do you consider and prioritize treatment in a patient-centered way?**
