# Supplementary material for: Benefits of Hormonal Contraception Across the Lifespan: A Case-Based, Interactive Curriculum
Source: MedEdPORTAL. 2025 Apr 4;21:11512. doi: 10.15766/mep_2374-8265.11512 (PMC11968450; doi:10.15766/mep_2374-8265.11512)
Supplement: Supplementary file 1 — Student Guide and Case 1.docxCase 2.docxCase 3.docxCDC Eligibility Criteria for Contraceptive Use.pdfBN How Well Does Birth Control Work.pdfRHAP Birth Control Across the Gender Spectrum.pdfCounseling for the Hormones Found in Contraceptives.pptxCase-Based Collaborative Learning.pptxFaculty Guide.docxLongitudinal Assessment Questions.docx [file mep_2374-8265.11512-s001.zip › D. CDC Eligibility Criteria for Contraceptive Use.pdf]

Appendix D. 2024 Centers for Disease Control Medical Eligibility Criteria (MEC) for contraception use. This resource was given to the learners ahead of the curriculum for their review. Content in both the interactive didactic and the case-based collaborative learning was dedicated to demonstrating how to use this resource. Learners were encouraged to use this resource throughout their clerkship year. Students were also instructed in the Student Guide (Appendix A) that this resource is updated occasionally and that they should look for an updated version every two years.

For accessible version, please see the summary of classifications at https://www.cdc.gov/contraception/hcp/usmec/.

Summary Chart of U.S. Medical Eligibility Criteria for Contraceptive Use (U.S. MEC)

Updated in 2024. This summary sheet only contains a subset of the recommendations from the U.S. MEC. For complete guidance, see: https://www.cdc.gov/contraception/hcp/usmec/. Most contraceptive methods do not protect against STIs. Consistent and correct use of the external (male) latex condom reduces the risk of STIs and HIV.
Please see NIH guidelines for up to date recommendations on hormonal contraception and ARVs: https://clinicalinfo.hiv.gov/en/guidelines/perinatal/prepregnancy-counseling-childbearing-age-overview?view=full#table-3 and https://clinicalinfo.hiv.gov/en/guidelines/hiv-clinical-guidelines-adult-and-adolescent-arv/drug-interactions-overview?view=full.

KEY: **1 = No restriction (method can be used)** | **2 = Advantages generally outweigh theoretical or proven risks** | **3 = Theoretical or proven risks usually outweigh the advantages** | **4 = Unacceptable health risk (method not to be used)**

Condition	Sub-Condition	Cu-IUD		LNG-IUD		Implant		DMPA		POP		CHC	
		I	C	I	C	I	C	I	C	I	C	I	C
**Age**		Menarche to <20 yrs:**2**		Menarche to <20 yrs:**2**		Menarche to <18 yrs:**1**		Menarche to <18 yrs:**1**		Menarche to <18 yrs:**1**		Menarche to <40 yrs:**1**	
		≥20 yrs:**1**		≥20 yrs:**1**		18–45 yrs:**1**		18–45 yrs:**1**		18–45 yrs:**1**		≥40 yrs:**2**	
						>45 yrs:**1**		>45 yrs:**2**		>45 yrs:**1**			
**Anatomical abnormalities**	a. Distorted uterine cavity	4		4									
	b. Other abnormalities	2		2									
**Anemia, iron-deficiency**		2		1		1		1		1		1	
**Benign ovarian tumors**	(*including cysts*)	1		1		1		1		1		1	
**Breast disease**	a. Undiagnosed mass	1		2*		2*		2*		2*		2*	
	b. Benign breast disease	1		1		1		1		1		1	
	c. Family history of cancer	1		1		1		1		1		1	
	d. Breast cancer‡												
	i. Current	1		4		4		4		4		4	
	ii. Past and no evidence of current disease for 5 years	1		3		3		3		3		3	
**Breastfeeding**	a. <21 days postpartum					2*		2*		2*		4*	
	b. 21 to <30 days postpartum												
	i. With other risk factors for VTE					2*		2*		2*		3*	
	ii. Without other risk factors for VTE					2*		2*		2*		3*	
	c. 30-42 days postpartum												
	i. With other risk factors for VTE					1*		2*		1*		3*	
	ii. Without other risk factors for VTE					1*		1*		1*		2*	
	d. >42 days postpartum					1*		1*		1*		2*	
**Cervical cancer**	Awaiting treatment	4	2	4	2	2		2		1		2	
**Cervical ectropion**		1		1		1		1		1		1	
**Cervical intraepithelial neoplasia**		1		2		2		2		1		2	
**Chronic kidney disease‡**	a. Current nephrotic syndrome	1	1	2	2	2		3		2/4*		4	
	b. Hemodialysis	1	1	2	2	2		3		2/4*		4	
	c. Peritoneal dialysis	2	1	2	2	2		3		2/4*		4	
**Cirrhosis**	a. Compensated (*normal liver function*)	1		1		1		1		1		1	
	b. Decompensated‡ (*impaired liver function*)	1		2		2		3		2		4	
**Cystic fibrosis‡**		1*		1*		1*		2*		1*		1*	
**Deep venous thrombosis (DVT)/Pulmonary embolism (PE)‡**	a. Current or history of DVT/PVE, receiving anticoagulant therapy (*therapeutic dose*)	2*		2*		2*		2*		2*		3*	
	b. History of DVT/PE, receiving anticoagulant therapy (*prophylactic dose*)												
	i. Higher risk for recurrent DVT/PE	2*		2*		2*		3*		2*		4*	
	ii. Lower risk for recurrent DVT/PE	2*		2*		2*		2*		2*		3*	
	c. History of DVT/PE, not receiving anticoagulant therapy												
	i. Higher risk for recurrent DVT/PE	1		2		2		3		2		4	
	ii. Lower risk for recurrent DVT/PE	1		2		2		2		2		3	
	d. Family history (*first-degree relatives*)	1		1		1		1		1		2	
**Depressive disorders**		1*		1*		1*		1*		1*		1*	

Condition	Sub-Condition	Cu-IUD		LNG-IUD		Implant		DMPA		POP		CHC	
		I	C	I	C	I	C	I	C	I	C	I	C
**Diabetes**	a. History of gestational disease	1		1		1		1		1		1	
	b. Nonvascular disease												
	i. Non-insulin dependent	1		2		2		2		2		2	
	ii. Insulin dependent‡	1		2		2		2		2		2	
	c. Nephropathy, retinopathy, or neuropathy‡	1		2		2		3		2		3/4*	
	d. Other vascular disease or diabetes of >20 years' duration‡	1		2		2		3		2		3/4*	
**Dysmenorrhea**	Severe	2		1		1		1		1		1	
**Endometrial cancer‡**		4	2	4	2	1		1		1		1	
**Endometrial hyperplasia**		1		1		1		1		1		1	
**Endometriosis**		2		1		1		1		1		1	
**Epilepsy‡**	(*see also Drug Interactions*)	1		1		1*		1*		1*		1*	
**Gallbladder disease**	a. Asymptomatic	1		2		2		2		2		2	
	b. Symptomatic												
	i. Current	1		2		2		2		2		3	
	ii. Treated by cholecystectomy	1		2		2		2		2		2	
	iii. Medically treated	1		2		2		2		2		3	
**Gestational trophoblastic disease (GTD)‡**	a. Suspected GTD (*immediate postevacuation*)												
	i. Uterine size first trimester	1*		1*		1*		1*		1*		1*	
	ii. Uterine size second trimester	2*		2*		1*		1*		1*		1*	
	b. Confirmed GTD												
	i. Undectectable or non-pregnant β-hCG levels	1*	1*	1*	1*	1*		1*		1*		1*	
	ii. Decreasing ß-hCG levels	2*	1*	2*	1*	1*		1*		1*		1*	
	iii. Persistently elevated ß-hCG levels or malignant disease, with no evidence or suspicion of intrauterine disease	2*	1*	2*	1*	1*		1*		1*		1*	
	iv. Persistently elevated ß-hCG levels or malignant disease, with evidence or suspicion of intrauterine disease	4*	2*	4*	2*	1*		1*		1*		1*	
**Headaches**	a. Nonmigraine (*mild or severe*)	1		1		1		1		1		1*	
	b. Migraine												
	i. Without aura (*includes menstrual migraine*)	1		1		1		1		1		2*	
	ii. With aura	1		1		1		1		1		4*	
**History of bariatric surgery‡**	a. Restrictive procedures	1		1		1		1		1		1	
	b. Malabsorptive procedures	1		1		1		1		3		COCs: 3<br>P/R: 1	
**History of cholestasis**	a. Pregnancy related	1		1		1		1		1		2	
	b. Past COC related	1		2		2		2		2		3	
**History of high blood pressure during pregnancy**		1		1		1		1		1		2	
**History of pelvic surgery**	(*see also Postpartum [including cesarean delivery]*)	1		1		1		1		1		1	
**HIV**	a. High risk for HIV	1*	1*	1*	1*	1		1		1		1	
	b. HIV infection					1*		1*		1*		1*	
	i. Clinically well receiving ARV therapy	1	1	1	1	*If on ARV, see also Drug Interactions.*							
	ii. Not clinically well or not receiving ARV therapy‡	2	1	2	1	*If on ARV, see also Drug Interactions.*							

Abbreviations: ARV = antiretroviral; C = continuation of contraceptive method; CHC = combined hormonal contraceptive (pill, patch, and ring); COC = combined oral contraceptive; Cu-IUD = copper intrauterine device; DMPA = depot medroxyprogesterone acetate; I = initiation of contraceptive method; LNG-IUD = levonorgestrel intrauterine device; NA = not applicable; POP = progestin-only pill; P/R = patch/ring; SSRI = selective serotonin reuptake inhibitor; STI = sexually transmitted infection; VTE = venous thromboembolism. ‡Condition associated with increased risk as a result of pregnancy. *Please see the complete guidance for a clarification to this classification: https://www.cdc.gov/contraception/hcp/usmec/.

CS 346094-A

Summary Chart of U.S. Medical Eligibility Criteria for Contraceptive Use (U.S. MEC)

Condition	Sub-Condition	Cu-IUD I	Cu-IUD C	LNG-IUD I	LNG-IUD C	Implant I	Implant C	DMPA I	DMPA C	POP I	POP C	CHC I	CHC C
**Hypertension**	a. Adequately controlled hypertension	1*		1*		1*		2*		1*		3*	
	b. Elevated blood pressure levels (*properly taken measurements*)												
	i. Systolic 140-159 or diastolic 90-99	1*		1*		1*		2*		1*		3*	
	ii. Systolic ≥160 or diastolic ≥100‡	1*		2*		2*		3*		2*		4*	
	c. Vascular disease	1*		2*		2*		3*		2*		4*	
**Inflammatory bowel disease**	(*ulcerative colitis or Crohn's disease*)	1		1		1		2		2		2/3*	
**Ischemic heart disease‡**	Current and history of	1		2	3	2	3	3		2	3	4	
**Liver tumors**	a. Benign												
	i. Focal nodular hyperplasia	1		2		2		2		2		2	
	ii. Hepatocellular adenoma‡	1		2		2		3		2		4	
	b. Malignant‡ (*hepatocellular carcinoma*)	1		3		3		3		3		4	
**Malaria**		1		1		1		1		1		1	
**Multiple risk factors for atherosclerotic cardiovascular disease**	(*e.g., older age, smoking, diabetes, hypertension, low HDL, high LDL, or high triglyceride levels*)	1		2		2*		3*		2*		3/4*	
**Multiple sclerosis**	a. Without prolonged immobility	1		1		1		2		1		1	
	b. With prolonged immobility	1		1		1		2		1		3	
**Obesity**	a. Body mass index (BMI) ≥30 kg/m²	1		1		1		1		1		2*	
	b. Menarche to <18 years and BMI ≥30 kg/m²	1		1		1		2		1		2*	
**Ovarian cancer‡**		1		1		1		1		1		1	
**Parity**	a. Nulliparous	2		2		1		1		1		1	
	b. Parous	1		1		1		1		1		1	
**Past ectopic pregnancy**		1		1		1		1		2		1	
**Pelvic inflammatory disease**	a. Current	4	2*	4	2*	1		1		1		1	
	b. Past												
	i. With subsequent pregnancy	1	1	1	1	1		1		1		1	
	ii. Without subsequent pregnancy	2	2	2	2	1		1		1		1	
**Peripartum cardiomyopathy‡**	a. Normal or mildly impaired cardiac function												
	i. <6 months	2		2		1		2		1		4	
	ii. ≥6 months	2		2		1		2		1		3	
	b. Moderately or severely impaired cardiac function	2		2		2		3		2		4	
**Postabortion** (*spontaneous or induced*)	a. First trimester abortion												
	i. Procedural (*surgical*)	1*		1*		1*		1*		1*		1*	
	ii. Medication	1*		1*		1*		1/2*		1*		1*	
	iii. Spontaneous abortion with no intervention	1*		1*		1*		1*		1*		1*	
	b. Second trimester abortion												
	i. Procedural (*surgical*)	2*		2*		1*		1*		1*		1*	
	ii. Medication	2*		2*		1*		1*		1*		1*	
	iii. Spontaneous abortion with no intervention	2*		2*		1*		1*		1*		1*	
	c. Immediate postseptic abortion	4		4		1*		1*		1*		1*	
**Postpartum** (*nonbreastfeeding*)	a. <21 days					1		2		1		4	
	b. 21 days to 42 days												
	i. With other risk factors for VTE					1		2		1		3*	
	ii. Without other risk factors for VTE					1		1		1		2	
	c. >42 days					1		1		1		1	
**Postpartum** (*including cesarean delivery, breastfeeding, or nonbreastfeeding*)	a. <10 minutes after delivery of the placenta	2*		2*									
	b. 10 minutes after delivery of the placenta to <4 weeks	2*		2*									
	c. ≥4 weeks	1*		1*									
	d. Postpartum sepsis	4		4									
**Pregnancy**		4*		4*		NA*		NA*		NA*		NA*	

Condition	Sub-Condition	Cu-IUD I	Cu-IUD C	LNG-IUD I	LNG-IUD C	Implant I	Implant C	DMPA I	DMPA C	POP I	POP C	CHC I	CHC C
**Rheumatoid arthritis**	a. Not on immunosuppressive therapy	1		1		1		2		1		2	
	b. On immunosuppressive therapy	2	1	2	1	1		2/3*		1		2	
**Schistosomiasis**	a. Uncomplicated	1		1		1		1		1		1	
	b. Fibrosis of the liver‡ (*if severe, see also Cirrhosis*)	1		1		1		1		1		1	
**Sexually transmitted infections (STIs)**	a. Current purulent cervicitis or chlamydial infection or gonococcal infection	4	2*	4	2*	1		1		1		1	
	b. Vaginitis (*including Trichomonas vaginalis and bacterial vaginosis*)	2	2	2	2	1		1		1		1	
	c. Other factors related to STIs	2*	2	2*	2	1		1		1		1	
**Sickle cell disease‡**		2		1		1		2/3*		1		4	
**Smoking**	a. Age <35	1		1		1		1		1		2	
	b. Age ≥35, <15 cigarettes/day	1		1		1		1		1		3	
	c. Age ≥35, ≥15 cigarettes/day	1		1		1		1		1		4	
**Solid organ transplantation‡**	a. No graft failure	1	1	1	1	2		2/3*		2		2*	
	b. Graft failure	2	1	2	1	2		2/3*		2		4	
**Stroke‡**	History of cerebrovascular accident	1		2		2	3	3		2	3	4	
**Superficial venous disorders**	a. Varicose veins	1		1		1		1		1		1	
	b. Superficial venous thrombosis (*acute or history*)	1		1		1		2		1		3*	
**Surgery**	a. Minor surgery without immobilization	1		1		1		1		1		1	
	b. Major surgery												
	i. Without prolonged immobilization	1		1		1		1		1		2	
	ii. With prolonged immobilization	1		1		1		2		1		4	
**Systemic lupus erythematosus‡**	a. Positive (or unknown) antiphospholipid antibodies	1*	1*	2*		2*		3*	3*	2*		4*	
	b. Severe thrombocytopenia	3*	2*	2*		2*		3*	2*	2*		2*	
	c. Immunosuppressive therapy	2*	1*	2*		2*		2*	2*	2*		2*	
	d. None of the above	1*	1*	2*		2*		2*	2*	2*		2*	
**Thalassemia**		2		1		1		1		1		1	
**Thrombophilia‡**		1*		2*		2*		3*		2*		4*	
**Thyroid disorders**	Simple goiter, hyperthyroid, or hypothyroid	1		1		1		1		1		1	
**Tuberculosis‡** (*see also Drug Interactions*)	a. Nonpelvic	1	1	1	1	1*		1*		1*		1*	
	b. Pelvic	4	3	4	3	1*		1*		1*		1*	
**Unexplained vaginal bleeding**	(suspicious for serious condition) before evaluation	4*	2*	4*	2*	3*		3*		2*		2*	
**Uterine fibroids**		2		2		1		1		1		1	
**Valvular heart disease**	a. Uncomplicated	1		1		1		1		1		2	
	b. Complicated‡	1		1		1		2		1		4	
**Vaginal bleeding patterns**	a. Irregular pattern without heavy bleeding	1		1	1	2		2		2		1	
	b. Heavy or prolonged bleeding	2*		1*	2*	2*		2*		2*		1*	
**Viral hepatitis**	a. Acute or flare	1		1		1		1		1		3/4*	2
	b. Chronic	1		1		1		1		1		1	1
**Drug Interactions**													
**Antiretrovirals (ARVs) used for prevention (PrEP) or treatment of HIV‡**	Fosamprenavir (FPV)<br>All other ARVs are 1 or 2 for all methods	1/2*	1*	1/2*	1*	2*		2*		2*		3*	
**Anticonvulsant therapy**	a. Certain anticonvulsants (phenytoin, carbamazepine, barbiturates, primidone, topiramate, oxcarbazepine)	1		1		2*		1*		3*		3*	
	b. Lamotrigine	1		1		1		1		1		3*	
**Antimicrobial therapy**	a. Broad-spectrum antibiotics	1		1		1		1		1		1	
	b. Antifungals	1		1		1		1		1		1	
	c. Antiparasitics	1		1		1		1		1		1	
	d. Rifampin or rifabutin therapy	1		1		2*		1*		3*		3*	
**SSRIs**		1		1		1		1		1		1	
**St. John's wort**		1		1		2		1		2		2	

CS 349509-A
