# Supplementary material for: Benefits of Hormonal Contraception Across the Lifespan: A Case-Based, Interactive Curriculum
Source: MedEdPORTAL. 2025 Apr 4;21:11512. doi: 10.15766/mep_2374-8265.11512 (PMC11968450; doi:10.15766/mep_2374-8265.11512)
Supplement: Supplementary file 1 — Student Guide and Case 1.docxCase 2.docxCase 3.docxCDC Eligibility Criteria for Contraceptive Use.pdfBN How Well Does Birth Control Work.pdfRHAP Birth Control Across the Gender Spectrum.pdfCounseling for the Hormones Found in Contraceptives.pptxCase-Based Collaborative Learning.pptxFaculty Guide.docxLongitudinal Assessment Questions.docx [file mep_2374-8265.11512-s001.zip › G. Counseling for the Hormones Found in Contraceptives.pptx]

## Slide 1
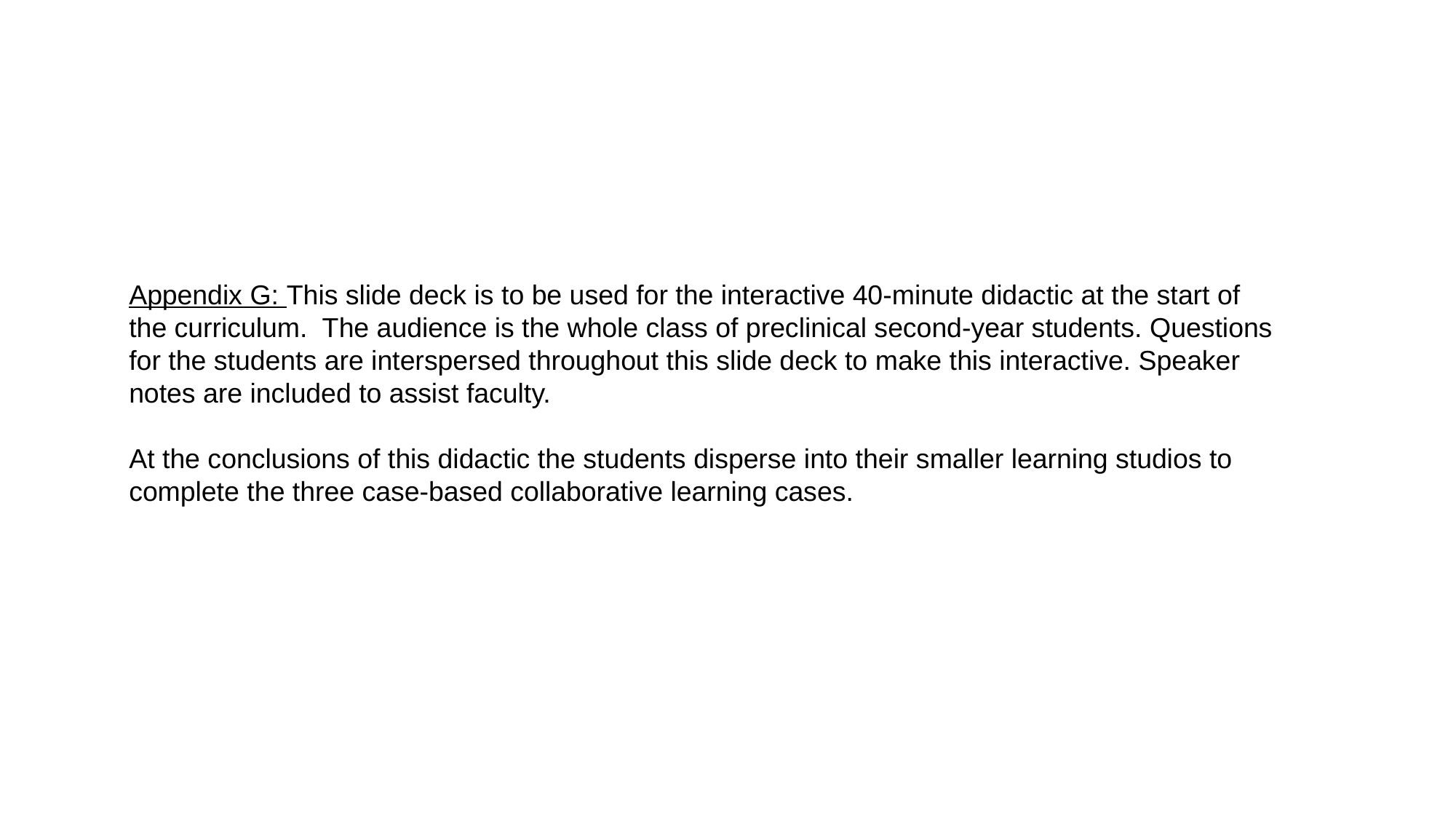

Appendix G: This slide deck is to be used for the interactive 40-minute didactic at the start of the curriculum. The audience is the whole class of preclinical second-year students. Questions for the students are interspersed throughout this slide deck to make this interactive. Speaker notes are included to assist faculty.
At the conclusions of this didactic the students disperse into their smaller learning studios to complete the three case-based collaborative learning cases.

## Slide 2
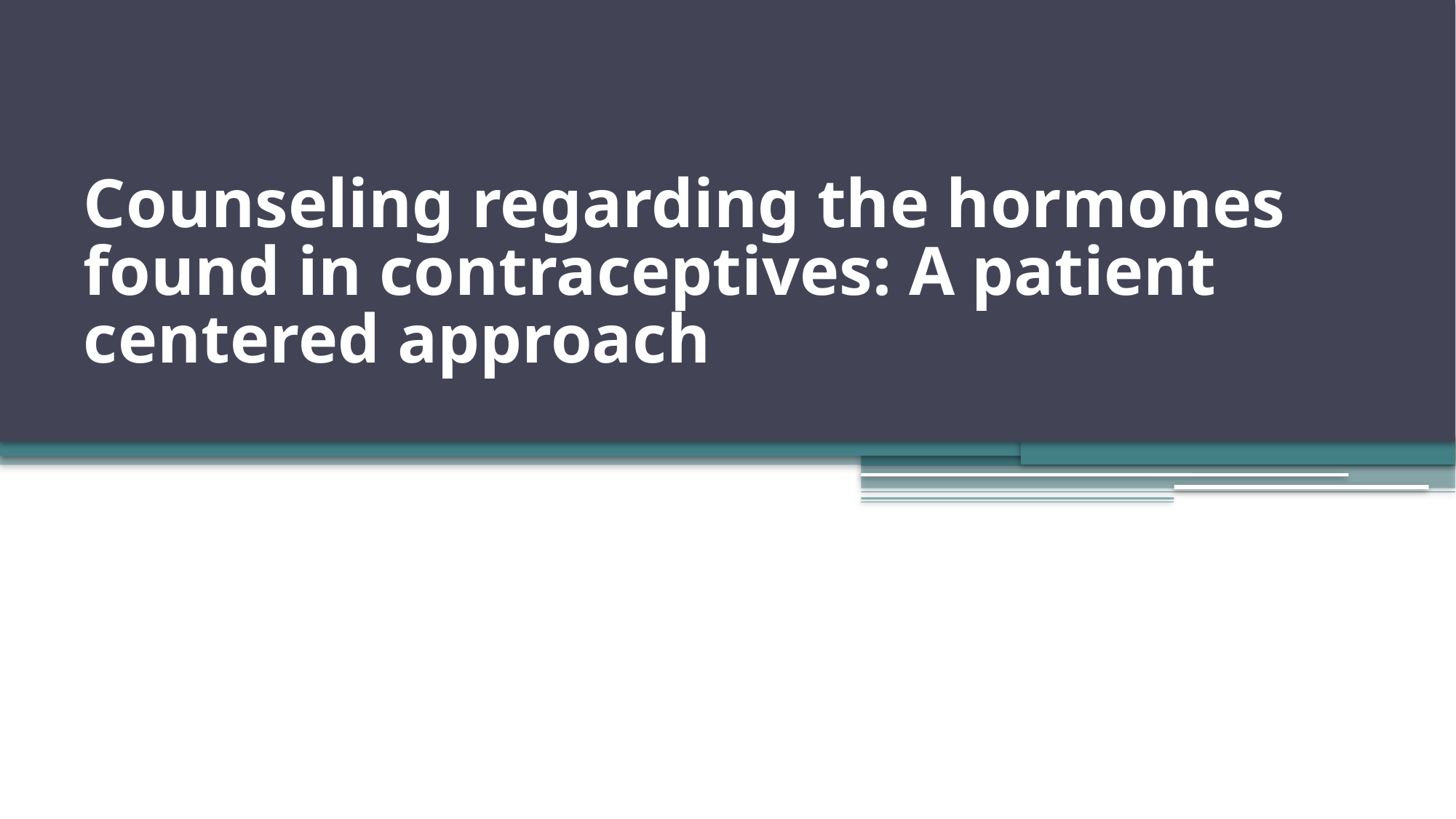

# Counseling regarding the hormones found in contraceptives: A patient centered approach

## Slide 3
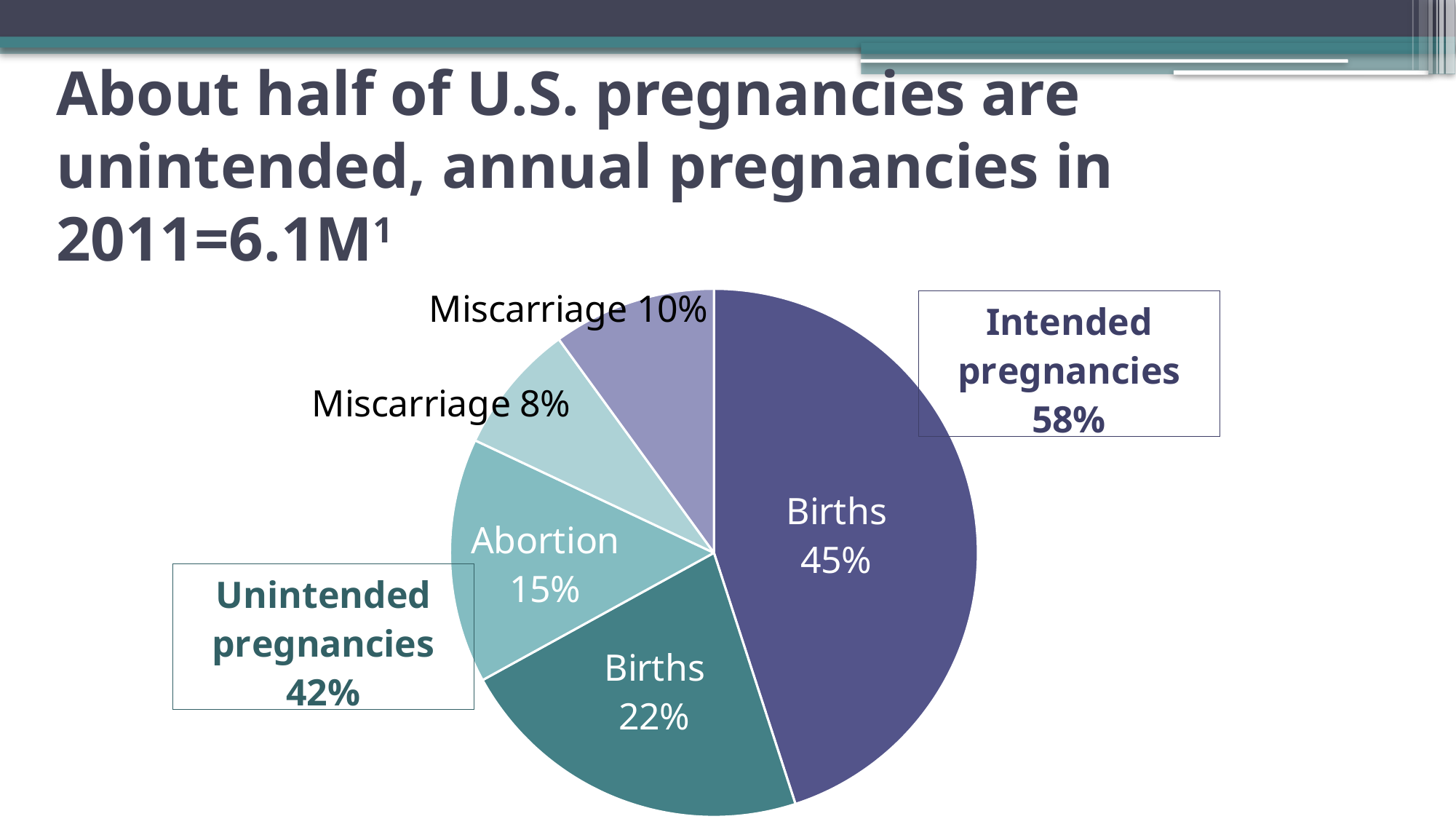

# About half of U.S. pregnancies are unintended, annual pregnancies in 2011=6.1M1
### Chart
| Category | Column1 |
|---|---|
| | 45.0 |
| | 22.0 |
| | 15.0 |
| | 8.0 |
| | 10.0 |

## Slide 4
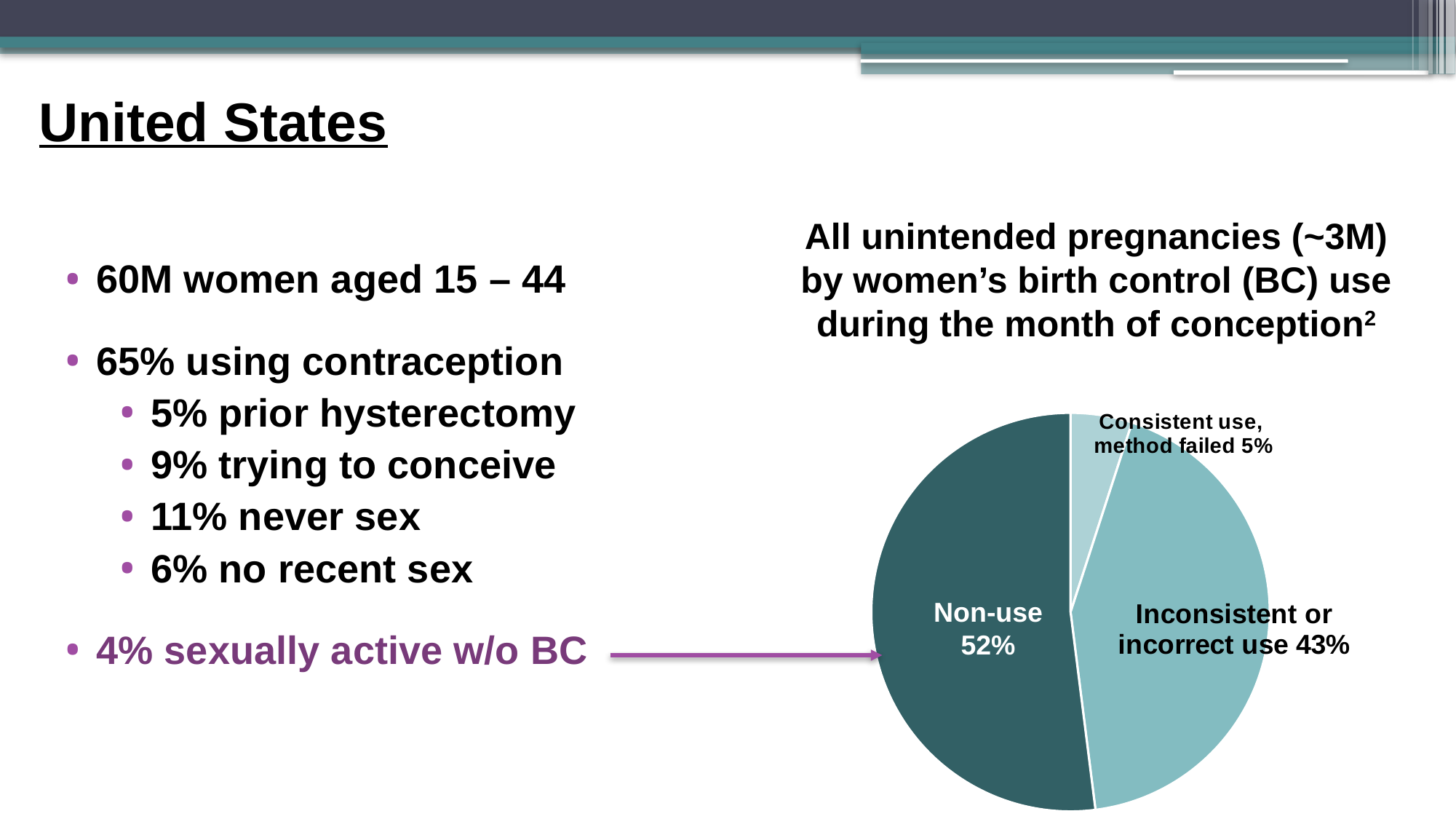

United States
All unintended pregnancies (~3M) by women’s birth control (BC) use during the month of conception2
60M women aged 15 – 44
65% using contraception
5% prior hysterectomy
9% trying to conceive
11% never sex
6% no recent sex
4% sexually active w/o BC
### Chart
| Category | Column1 |
|---|---|
| | 5.0 |
| | 43.0 |
| | 52.0 |
| | None |Non-use
52%

## Slide 5
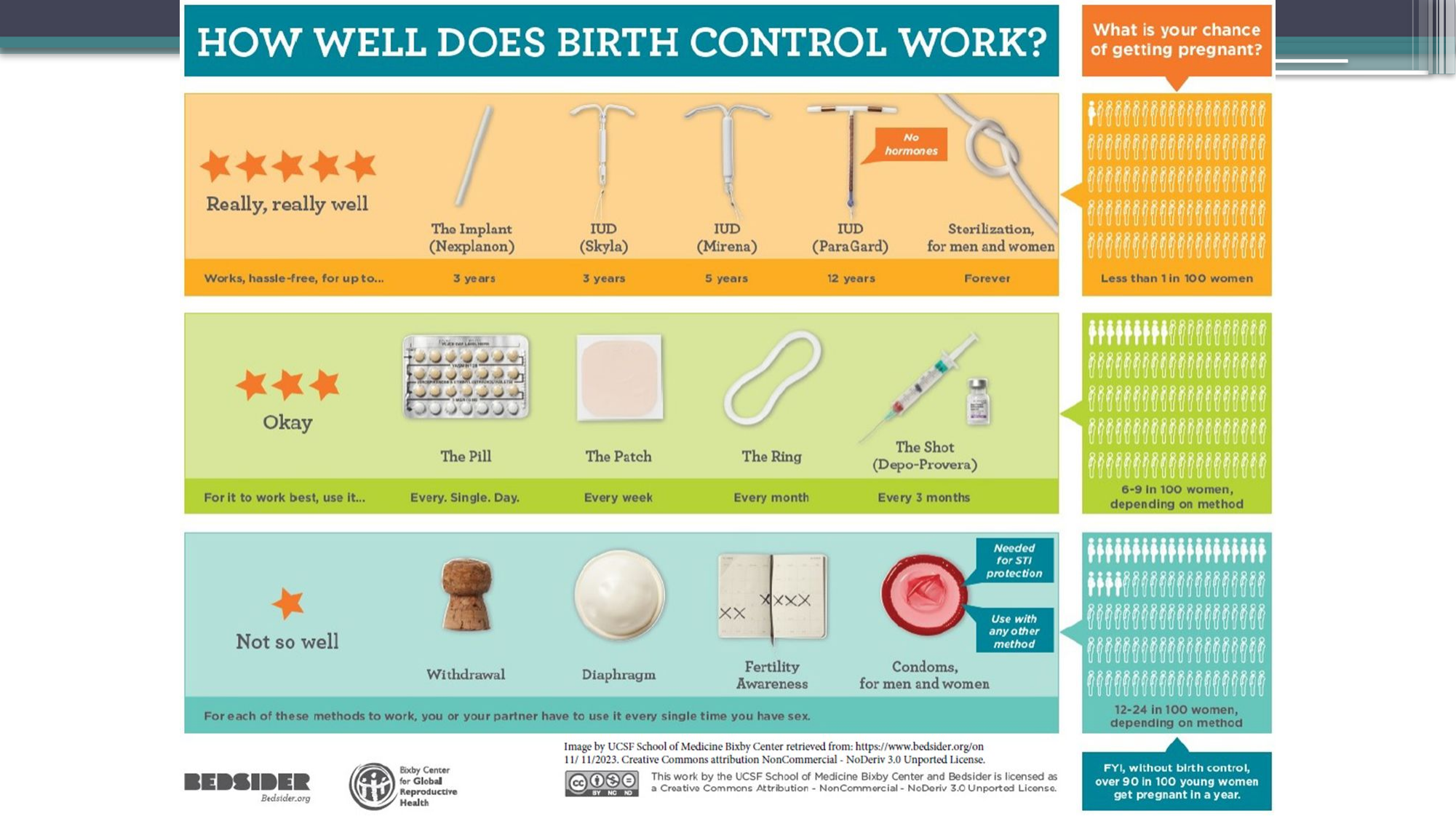

## Slide 6
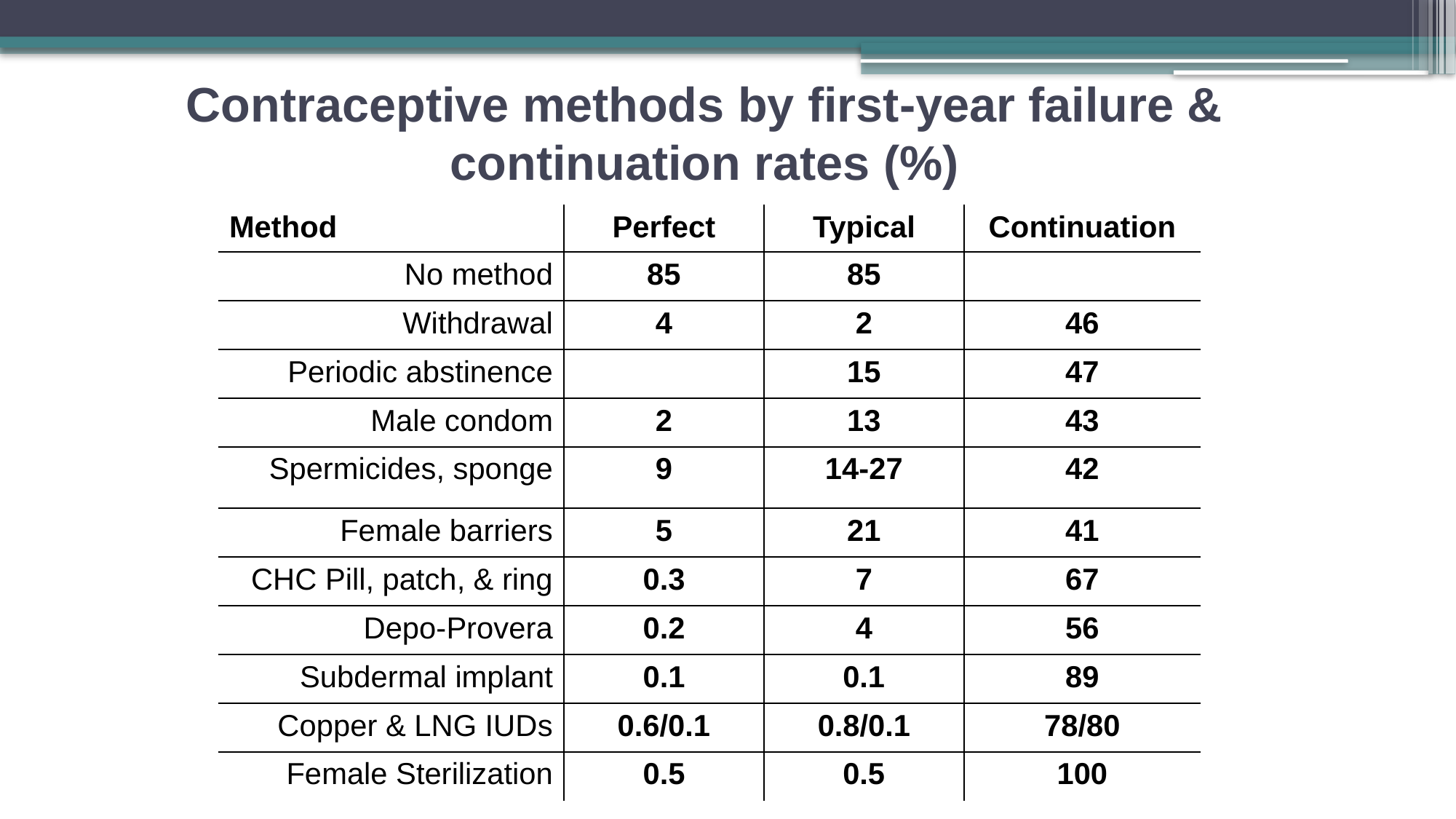

Contraceptive methods by first-year failure & continuation rates (%)
| Method | Perfect | Typical | Continuation |
| --- | --- | --- | --- |
| No method | 85 | 85 | |
| Withdrawal | 4 | 2 | 46 |
| Periodic abstinence | | 15 | 47 |
| Male condom | 2 | 13 | 43 |
| Spermicides, sponge | 9 | 14-27 | 42 |
| Female barriers | 5 | 21 | 41 |
| CHC Pill, patch, & ring | 0.3 | 7 | 67 |
| Depo-Provera | 0.2 | 4 | 56 |
| Subdermal implant | 0.1 | 0.1 | 89 |
| Copper & LNG IUDs | 0.6/0.1 | 0.8/0.1 | 78/80 |
| Female Sterilization | 0.5 | 0.5 | 100 |

## Slide 7
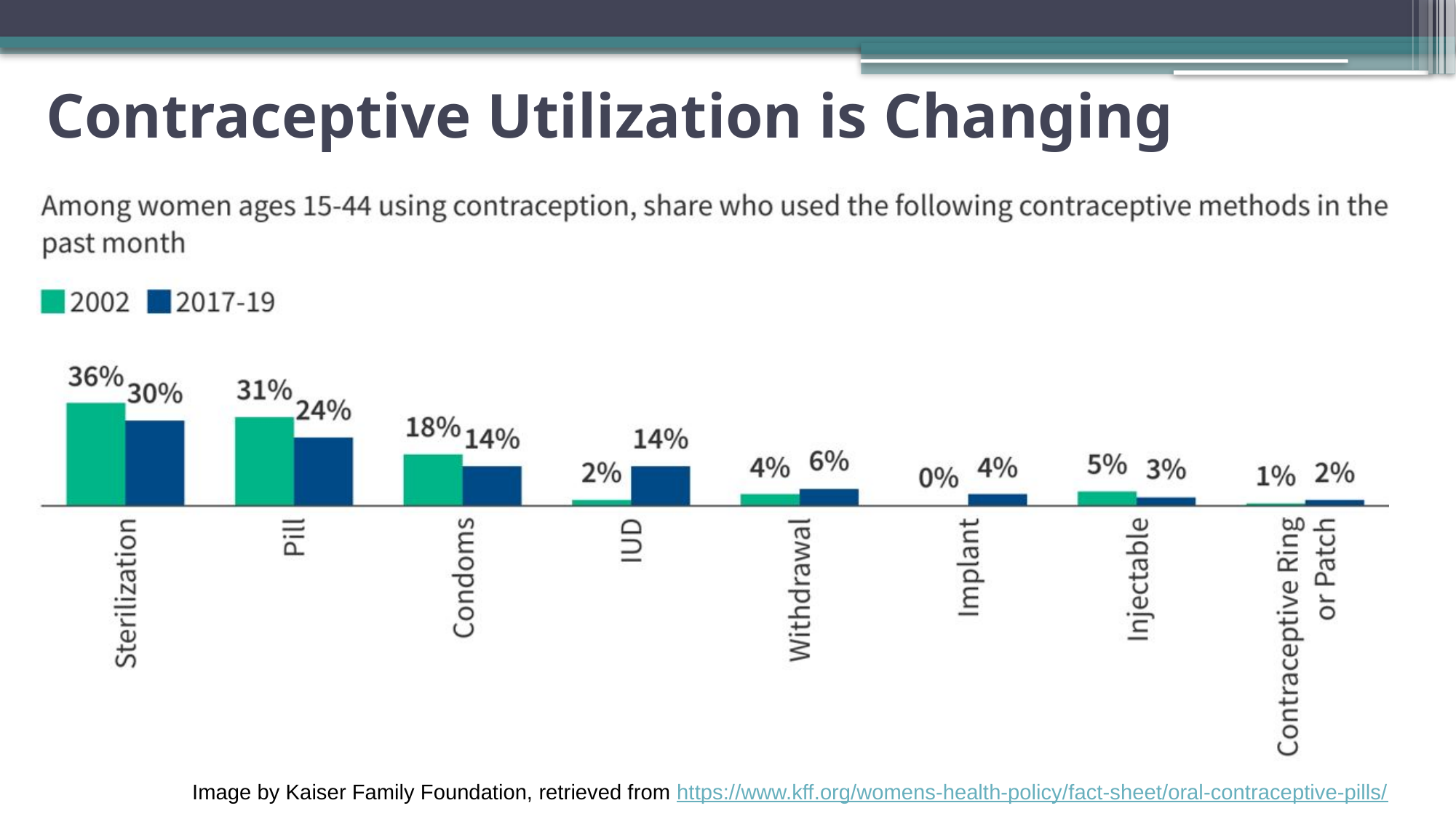

# Contraceptive Utilization is Changing
Image by Kaiser Family Foundation, retrieved from https://www.kff.org/womens-health-policy/fact-sheet/oral-contraceptive-pills/

## Slide 8
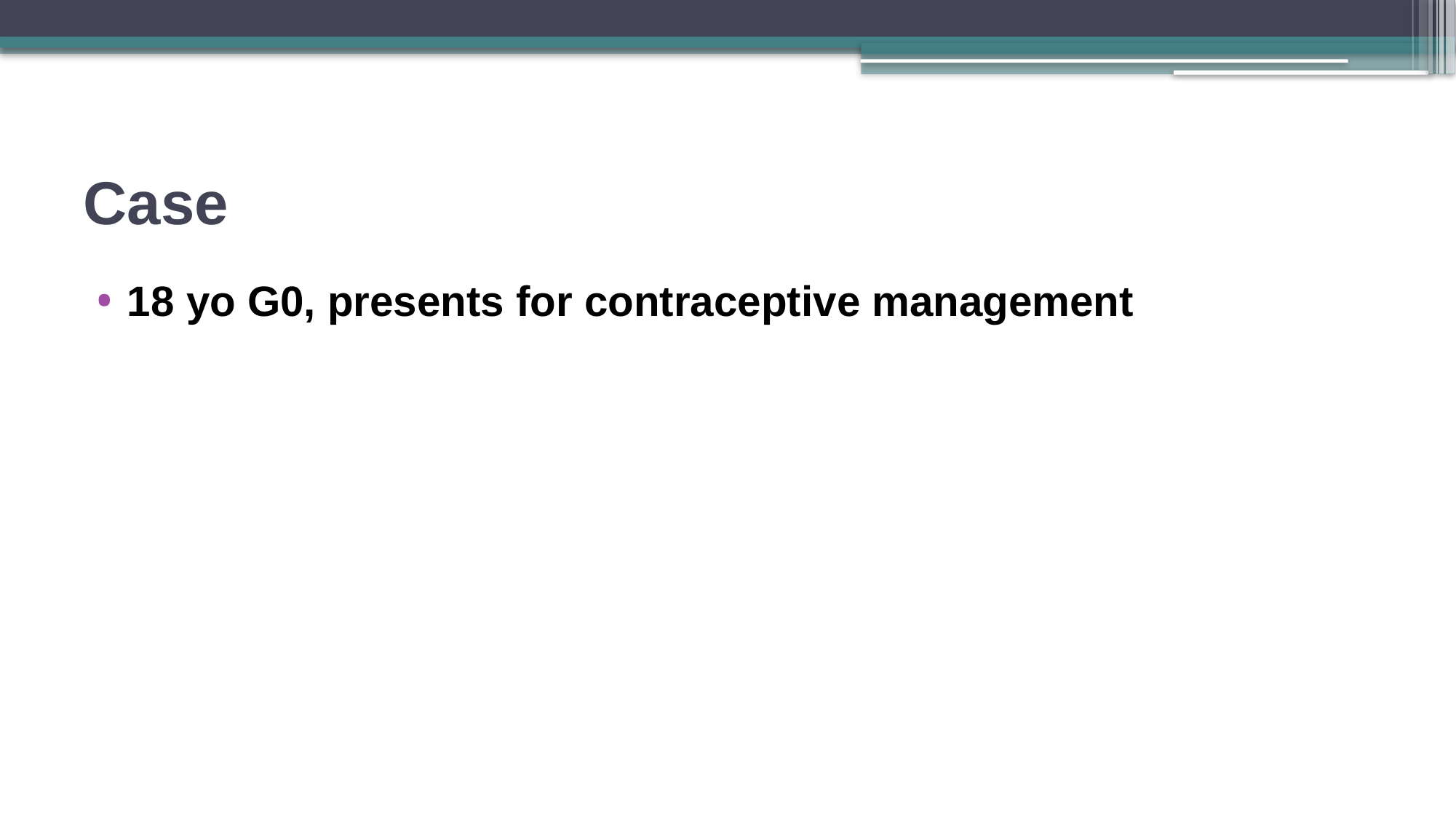

# Case
18 yo G0, presents for contraceptive management

## Slide 9
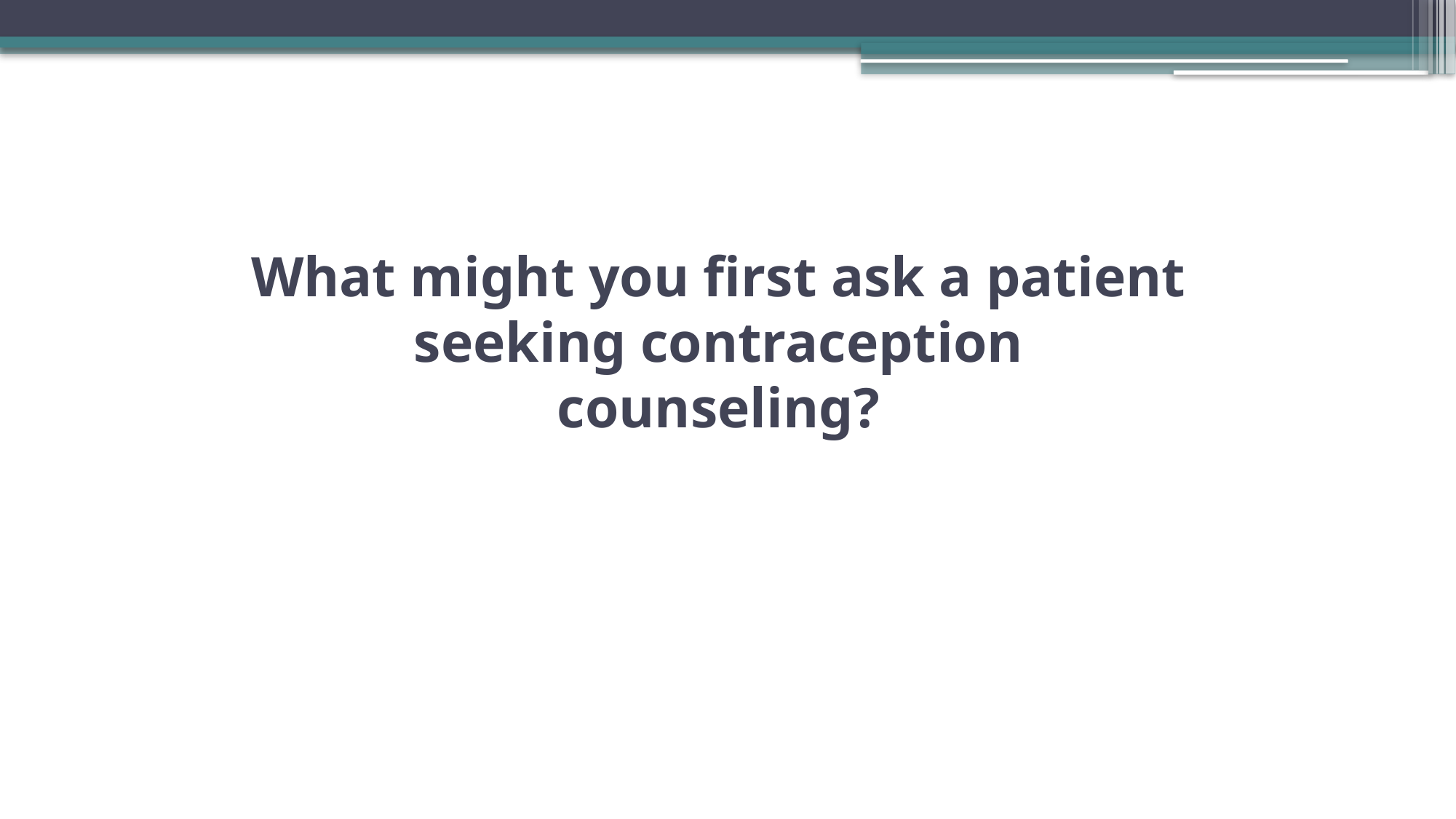

What might you first ask a patient seeking contraception counseling?

## Slide 10
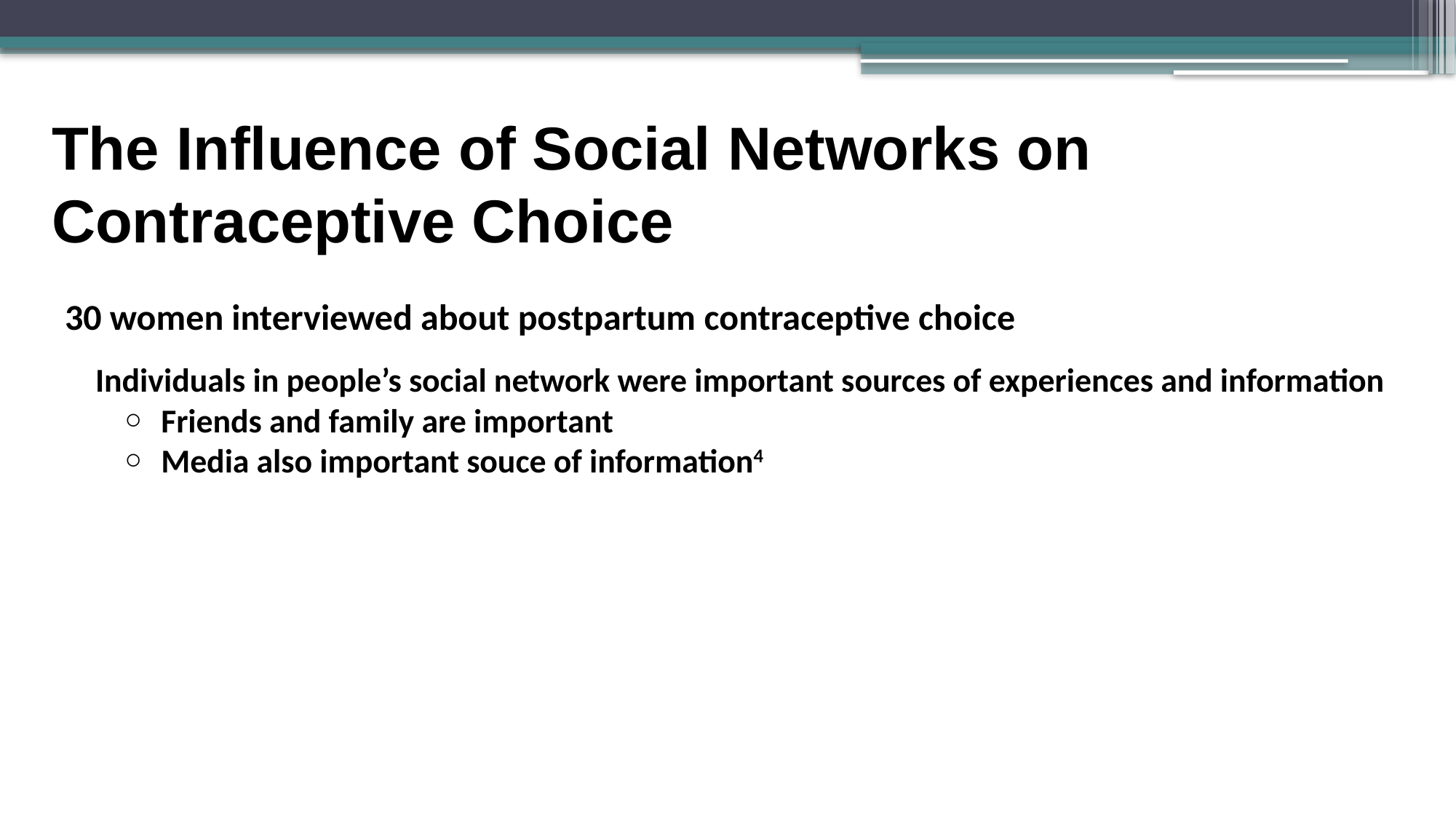

# The Influence of Social Networks on Contraceptive Choice
30 women interviewed about postpartum contraceptive choice
	Individuals in people’s social network were important sources of experiences and information
Friends and family are important
Media also important souce of information4

## Slide 11
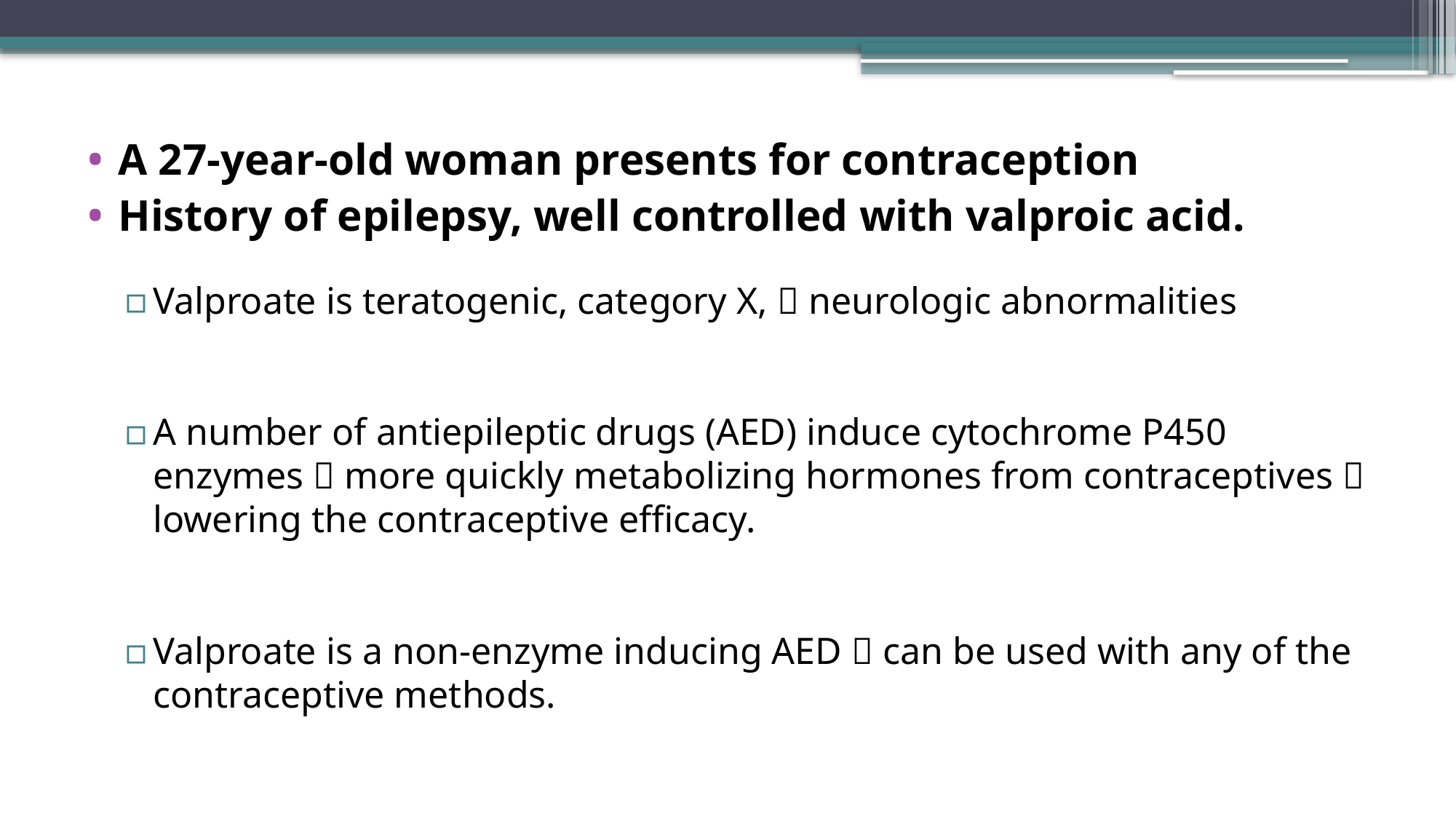

A 27-year-old woman presents for contraception
History of epilepsy, well controlled with valproic acid.
Valproate is teratogenic, category X,  neurologic abnormalities
A number of antiepileptic drugs (AED) induce cytochrome P450 enzymes  more quickly metabolizing hormones from contraceptives  lowering the contraceptive efficacy.
Valproate is a non-enzyme inducing AED  can be used with any of the contraceptive methods.

## Slide 12
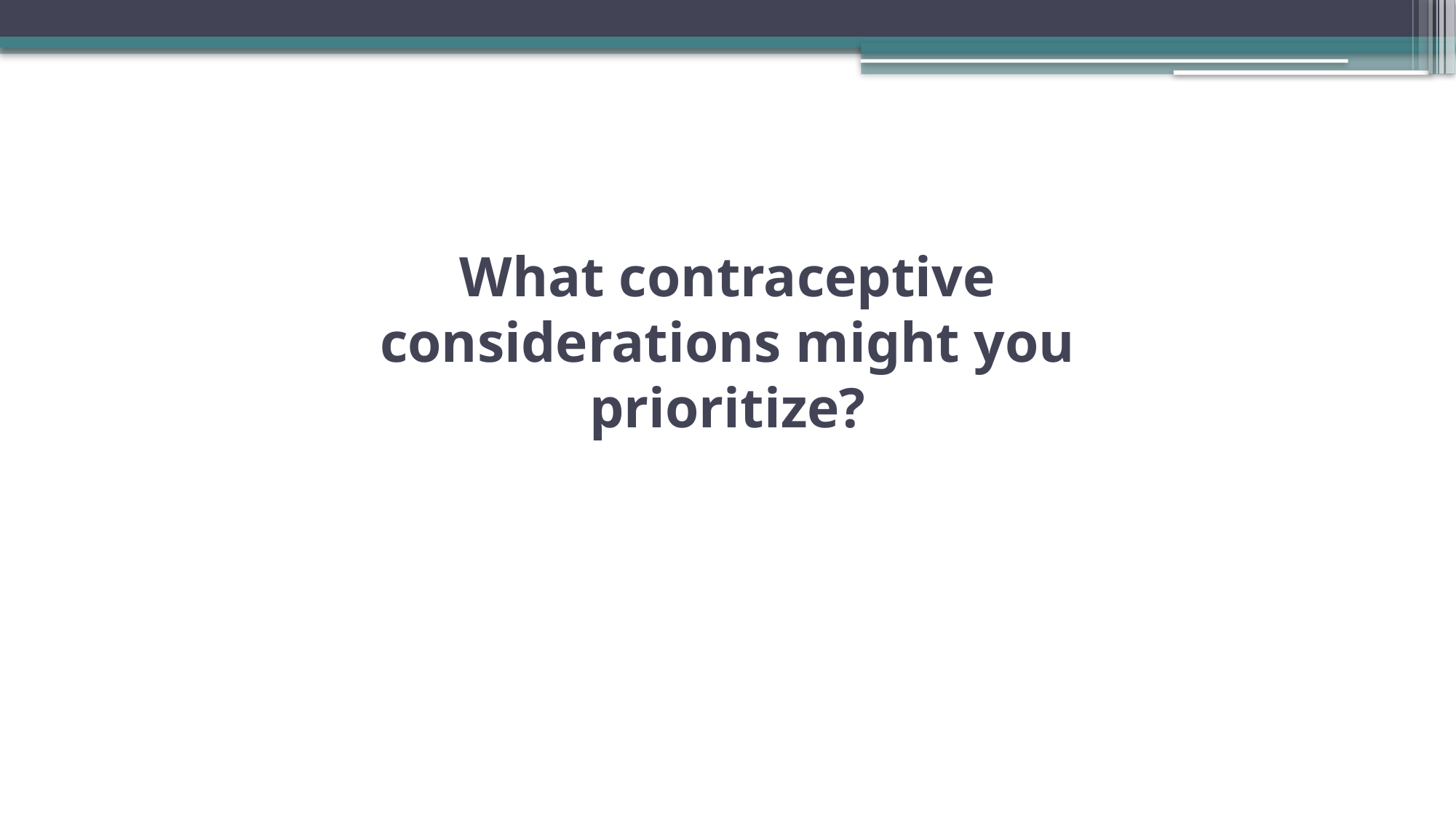

What contraceptive considerations might you prioritize?

## Slide 13
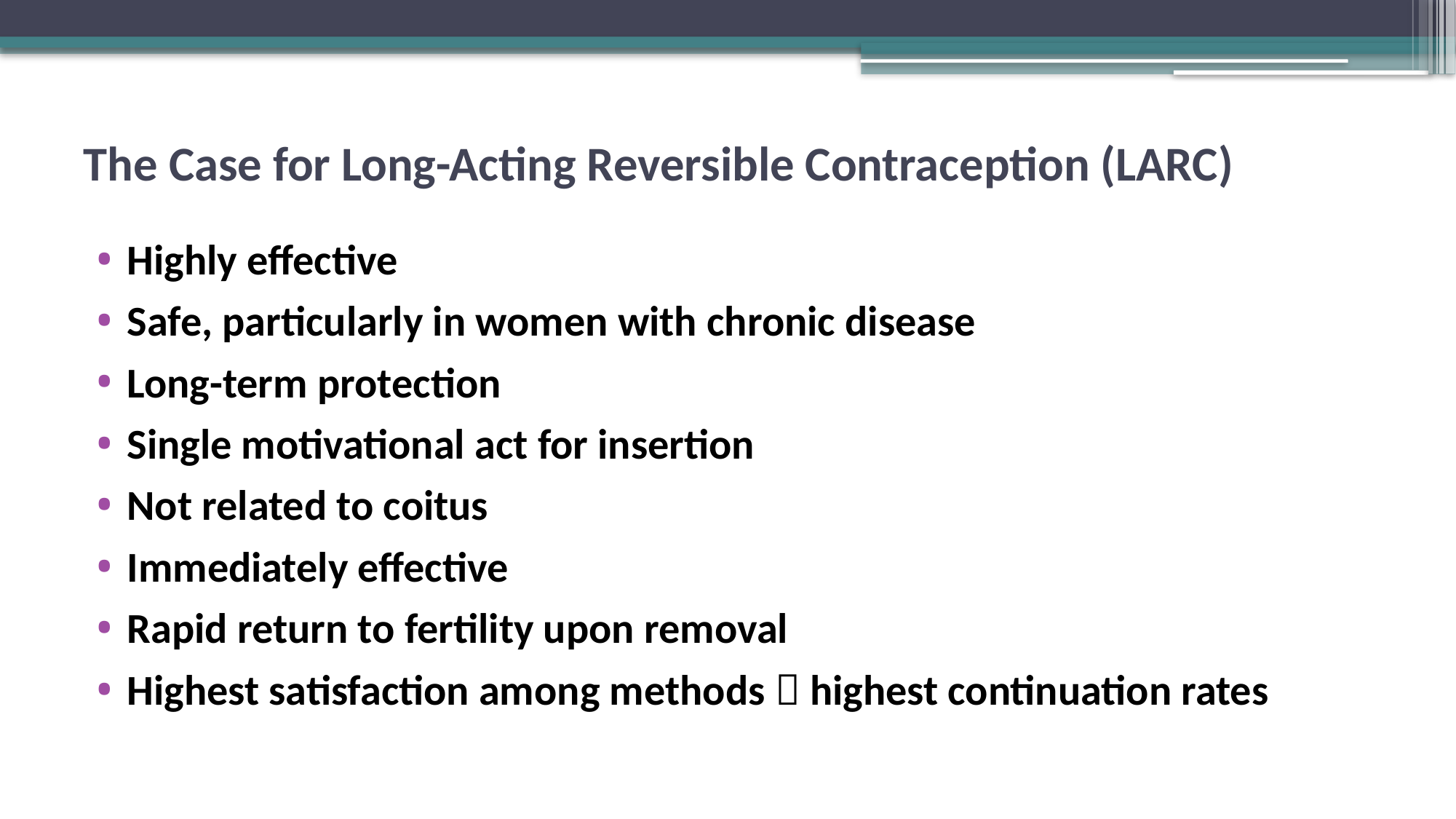

The Case for Long-Acting Reversible Contraception (LARC)
Highly effective
Safe, particularly in women with chronic disease
Long-term protection
Single motivational act for insertion
Not related to coitus
Immediately effective
Rapid return to fertility upon removal
Highest satisfaction among methods  highest continuation rates

## Slide 14
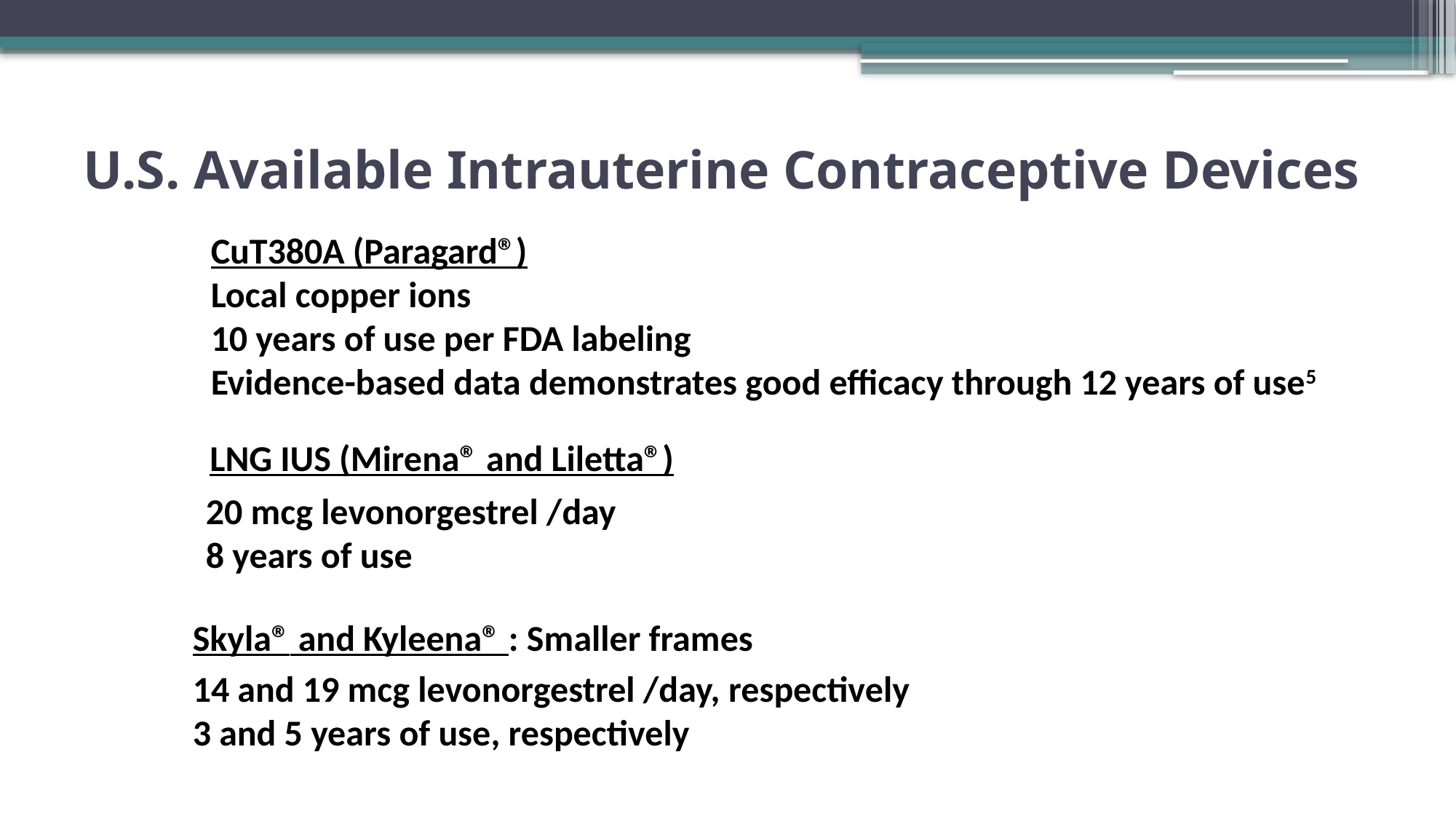

U.S. Available Intrauterine Contraceptive Devices
CuT380A (Paragard®)
Local copper ions
10 years of use per FDA labeling
Evidence-based data demonstrates good efficacy through 12 years of use5
LNG IUS (Mirena® and Liletta®)
20 mcg levonorgestrel /day
8 years of use
Skyla® and Kyleena® : Smaller frames
14 and 19 mcg levonorgestrel /day, respectively
3 and 5 years of use, respectively

## Slide 15
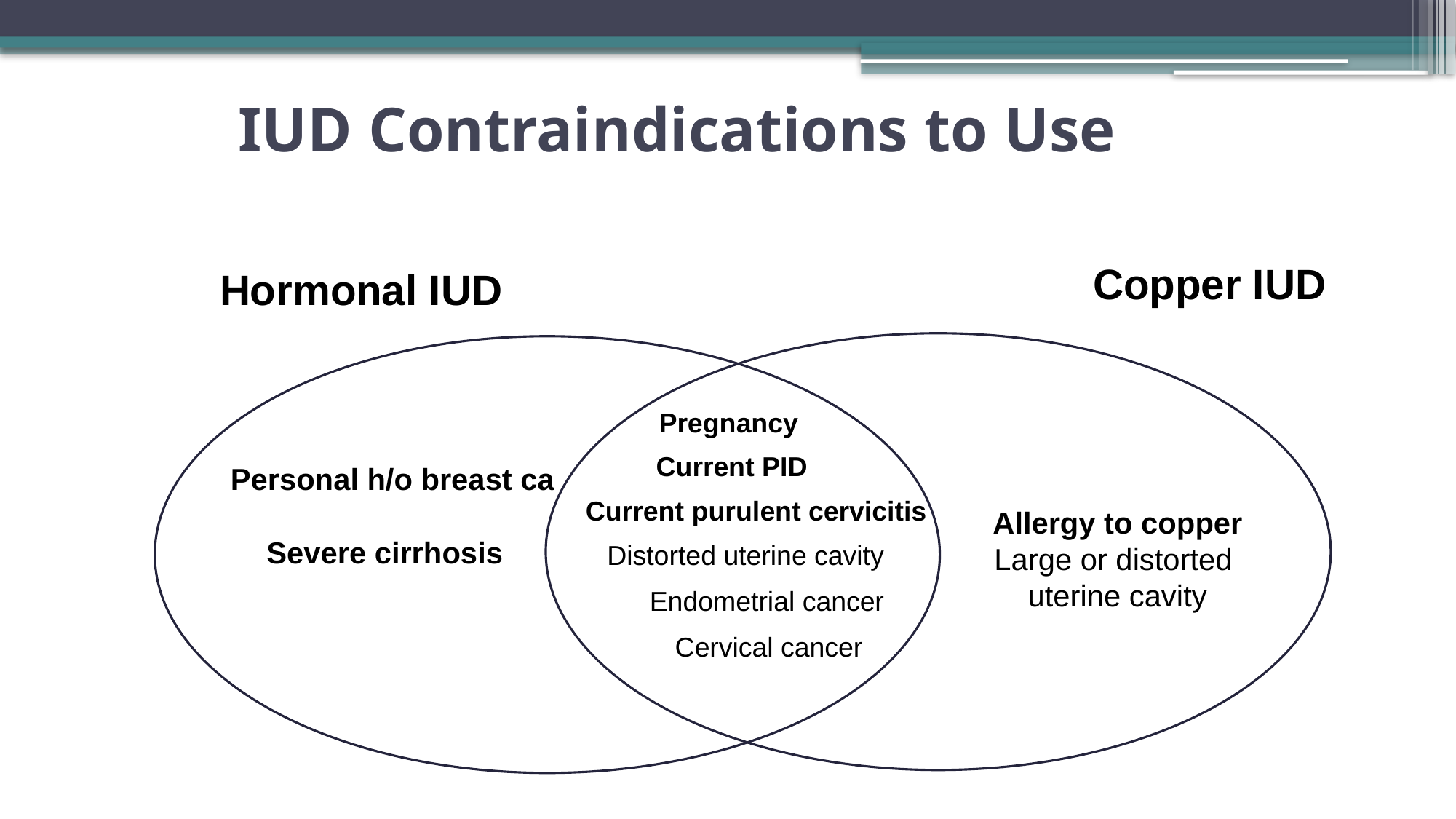

# IUD Contraindications to Use
Copper IUD
Hormonal IUD
Pregnancy
Current PID
Personal h/o breast ca
Current purulent cervicitis
Allergy to copper
Large or distorted
uterine cavity
Severe cirrhosis
Distorted uterine cavity
Endometrial cancer
Cervical cancer

## Slide 16
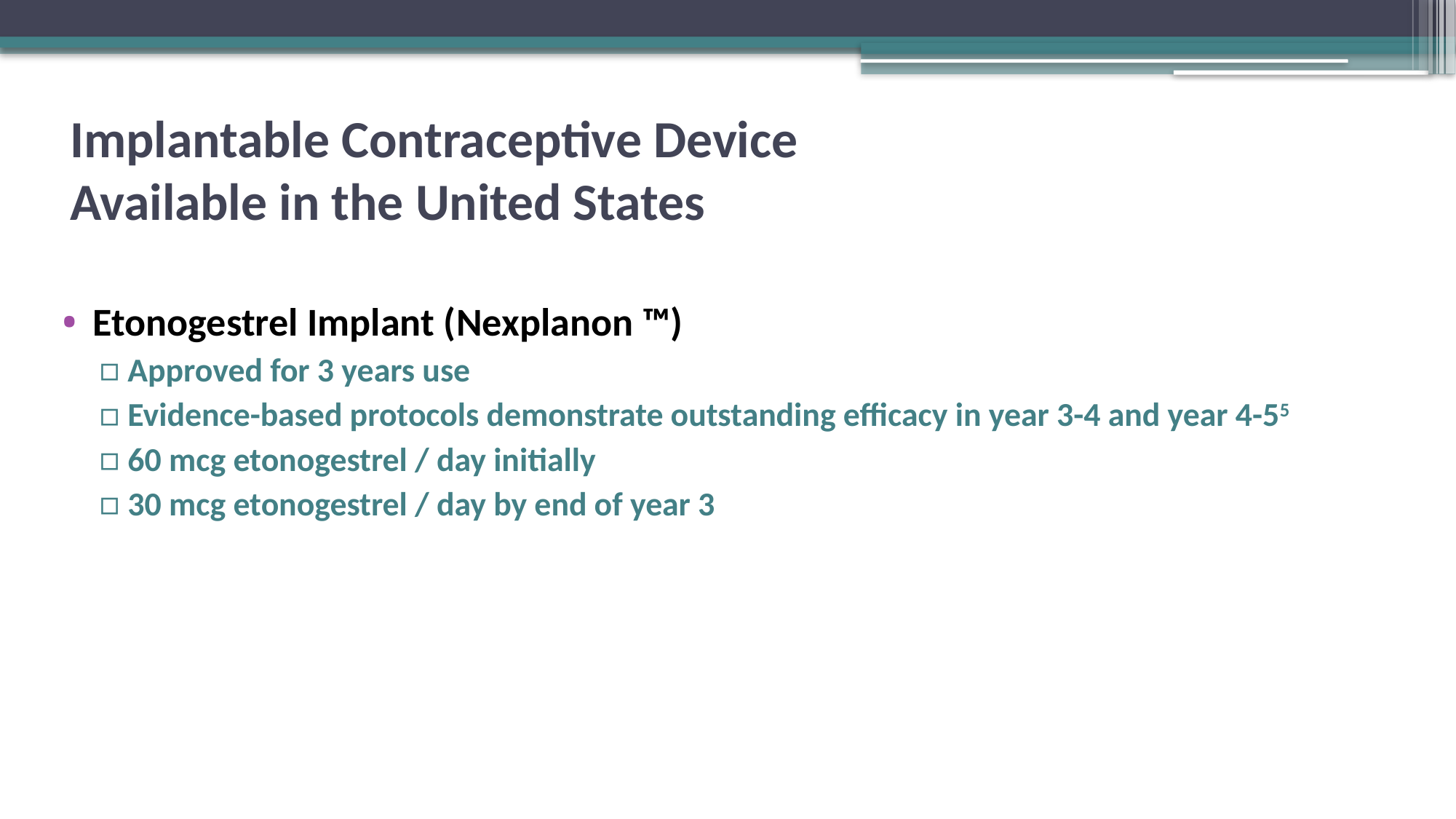

# Implantable Contraceptive Device Available in the United States
Etonogestrel Implant (Nexplanon ™)
Approved for 3 years use
Evidence-based protocols demonstrate outstanding efficacy in year 3-4 and year 4-55
60 mcg etonogestrel / day initially
30 mcg etonogestrel / day by end of year 3

## Slide 17
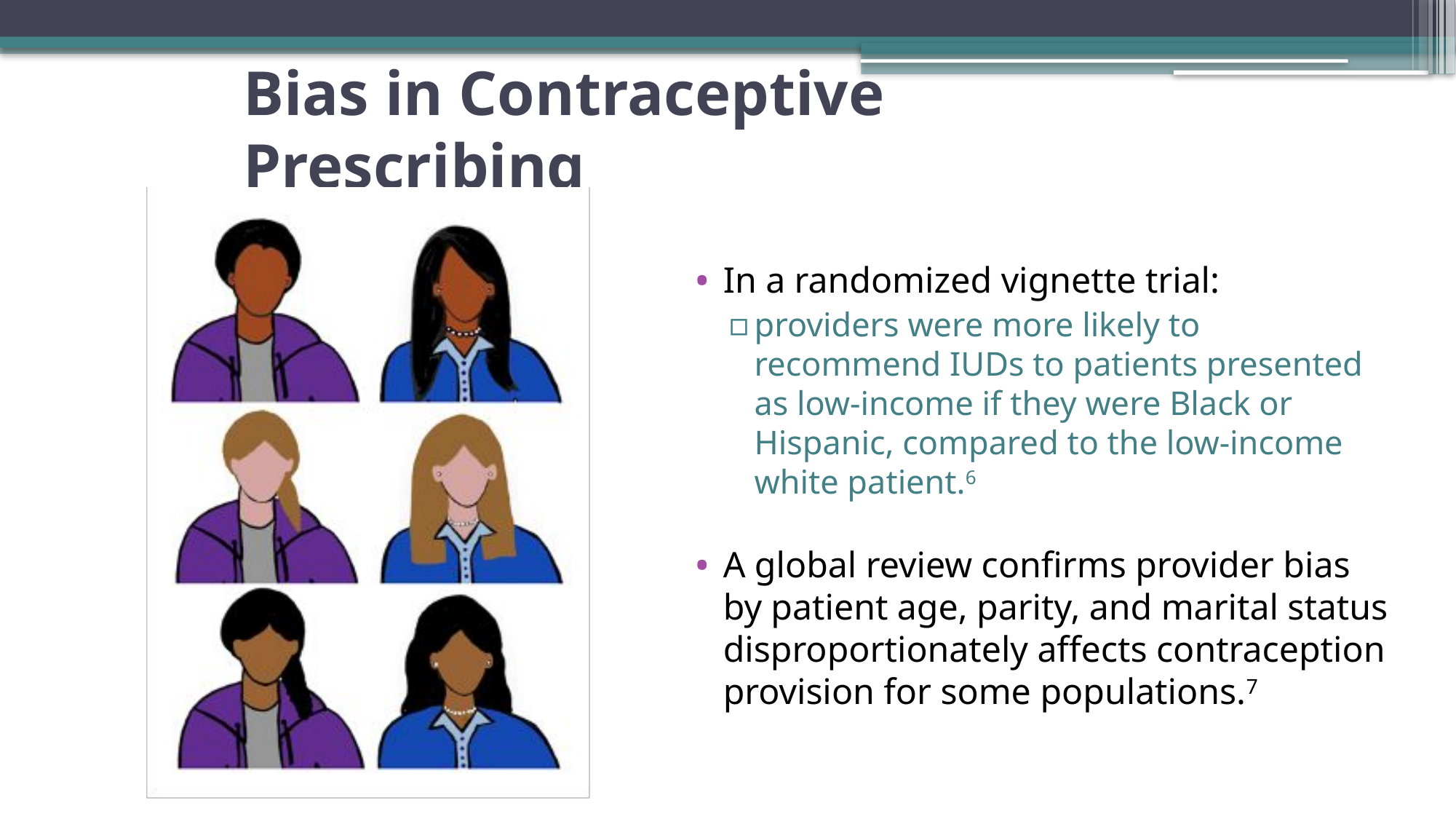

# Bias in Contraceptive Prescribing
In a randomized vignette trial:
providers were more likely to recommend IUDs to patients presented as low-income if they were Black or Hispanic, compared to the low-income white patient.6
A global review confirms provider bias by patient age, parity, and marital status disproportionately affects contraception provision for some populations.7

## Slide 18
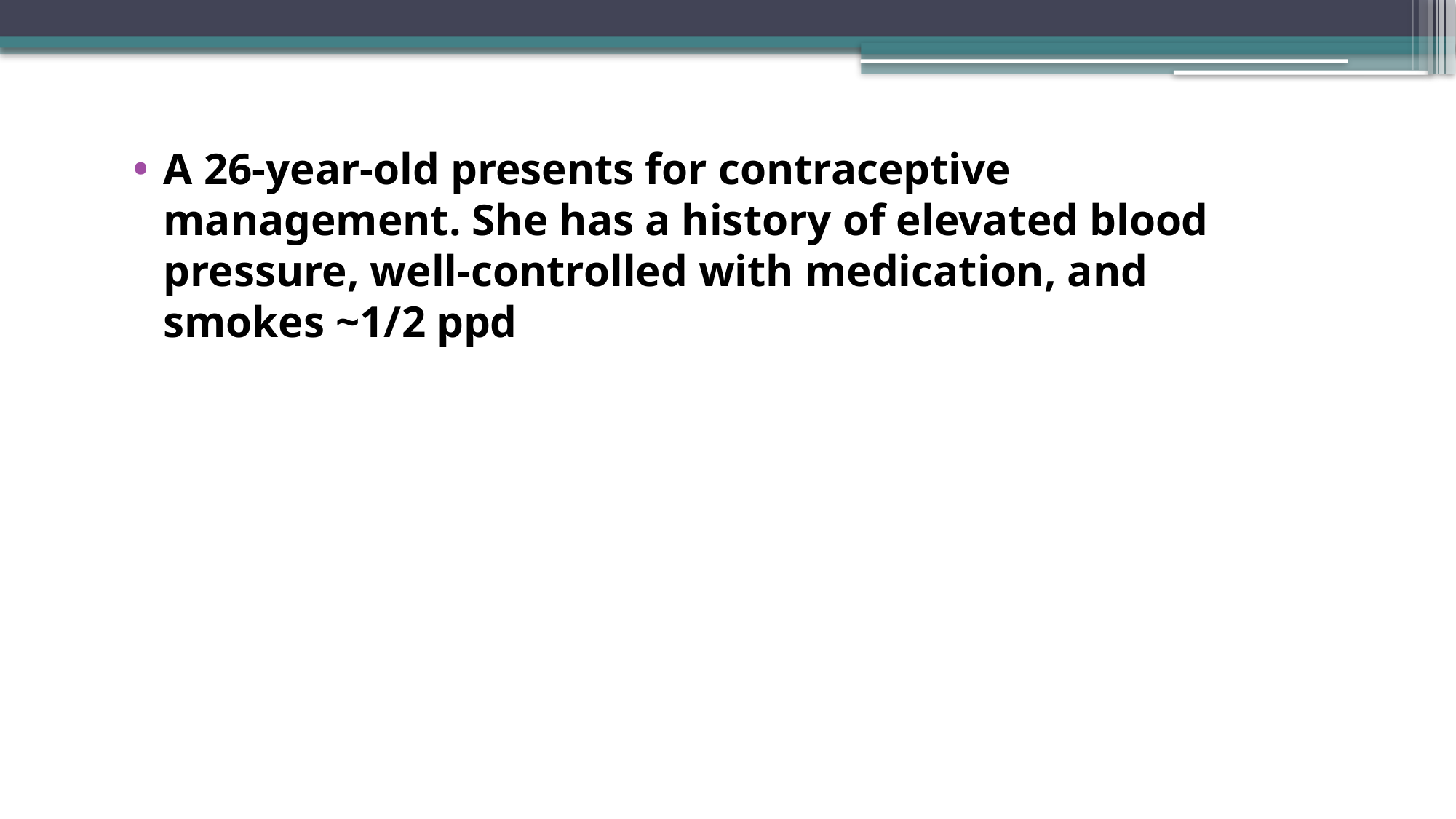

A 26-year-old presents for contraceptive management. She has a history of elevated blood pressure, well-controlled with medication, and smokes ~1/2 ppd

## Slide 19
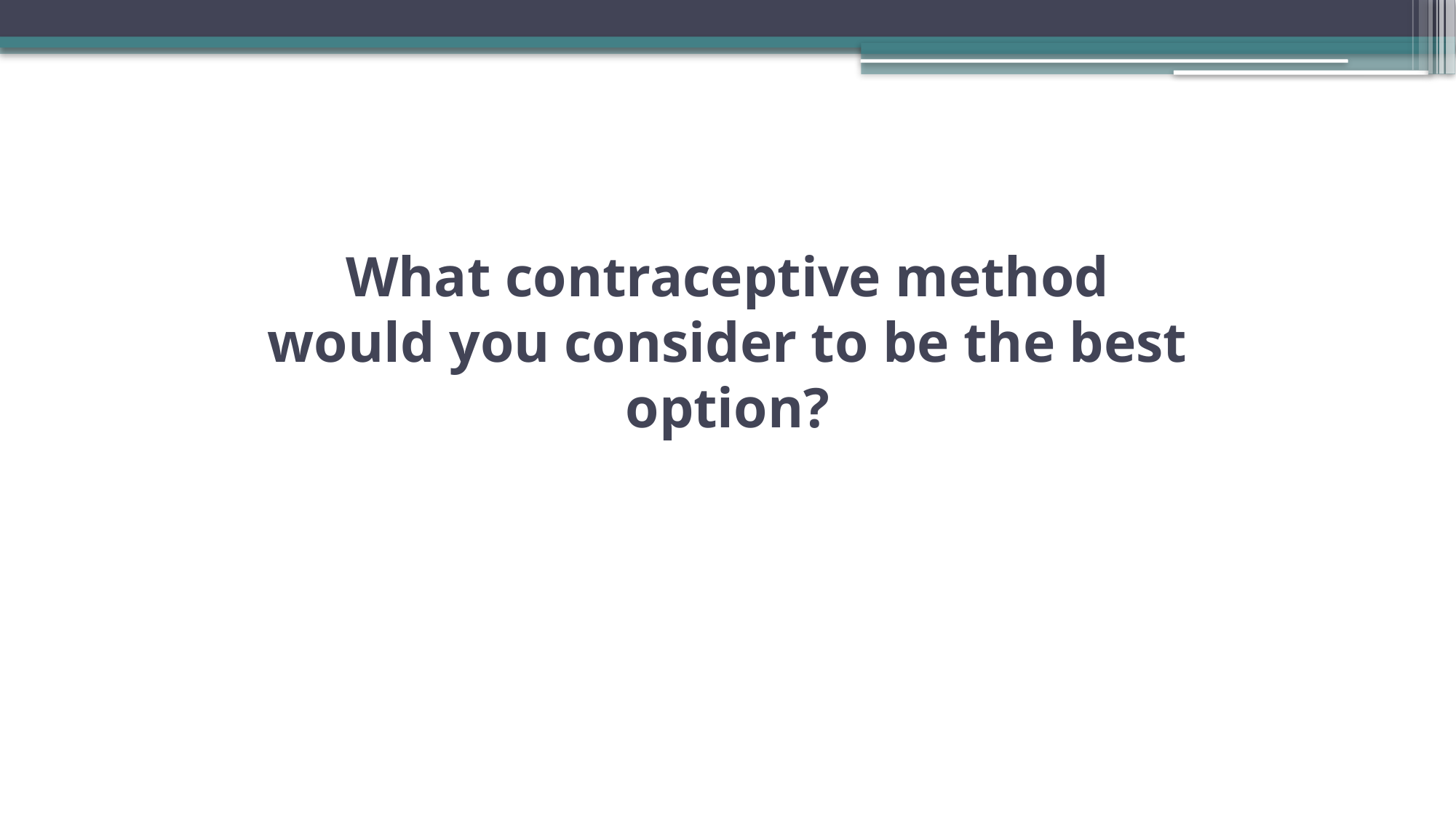

What contraceptive method would you consider to be the best option?

## Slide 20
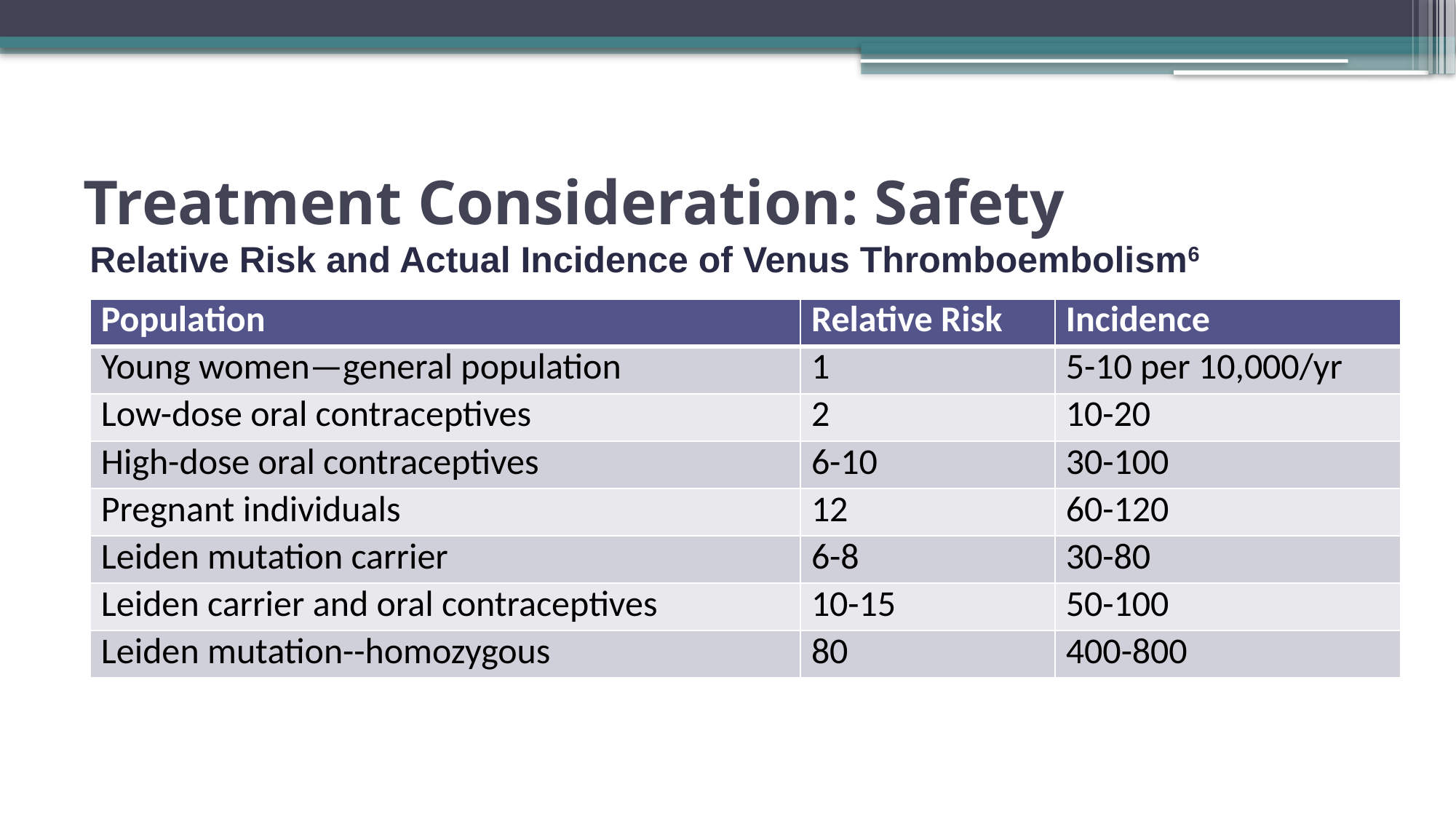

# Treatment Consideration: Safety
Relative Risk and Actual Incidence of Venus Thromboembolism6
| Population | Relative Risk | Incidence |
| --- | --- | --- |
| Young women—general population | 1 | 5-10 per 10,000/yr |
| Low-dose oral contraceptives | 2 | 10-20 |
| High-dose oral contraceptives | 6-10 | 30-100 |
| Pregnant individuals | 12 | 60-120 |
| Leiden mutation carrier | 6-8 | 30-80 |
| Leiden carrier and oral contraceptives | 10-15 | 50-100 |
| Leiden mutation--homozygous | 80 | 400-800 |

## Slide 21
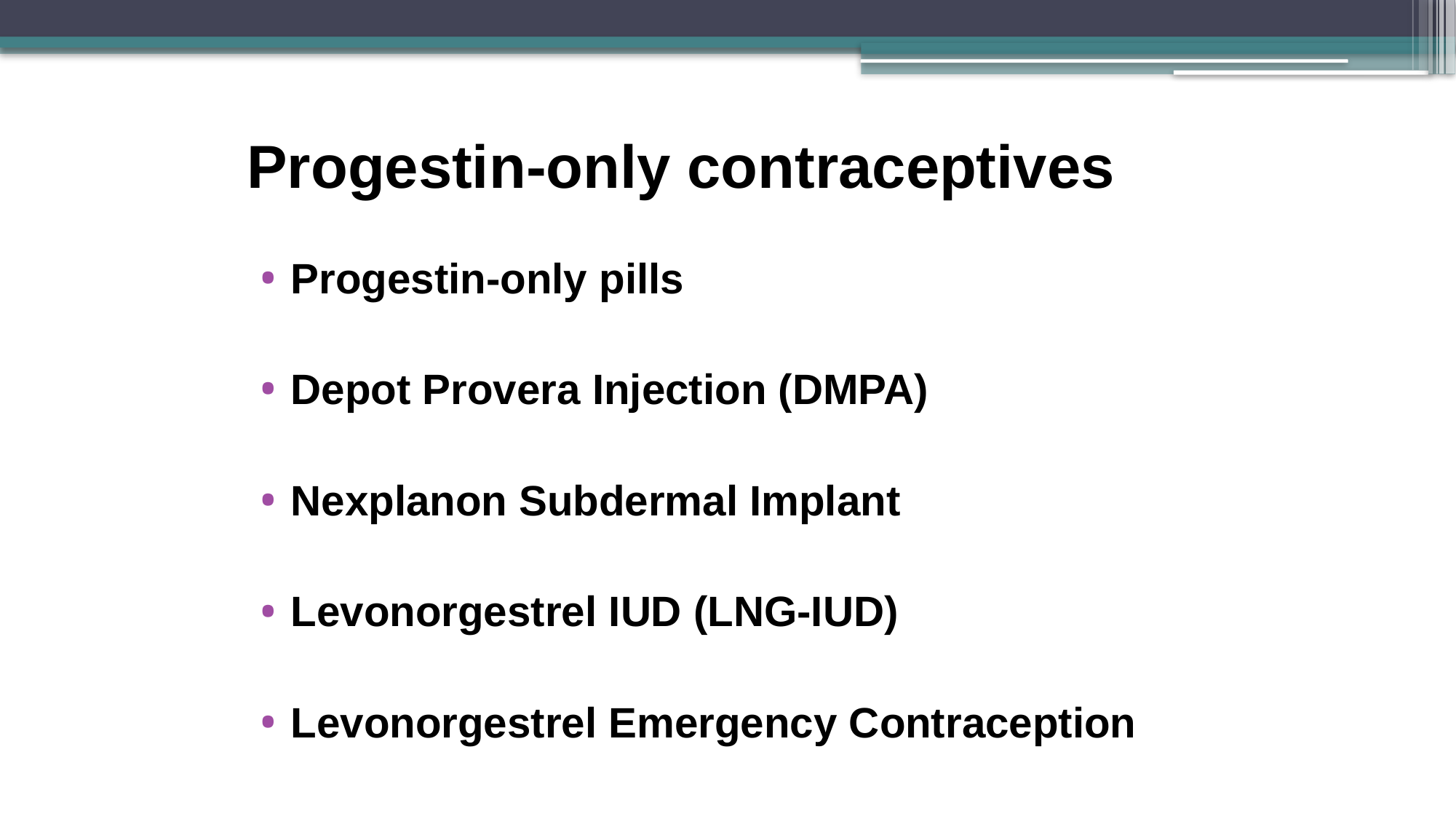

# Progestin-only contraceptives
Progestin-only pills
Depot Provera Injection (DMPA)
Nexplanon Subdermal Implant
Levonorgestrel IUD (LNG-IUD)
Levonorgestrel Emergency Contraception

## Slide 22
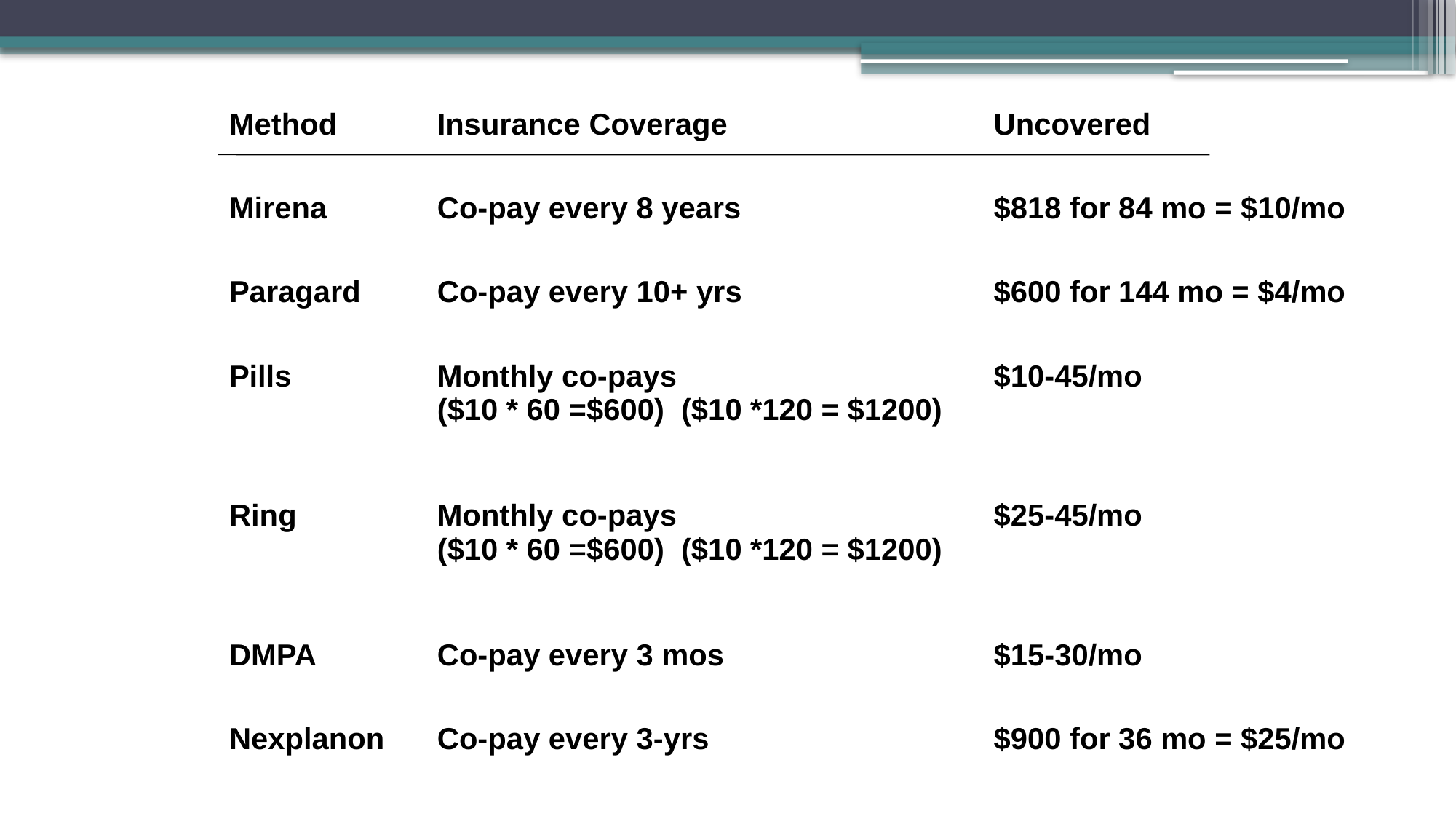

| Method | Insurance Coverage | Uncovered |
| --- | --- | --- |
| Mirena | Co-pay every 8 years | $818 for 84 mo = $10/mo |
| Paragard | Co-pay every 10+ yrs | $600 for 144 mo = $4/mo |
| Pills | Monthly co-pays ($10 \* 60 =$600) ($10 \*120 = $1200) | $10-45/mo |
| Ring | Monthly co-pays ($10 \* 60 =$600) ($10 \*120 = $1200) | $25-45/mo |
| DMPA | Co-pay every 3 mos | $15-30/mo |
| Nexplanon | Co-pay every 3-yrs | $900 for 36 mo = $25/mo |

## Slide 23
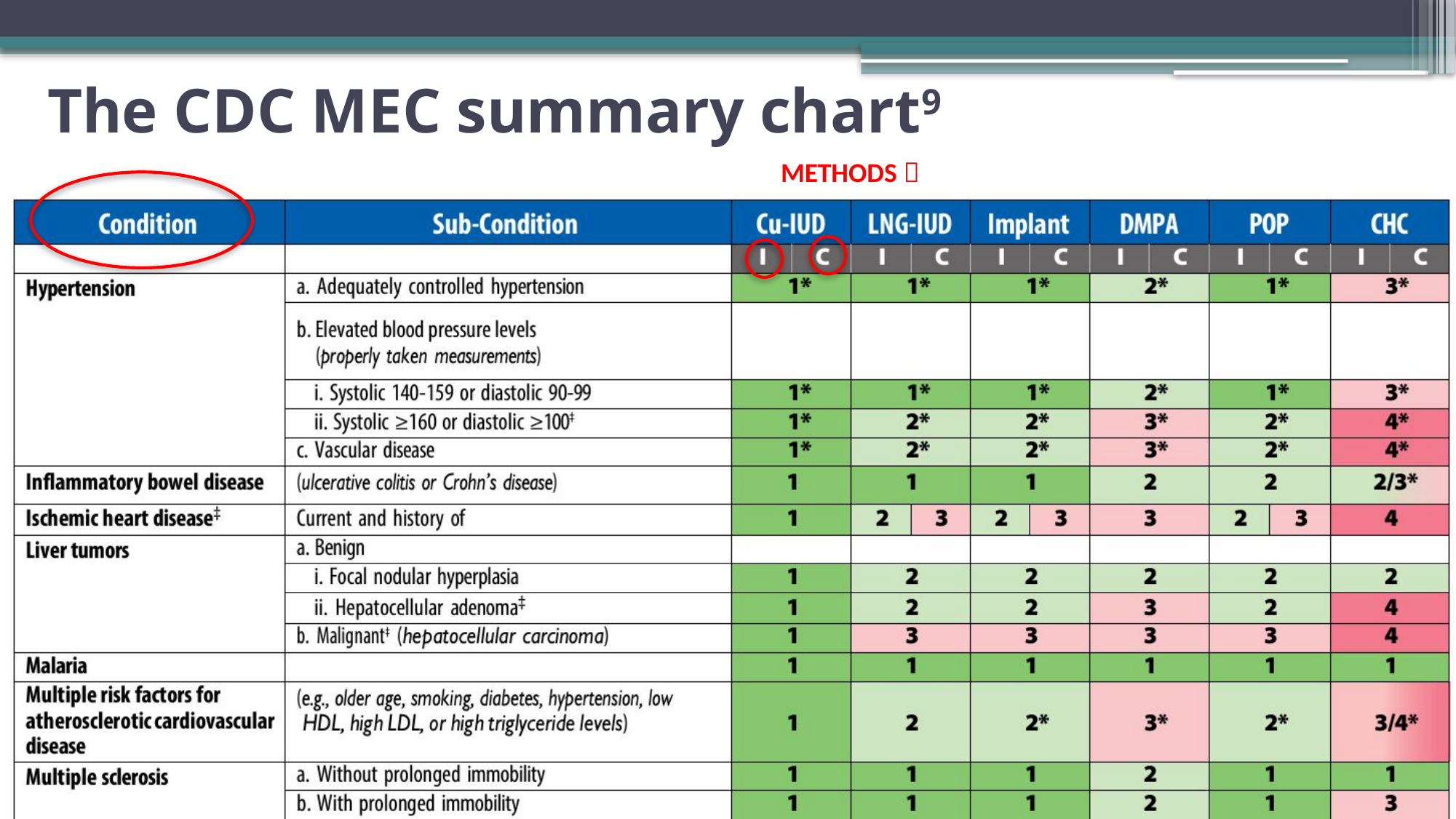

# The CDC MEC summary chart9
METHODS 

## Slide 24
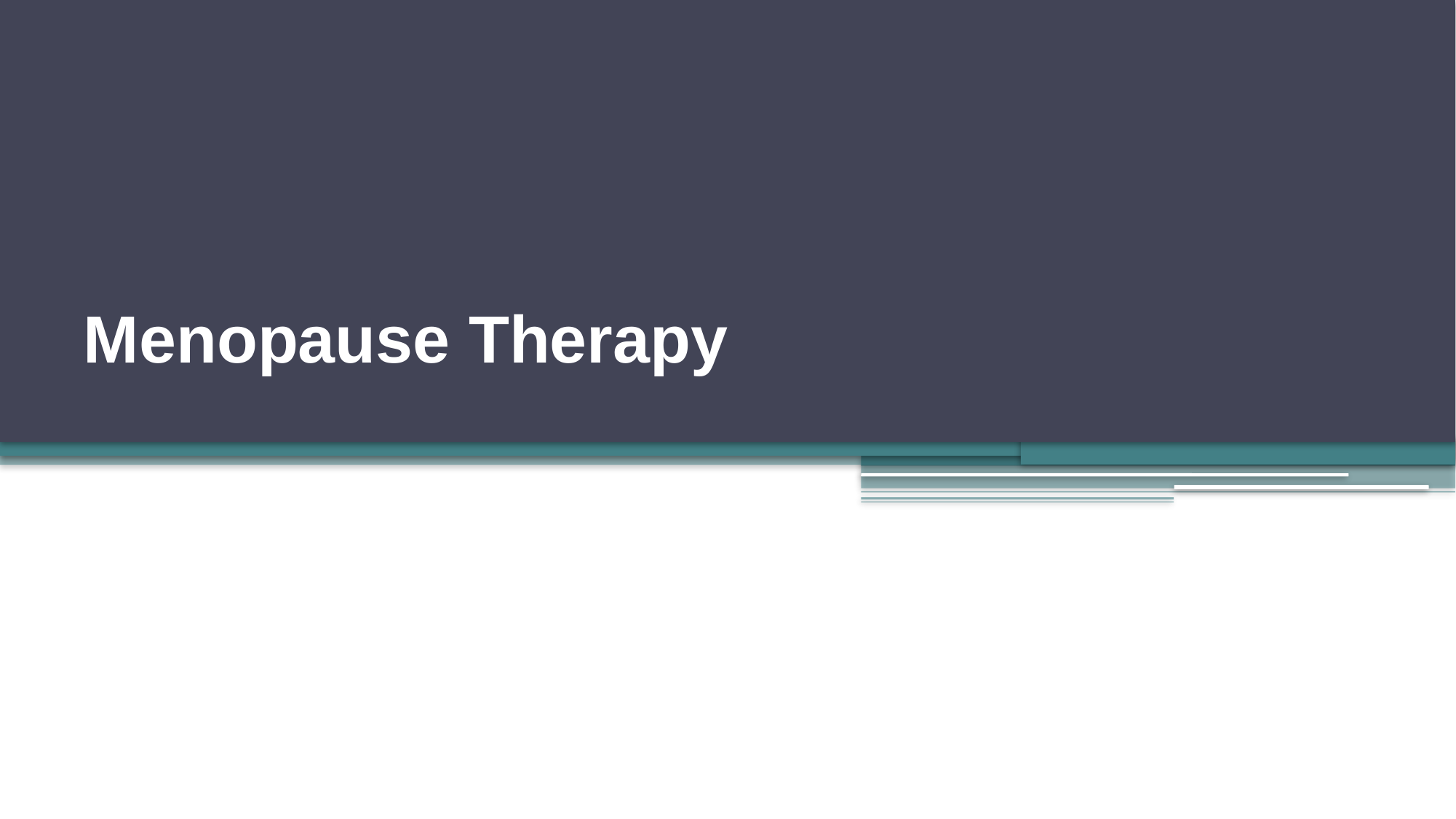

# Menopause Therapy

## Slide 25
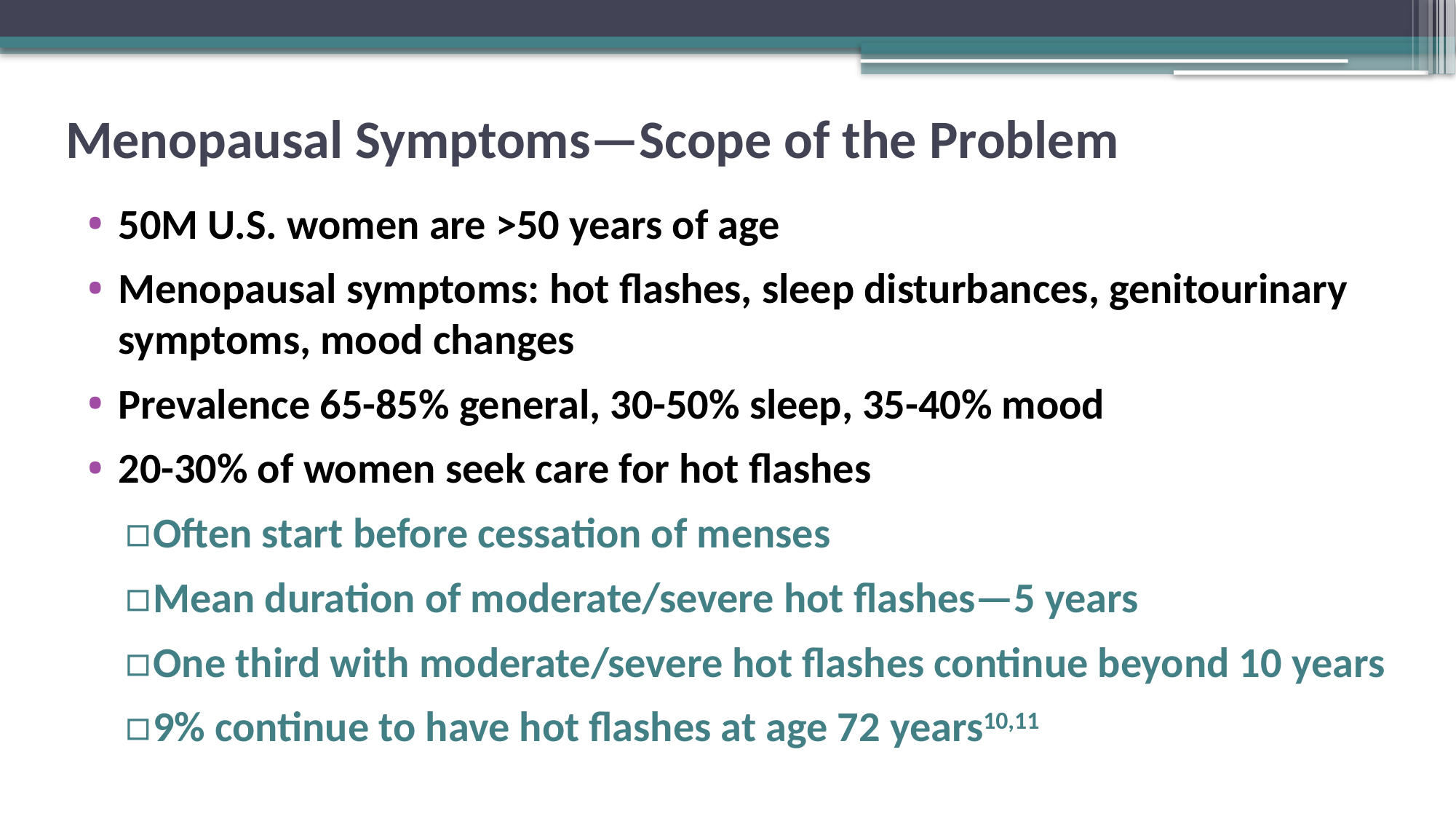

# Menopausal Symptoms—Scope of the Problem
50M U.S. women are >50 years of age
Menopausal symptoms: hot flashes, sleep disturbances, genitourinary symptoms, mood changes
Prevalence 65-85% general, 30-50% sleep, 35-40% mood
20-30% of women seek care for hot flashes
Often start before cessation of menses
Mean duration of moderate/severe hot flashes—5 years
One third with moderate/severe hot flashes continue beyond 10 years
9% continue to have hot flashes at age 72 years10,11

## Slide 26
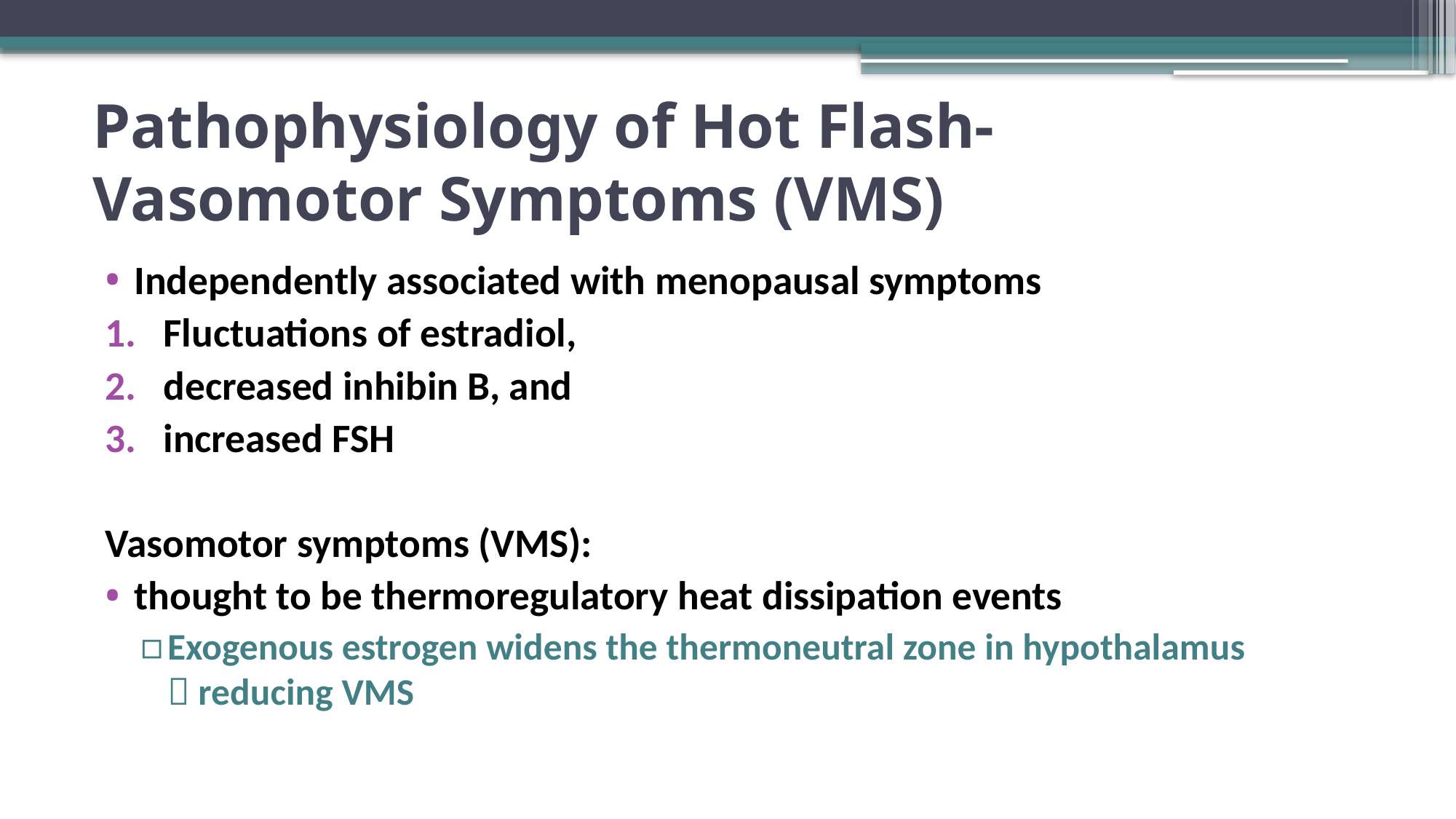

# Pathophysiology of Hot Flash-Vasomotor Symptoms (VMS)
Independently associated with menopausal symptoms
Fluctuations of estradiol,
decreased inhibin B, and
increased FSH
Vasomotor symptoms (VMS):
thought to be thermoregulatory heat dissipation events
Exogenous estrogen widens the thermoneutral zone in hypothalamus  reducing VMS

## Slide 27
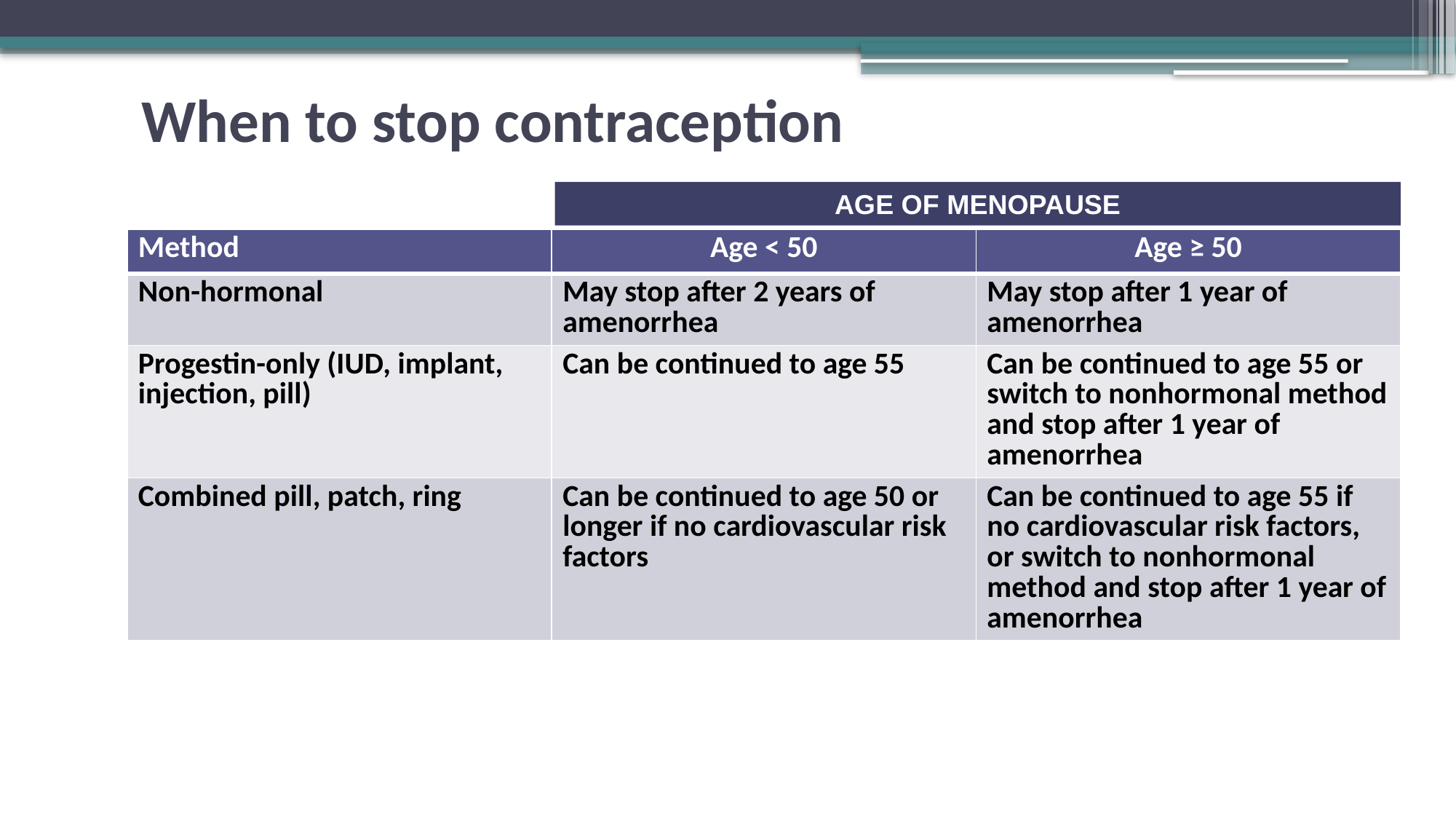

# When to stop contraception
AGE OF MENOPAUSE
| Method | Age < 50 | Age ≥ 50 |
| --- | --- | --- |
| Non-hormonal | May stop after 2 years of amenorrhea | May stop after 1 year of amenorrhea |
| Progestin-only (IUD, implant, injection, pill) | Can be continued to age 55 | Can be continued to age 55 or switch to nonhormonal method and stop after 1 year of amenorrhea |
| Combined pill, patch, ring | Can be continued to age 50 or longer if no cardiovascular risk factors | Can be continued to age 55 if no cardiovascular risk factors, or switch to nonhormonal method and stop after 1 year of amenorrhea |

## Slide 28
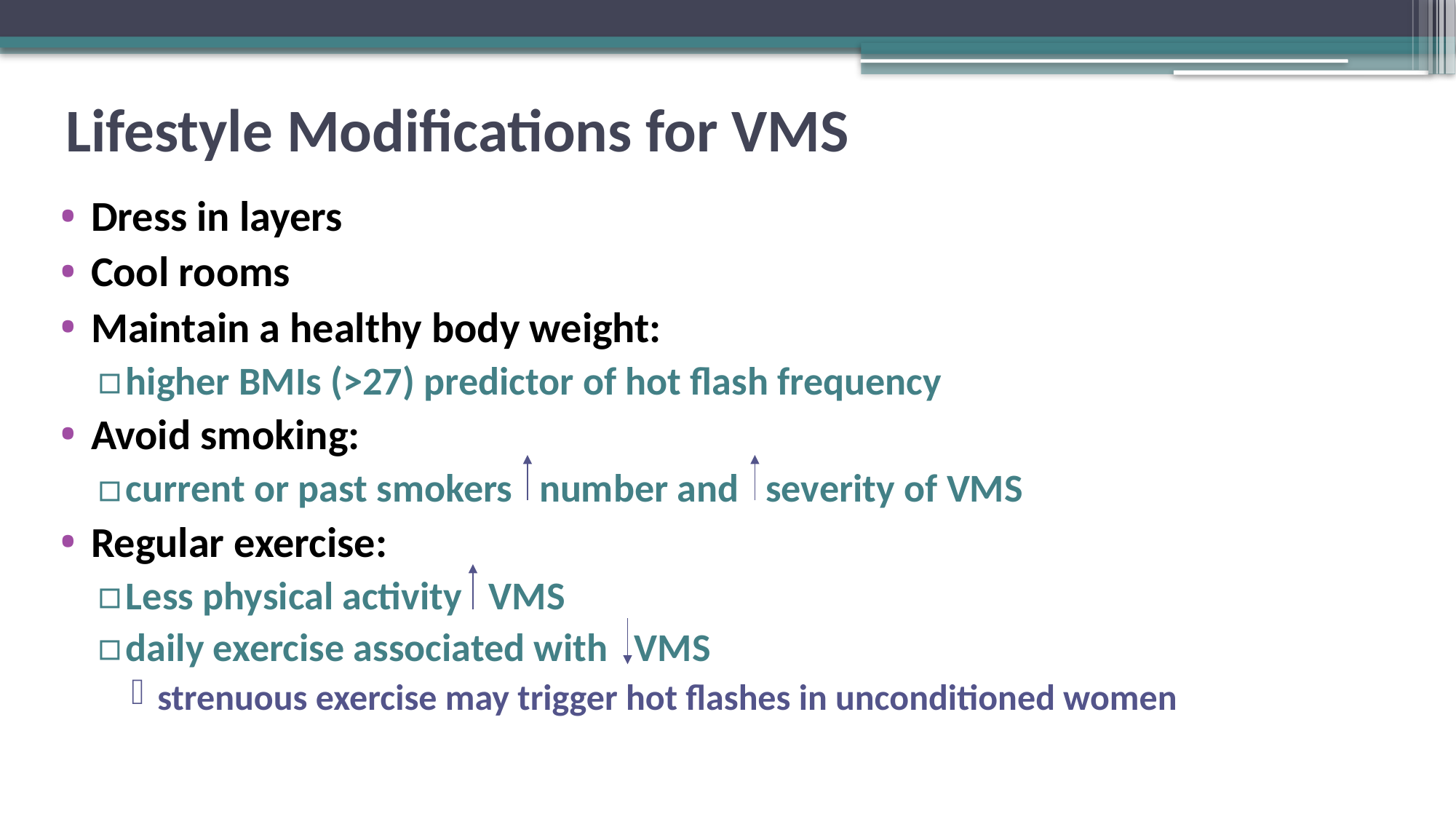

# Lifestyle Modifications for VMS
Dress in layers
Cool rooms
Maintain a healthy body weight:
higher BMIs (>27) predictor of hot flash frequency
Avoid smoking:
current or past smokers number and severity of VMS
Regular exercise:
Less physical activity VMS
daily exercise associated with VMS
strenuous exercise may trigger hot flashes in unconditioned women

## Slide 29
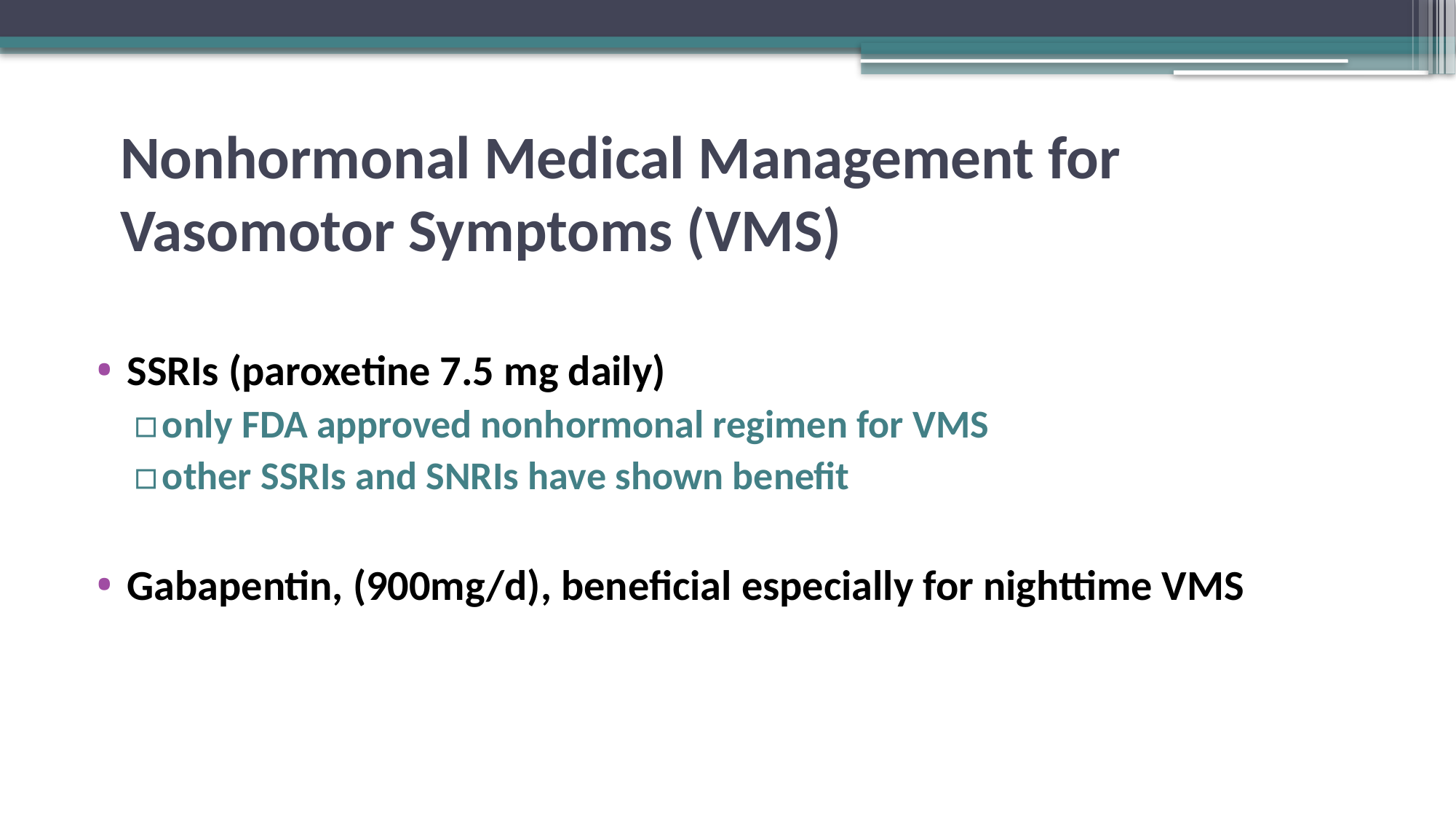

# Nonhormonal Medical Management for Vasomotor Symptoms (VMS)
SSRIs (paroxetine 7.5 mg daily)
only FDA approved nonhormonal regimen for VMS
other SSRIs and SNRIs have shown benefit
Gabapentin, (900mg/d), beneficial especially for nighttime VMS

## Slide 30
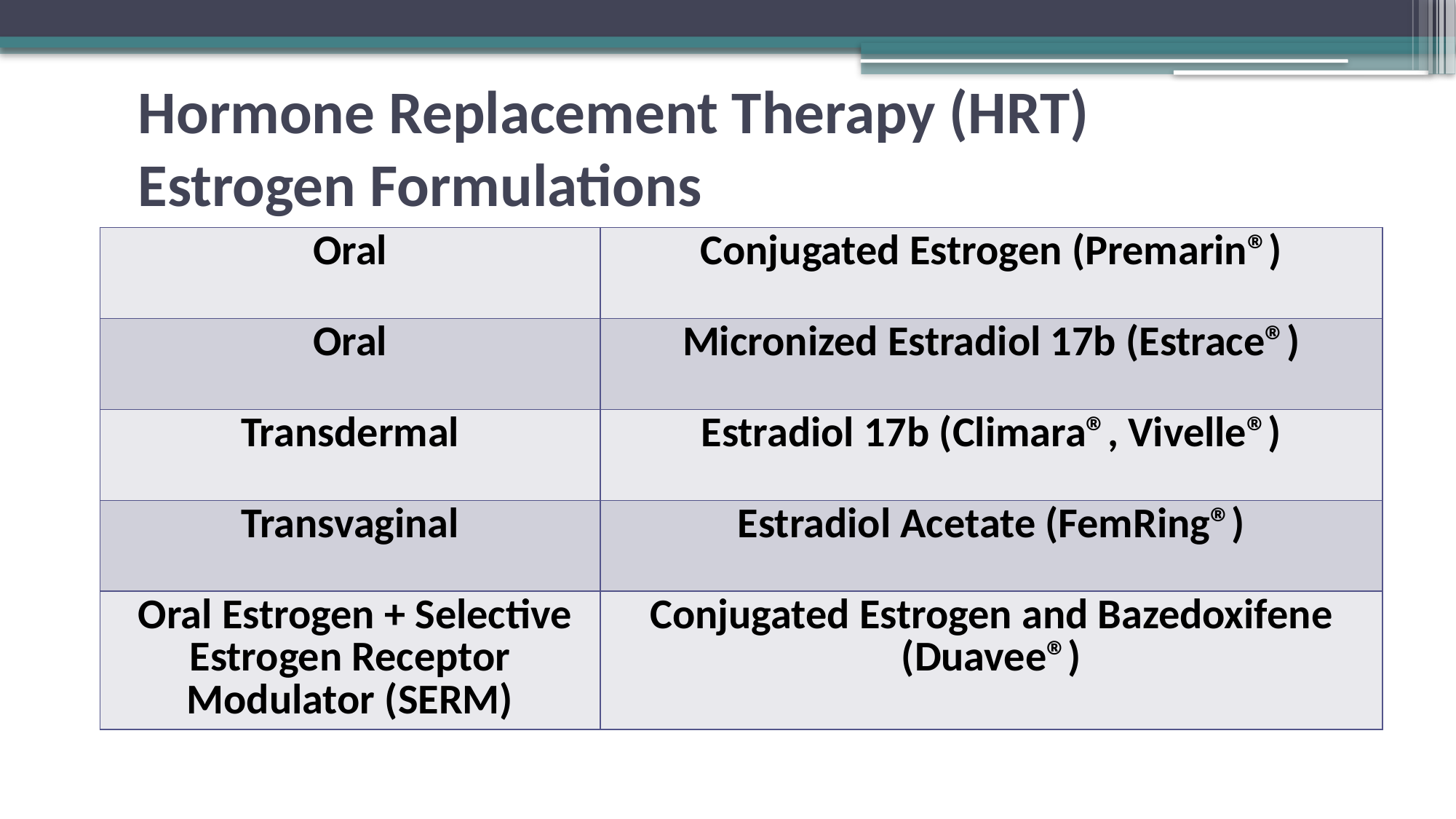

# Hormone Replacement Therapy (HRT) Estrogen Formulations
| Oral | Conjugated Estrogen (Premarin®) |
| --- | --- |
| Oral | Micronized Estradiol 17b (Estrace®) |
| Transdermal | Estradiol 17b (Climara®, Vivelle®) |
| Transvaginal | Estradiol Acetate (FemRing®) |
| Oral Estrogen + Selective Estrogen Receptor Modulator (SERM) | Conjugated Estrogen and Bazedoxifene (Duavee®) |

## Slide 31
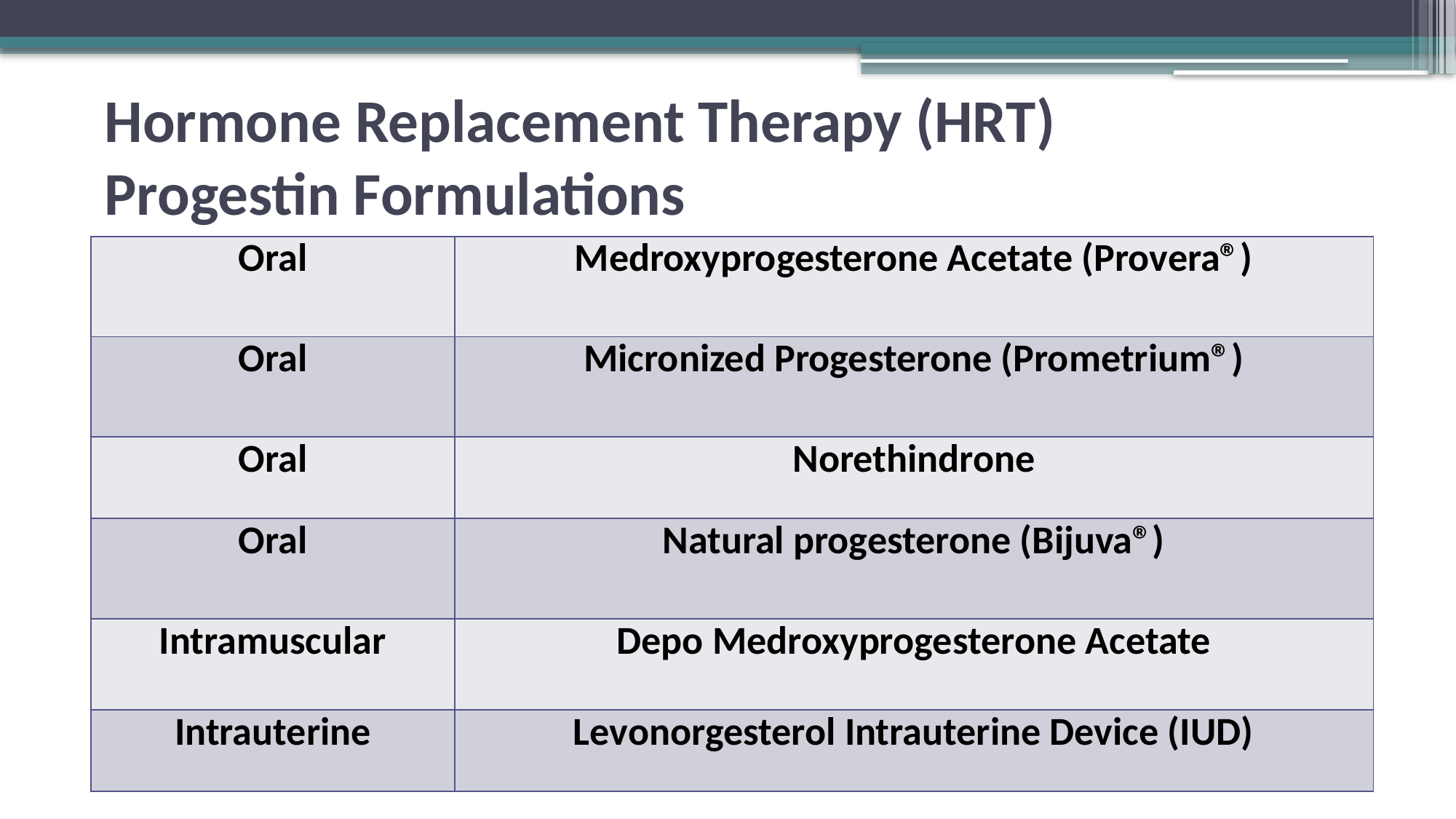

# Hormone Replacement Therapy (HRT) Progestin Formulations
| Oral | Medroxyprogesterone Acetate (Provera®) |
| --- | --- |
| Oral | Micronized Progesterone (Prometrium®) |
| Oral | Norethindrone |
| Oral | Natural progesterone (Bijuva®) |
| Intramuscular | Depo Medroxyprogesterone Acetate |
| Intrauterine | Levonorgesterol Intrauterine Device (IUD) |

## Slide 32
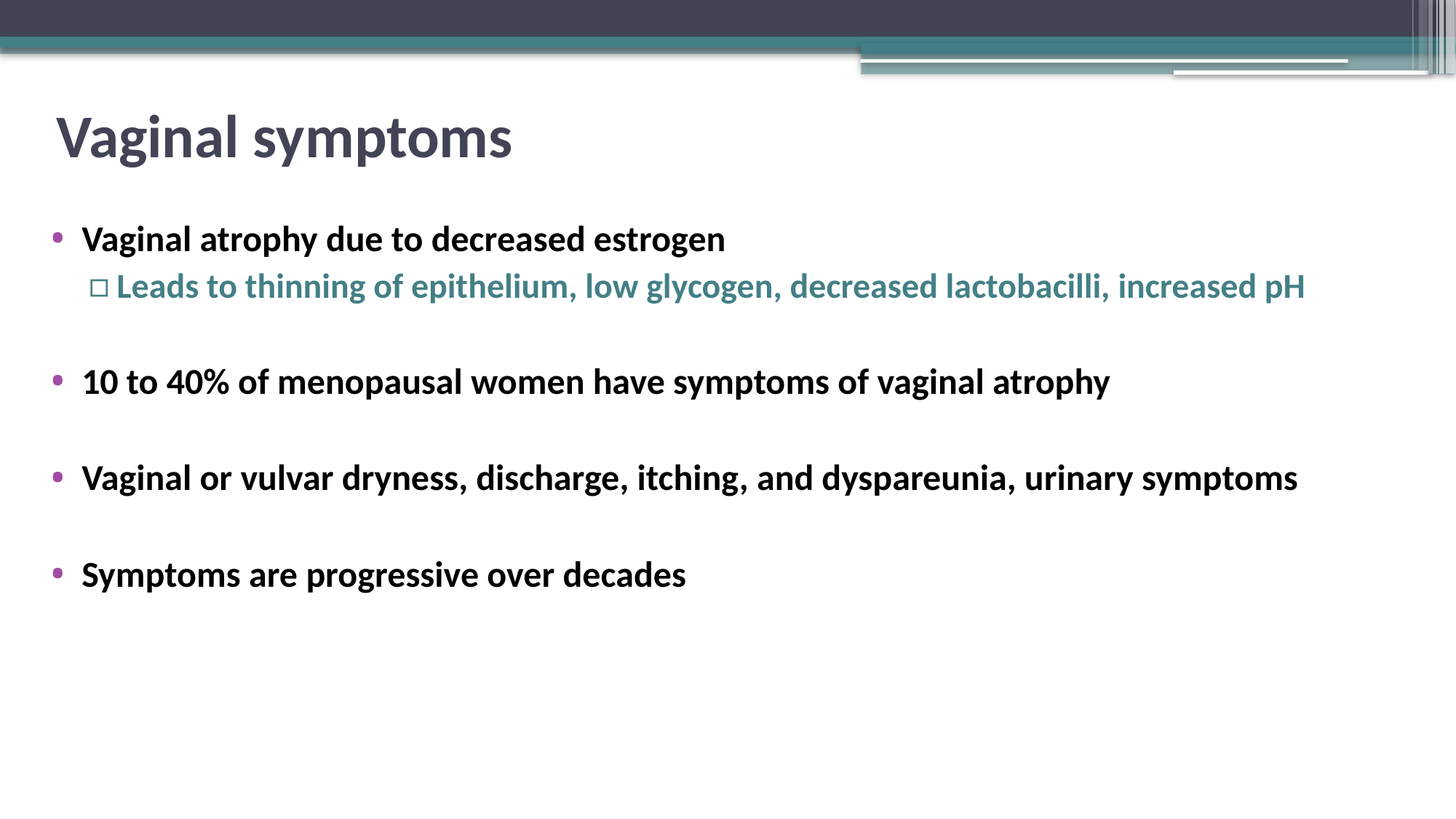

# Vaginal symptoms
Vaginal atrophy due to decreased estrogen
Leads to thinning of epithelium, low glycogen, decreased lactobacilli, increased pH
10 to 40% of menopausal women have symptoms of vaginal atrophy
Vaginal or vulvar dryness, discharge, itching, and dyspareunia, urinary symptoms
Symptoms are progressive over decades

## Slide 33
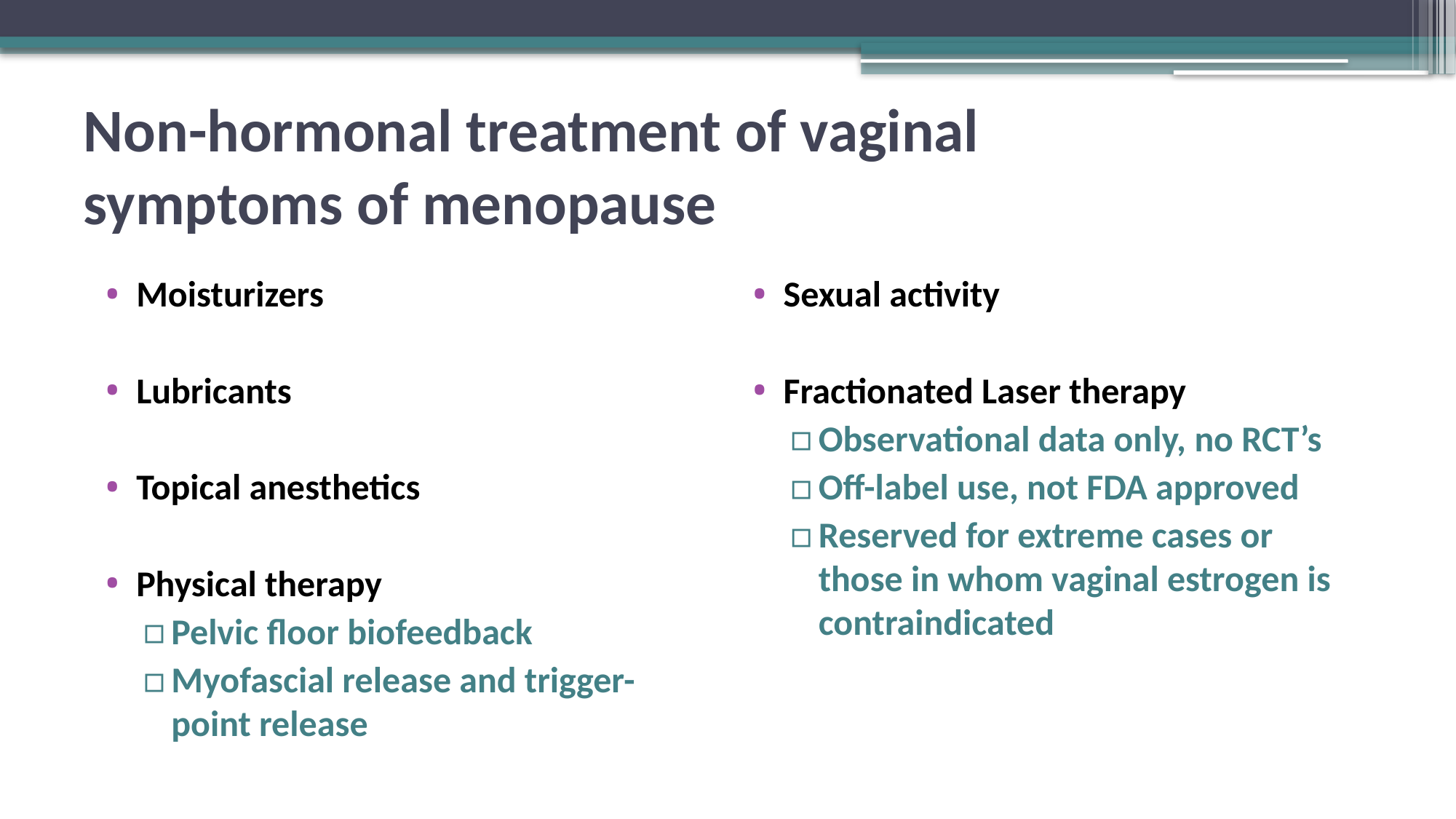

# Non-hormonal treatment of vaginal symptoms of menopause
Moisturizers
Lubricants
Topical anesthetics
Physical therapy
Pelvic floor biofeedback
Myofascial release and trigger-point release
Sexual activity
Fractionated Laser therapy
Observational data only, no RCT’s
Off-label use, not FDA approved
Reserved for extreme cases or those in whom vaginal estrogen is contraindicated

## Slide 34
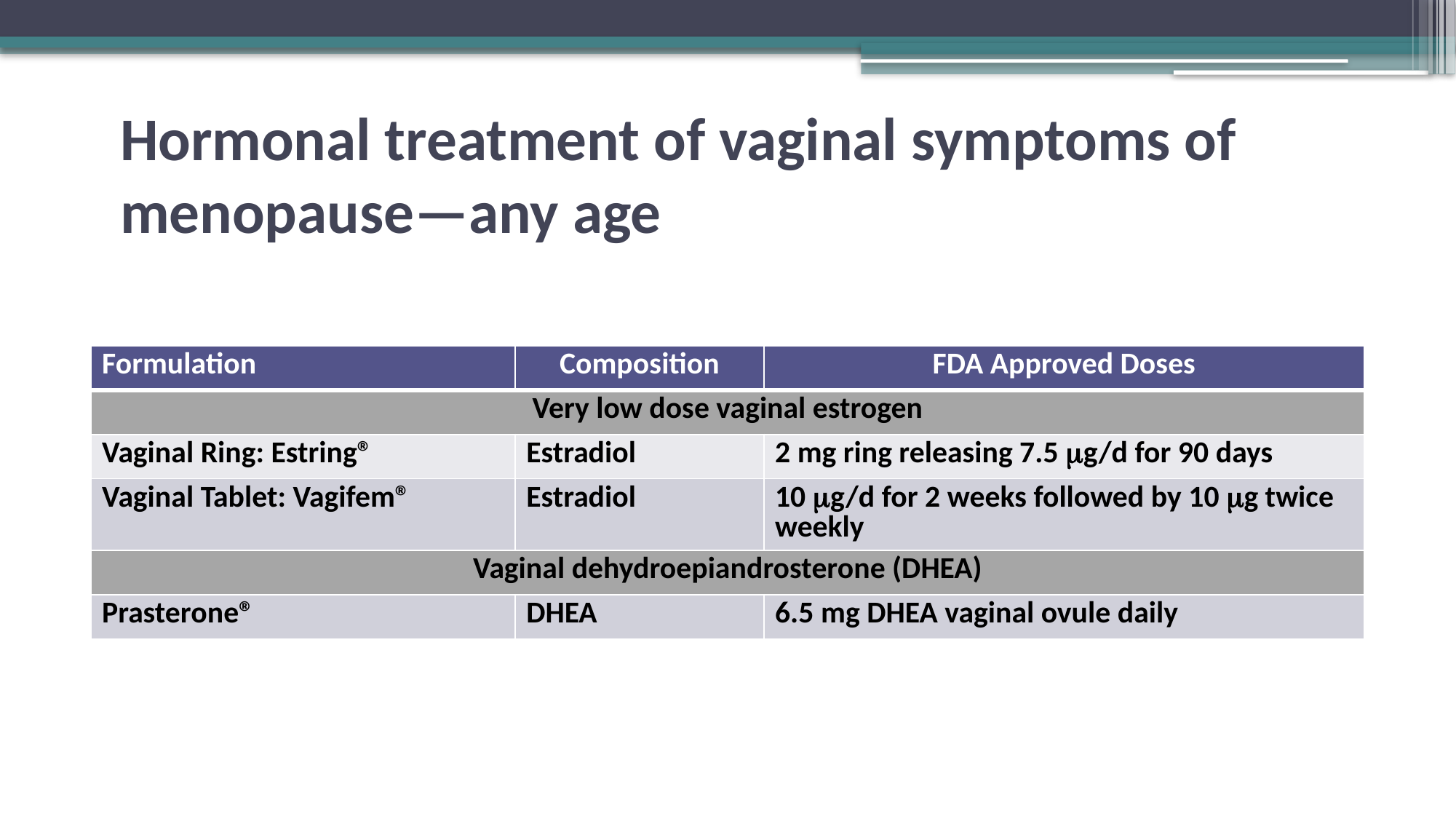

# Hormonal treatment of vaginal symptoms of menopause—any age
| Formulation | Composition | FDA Approved Doses |
| --- | --- | --- |
| Very low dose vaginal estrogen | | |
| Vaginal Ring: Estring® | Estradiol | 2 mg ring releasing 7.5 mg/d for 90 days |
| Vaginal Tablet: Vagifem® | Estradiol | 10 mg/d for 2 weeks followed by 10 mg twice weekly |
| Vaginal dehydroepiandrosterone (DHEA) | | |
| Prasterone® | DHEA | 6.5 mg DHEA vaginal ovule daily |

## Slide 35
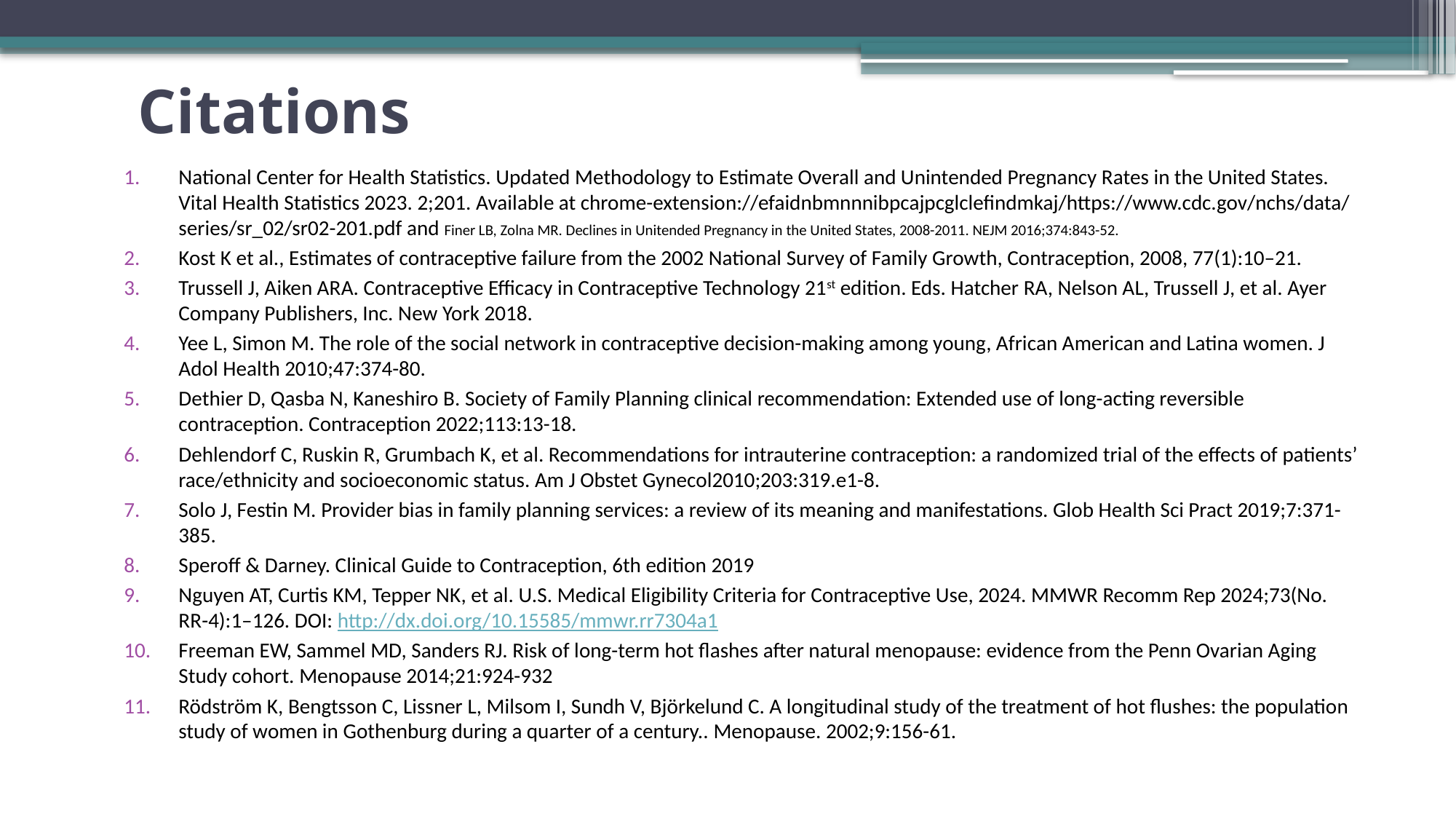

# Citations
National Center for Health Statistics. Updated Methodology to Estimate Overall and Unintended Pregnancy Rates in the United States. Vital Health Statistics 2023. 2;201. Available at chrome-extension://efaidnbmnnnibpcajpcglclefindmkaj/https://www.cdc.gov/nchs/data/series/sr_02/sr02-201.pdf and Finer LB, Zolna MR. Declines in Unitended Pregnancy in the United States, 2008-2011. NEJM 2016;374:843-52.
Kost K et al., Estimates of contraceptive failure from the 2002 National Survey of Family Growth, Contraception, 2008, 77(1):10–21.
Trussell J, Aiken ARA. Contraceptive Efficacy in Contraceptive Technology 21st edition. Eds. Hatcher RA, Nelson AL, Trussell J, et al. Ayer Company Publishers, Inc. New York 2018.
Yee L, Simon M. The role of the social network in contraceptive decision-making among young, African American and Latina women. J Adol Health 2010;47:374-80.
Dethier D, Qasba N, Kaneshiro B. Society of Family Planning clinical recommendation: Extended use of long-acting reversible contraception. Contraception 2022;113:13-18.
Dehlendorf C, Ruskin R, Grumbach K, et al. Recommendations for intrauterine contraception: a randomized trial of the effects of patients’ race/ethnicity and socioeconomic status. Am J Obstet Gynecol2010;203:319.e1-8.
Solo J, Festin M. Provider bias in family planning services: a review of its meaning and manifestations. Glob Health Sci Pract 2019;7:371-385.
Speroff & Darney. Clinical Guide to Contraception, 6th edition 2019
Nguyen AT, Curtis KM, Tepper NK, et al. U.S. Medical Eligibility Criteria for Contraceptive Use, 2024. MMWR Recomm Rep 2024;73(No. RR-4):1–126. DOI: http://dx.doi.org/10.15585/mmwr.rr7304a1
Freeman EW, Sammel MD, Sanders RJ. Risk of long-term hot flashes after natural menopause: evidence from the Penn Ovarian Aging Study cohort. Menopause 2014;21:924-932
Rödström K, Bengtsson C, Lissner L, Milsom I, Sundh V, Björkelund C. A longitudinal study of the treatment of hot flushes: the population study of women in Gothenburg during a quarter of a century.. Menopause. 2002;9:156-61.
