# Supplementary material for: Benefits of Hormonal Contraception Across the Lifespan: A Case-Based, Interactive Curriculum
Source: MedEdPORTAL. 2025 Apr 4;21:11512. doi: 10.15766/mep_2374-8265.11512 (PMC11968450; doi:10.15766/mep_2374-8265.11512)
Supplement: Supplementary file 1 — Student Guide and Case 1.docxCase 2.docxCase 3.docxCDC Eligibility Criteria for Contraceptive Use.pdfBN How Well Does Birth Control Work.pdfRHAP Birth Control Across the Gender Spectrum.pdfCounseling for the Hormones Found in Contraceptives.pptxCase-Based Collaborative Learning.pptxFaculty Guide.docxLongitudinal Assessment Questions.docx [file mep_2374-8265.11512-s001.zip › H. Case-Based Collaborative Learning.pptx]

## Slide 1
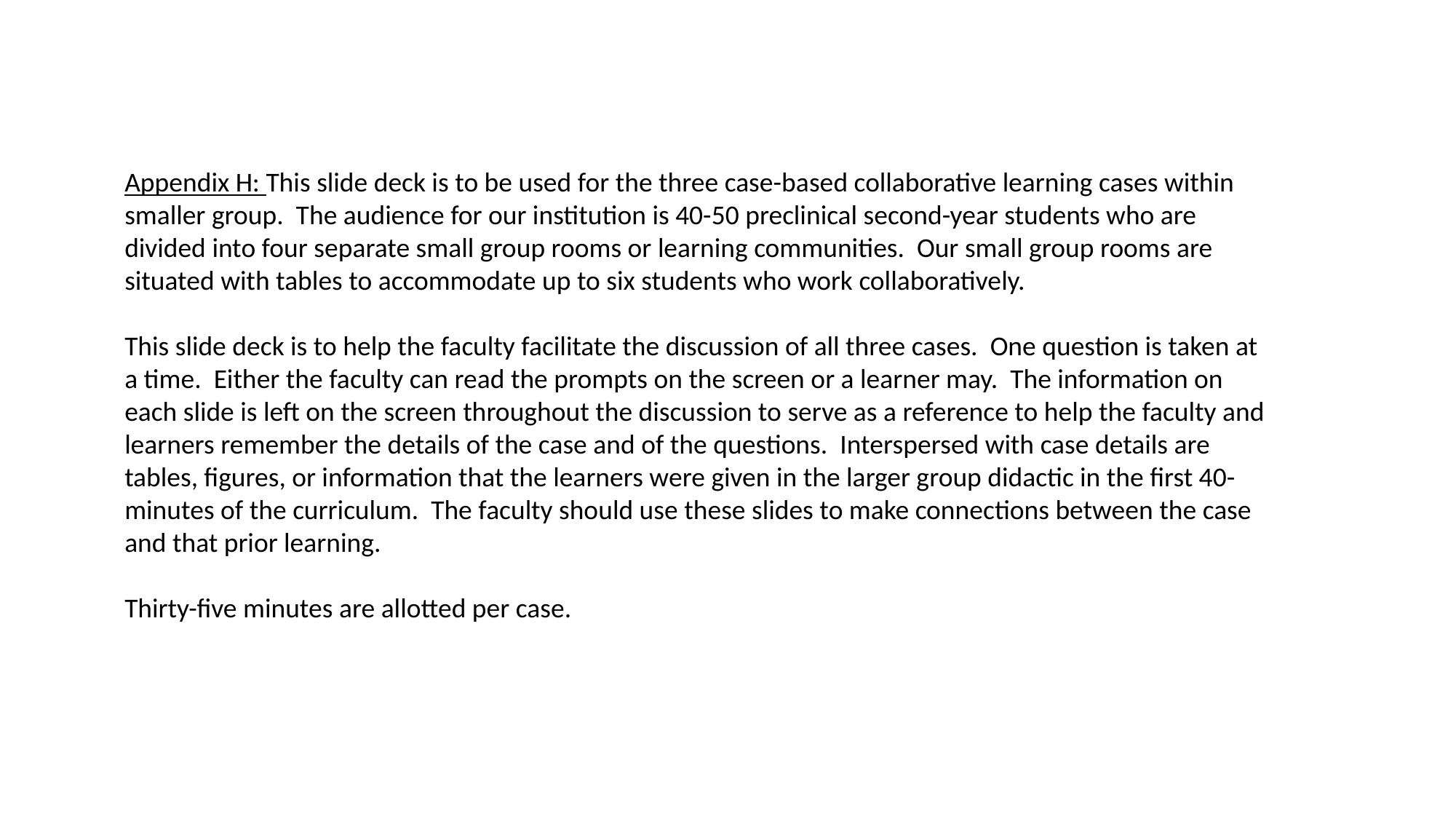

Appendix H: This slide deck is to be used for the three case-based collaborative learning cases within smaller group. The audience for our institution is 40-50 preclinical second-year students who are divided into four separate small group rooms or learning communities. Our small group rooms are situated with tables to accommodate up to six students who work collaboratively.
This slide deck is to help the faculty facilitate the discussion of all three cases. One question is taken at a time. Either the faculty can read the prompts on the screen or a learner may. The information on each slide is left on the screen throughout the discussion to serve as a reference to help the faculty and learners remember the details of the case and of the questions. Interspersed with case details are tables, figures, or information that the learners were given in the larger group didactic in the first 40-minutes of the curriculum. The faculty should use these slides to make connections between the case and that prior learning.
Thirty-five minutes are allotted per case.

## Slide 2
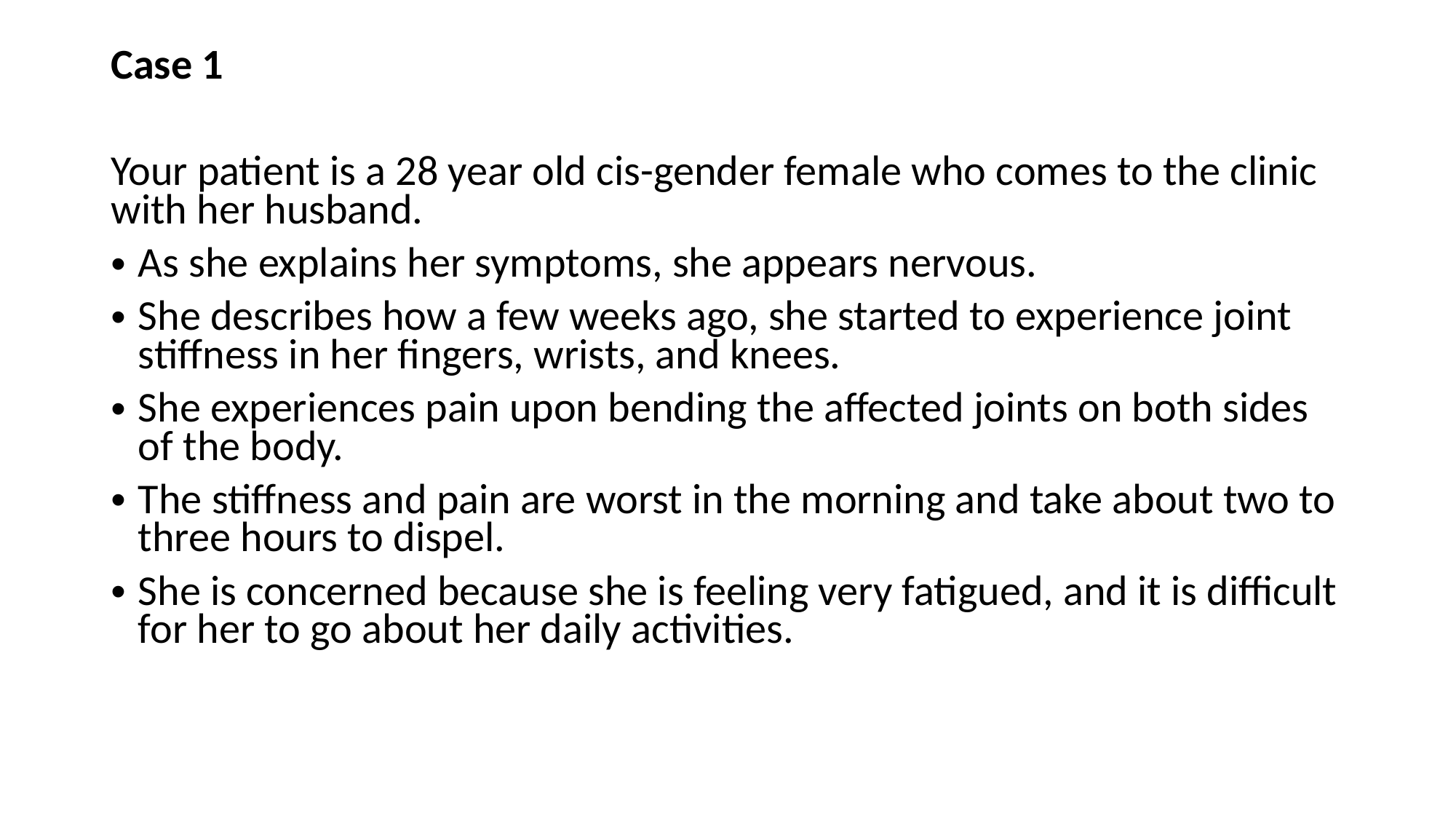

Case 1
Your patient is a 28 year old cis-gender female who comes to the clinic with her husband.
As she explains her symptoms, she appears nervous.
She describes how a few weeks ago, she started to experience joint stiffness in her fingers, wrists, and knees.
She experiences pain upon bending the affected joints on both sides of the body.
The stiffness and pain are worst in the morning and take about two to three hours to dispel.
She is concerned because she is feeling very fatigued, and it is difficult for her to go about her daily activities.

## Slide 3
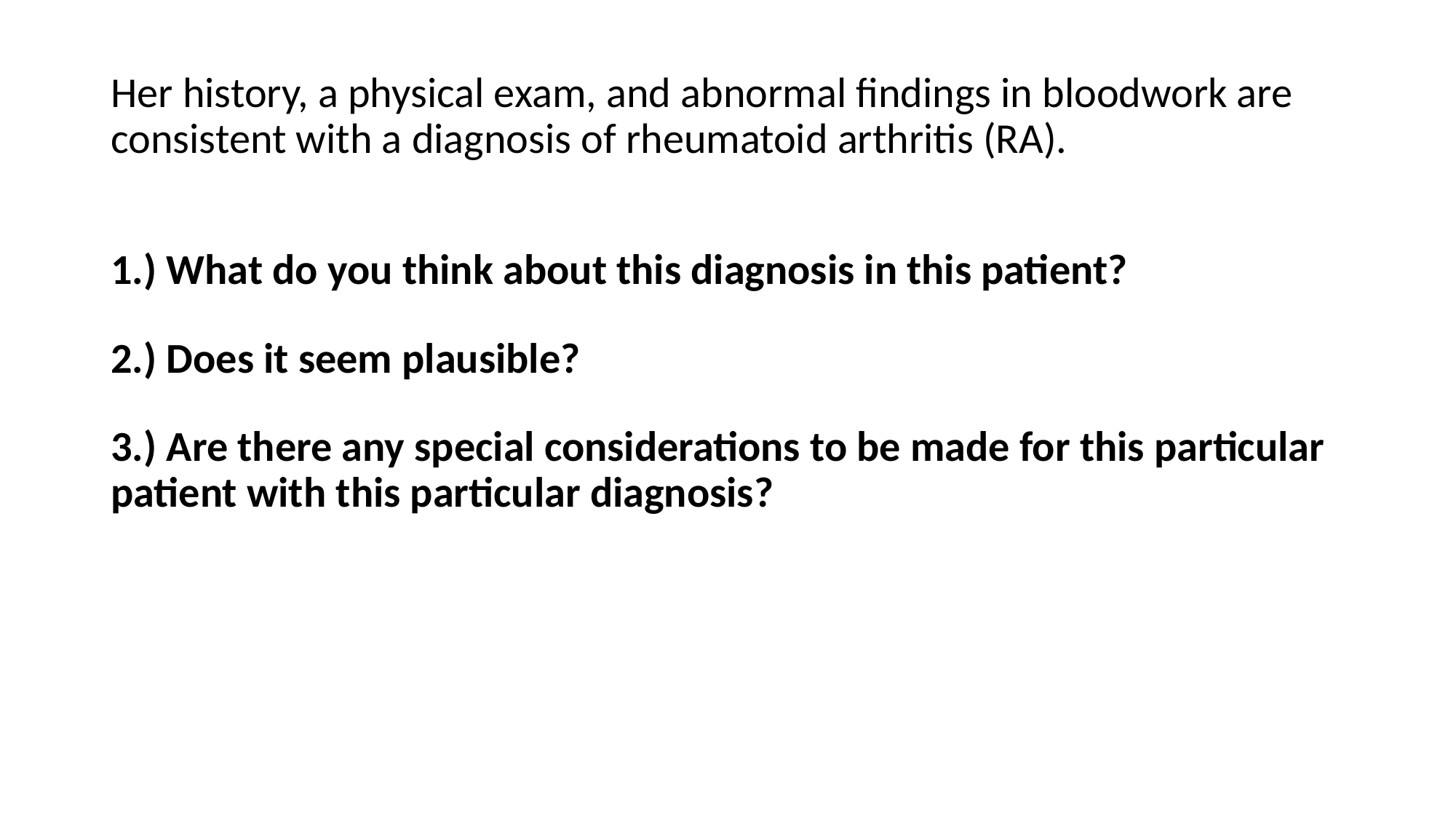

Her history, a physical exam, and abnormal findings in bloodwork are consistent with a diagnosis of rheumatoid arthritis (RA).
1.) What do you think about this diagnosis in this patient?
2.) Does it seem plausible?
3.) Are there any special considerations to be made for this particular patient with this particular diagnosis?

## Slide 4
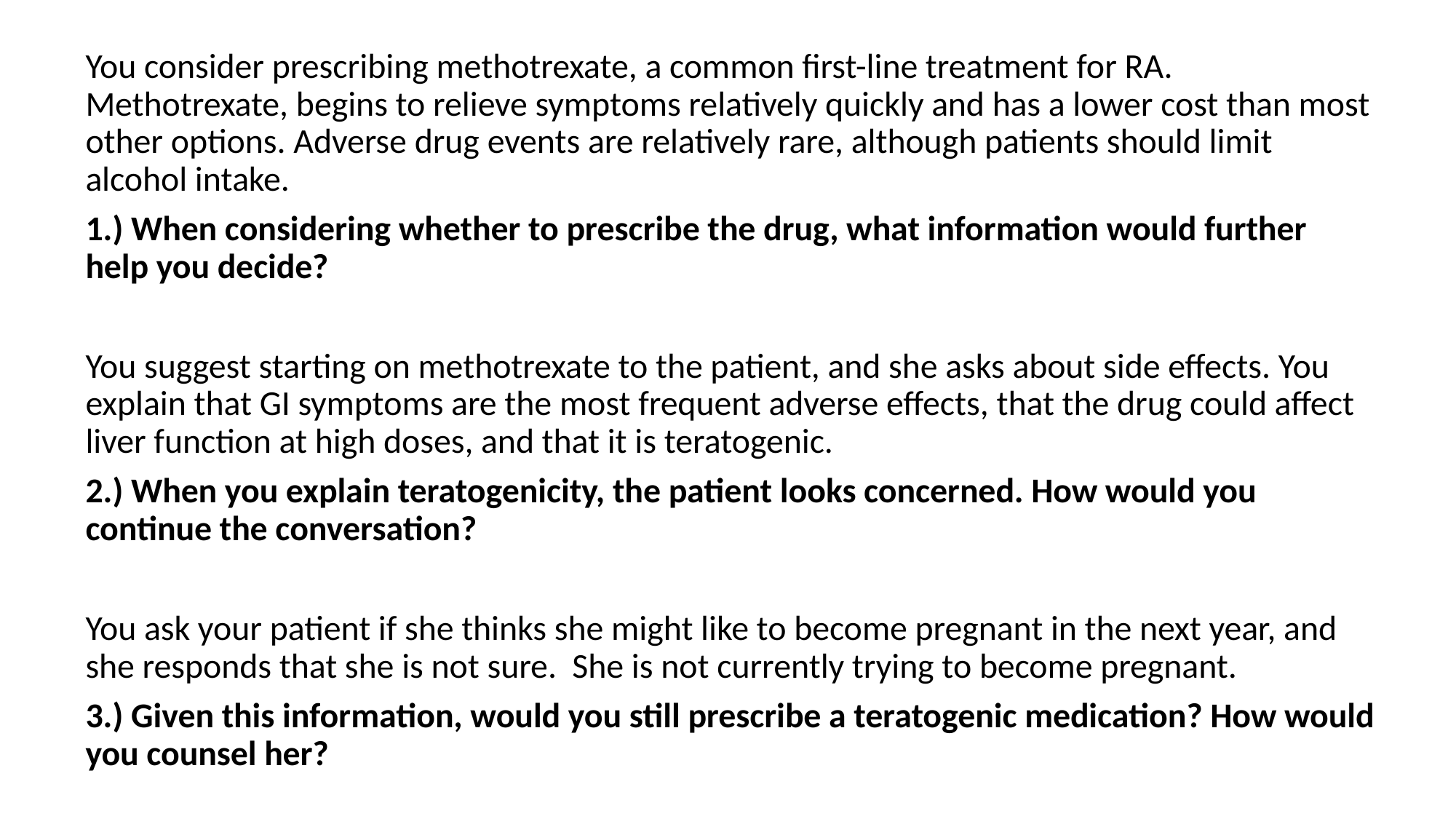

You consider prescribing methotrexate, a common first-line treatment for RA. Methotrexate, begins to relieve symptoms relatively quickly and has a lower cost than most other options. Adverse drug events are relatively rare, although patients should limit alcohol intake.
1.) When considering whether to prescribe the drug, what information would further help you decide?
You suggest starting on methotrexate to the patient, and she asks about side effects. You explain that GI symptoms are the most frequent adverse effects, that the drug could affect liver function at high doses, and that it is teratogenic.
2.) When you explain teratogenicity, the patient looks concerned. How would you continue the conversation?
You ask your patient if she thinks she might like to become pregnant in the next year, and she responds that she is not sure. She is not currently trying to become pregnant.
3.) Given this information, would you still prescribe a teratogenic medication? How would you counsel her?

## Slide 5
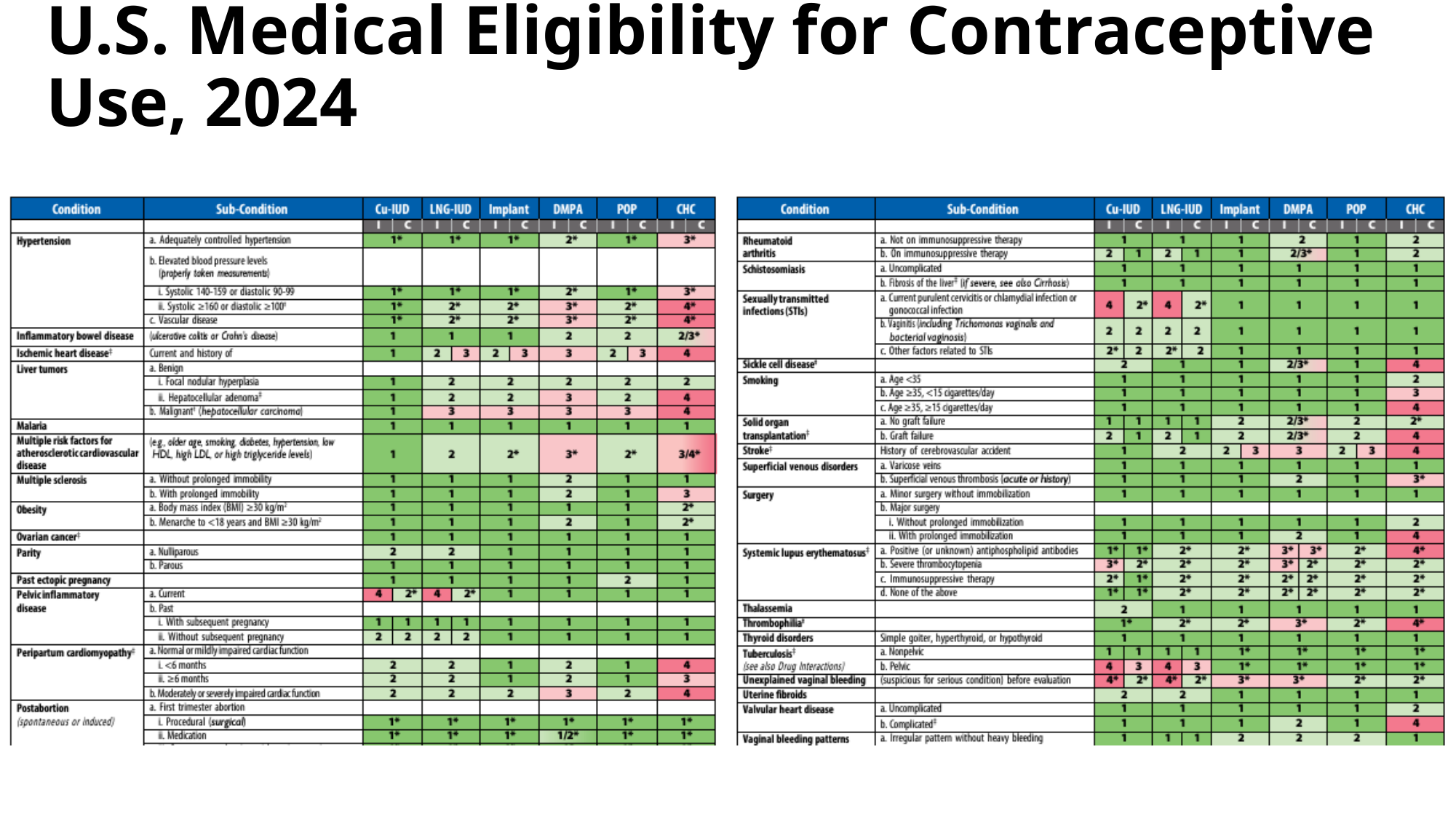

# U.S. Medical Eligibility for Contraceptive Use, 2024

## Slide 6
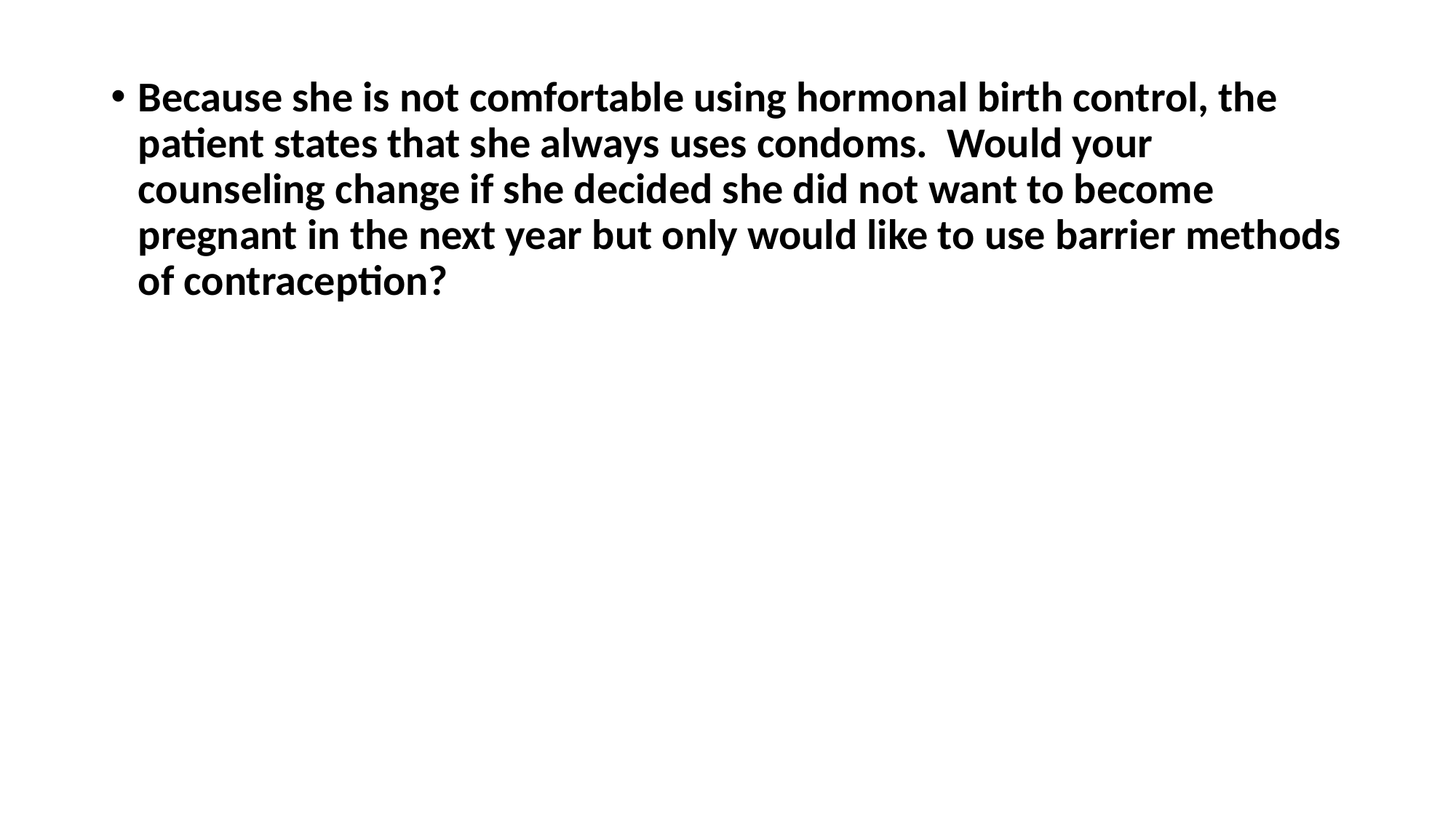

Because she is not comfortable using hormonal birth control, the patient states that she always uses condoms. Would your counseling change if she decided she did not want to become pregnant in the next year but only would like to use barrier methods of contraception?

## Slide 7
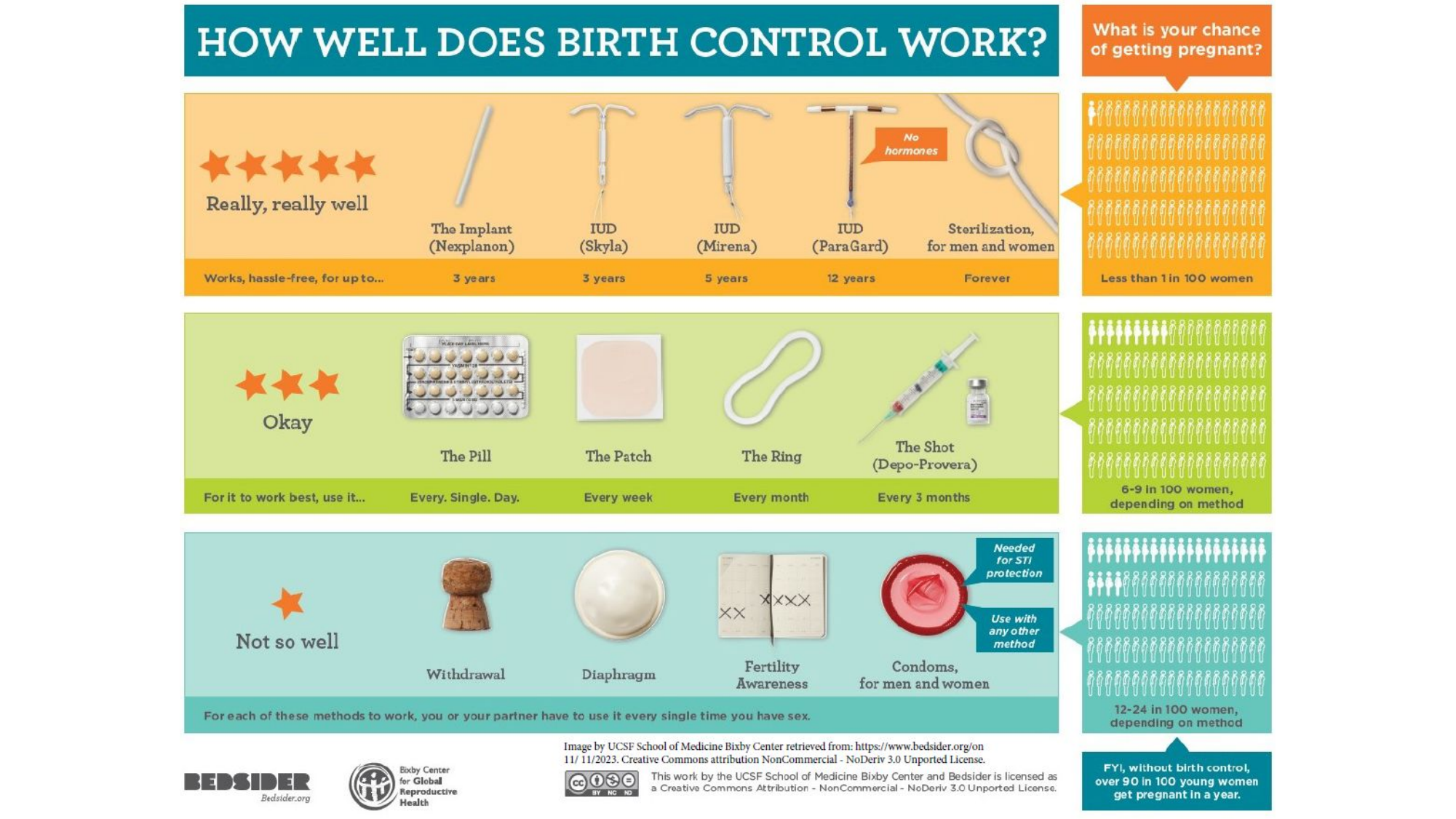

## Slide 8
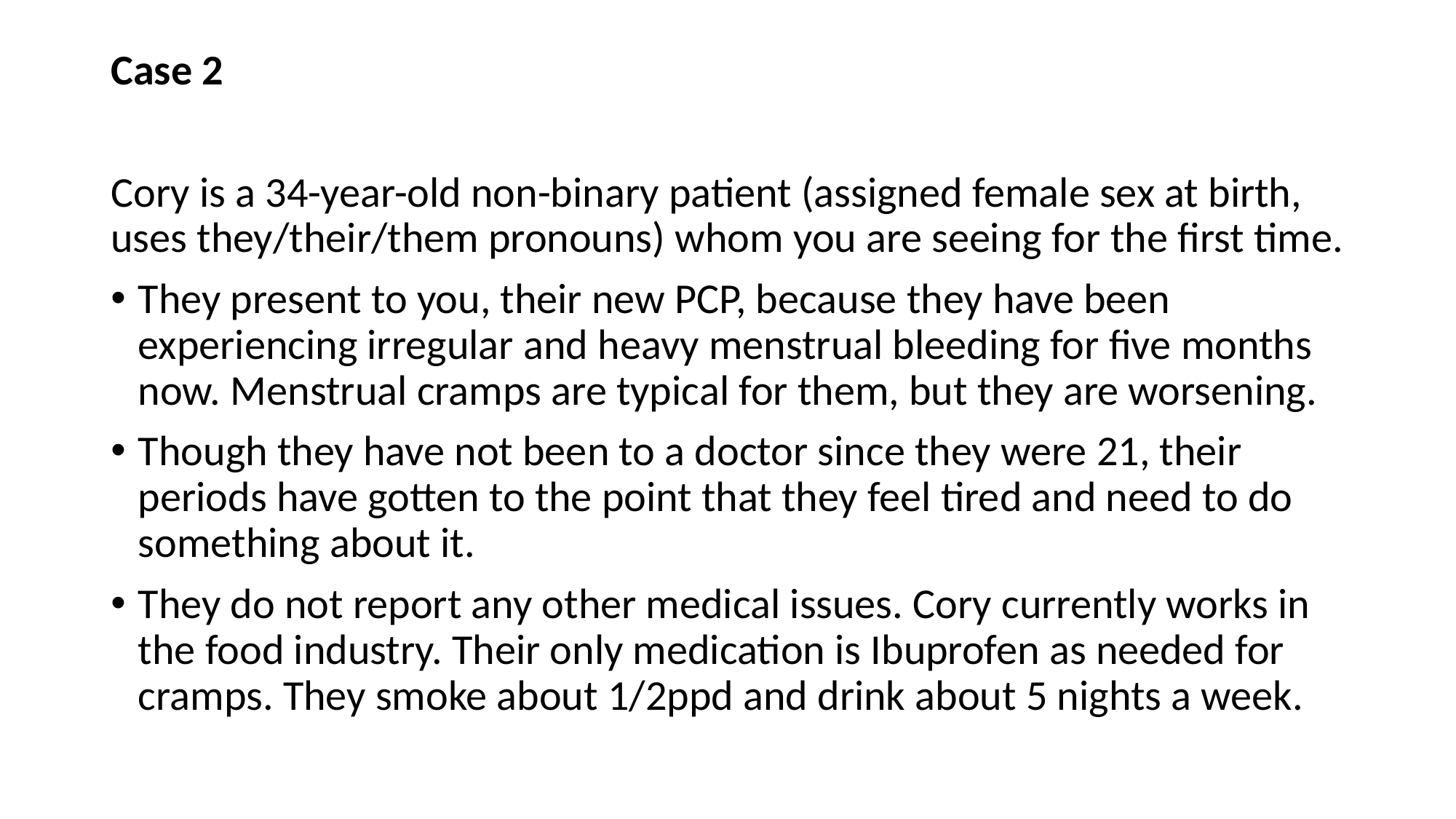

Case 2
Cory is a 34-year-old non-binary patient (assigned female sex at birth, uses they/their/them pronouns) whom you are seeing for the first time.
They present to you, their new PCP, because they have been experiencing irregular and heavy menstrual bleeding for five months now. Menstrual cramps are typical for them, but they are worsening.
Though they have not been to a doctor since they were 21, their periods have gotten to the point that they feel tired and need to do something about it.
They do not report any other medical issues. Cory currently works in the food industry. Their only medication is Ibuprofen as needed for cramps. They smoke about 1/2ppd and drink about 5 nights a week.

## Slide 9
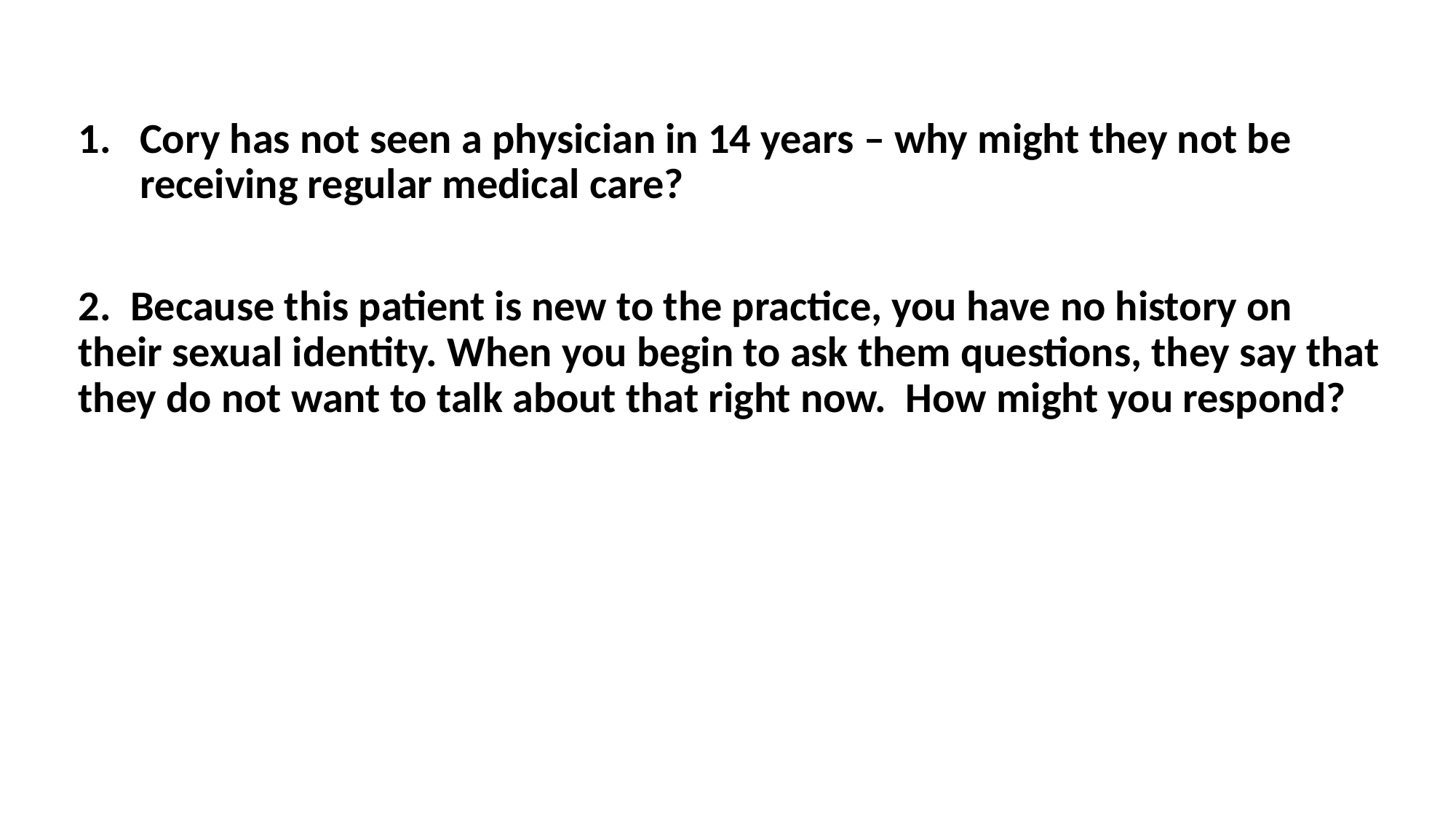

Cory has not seen a physician in 14 years – why might they not be receiving regular medical care?
2. Because this patient is new to the practice, you have no history on their sexual identity. When you begin to ask them questions, they say that they do not want to talk about that right now. How might you respond?

## Slide 10
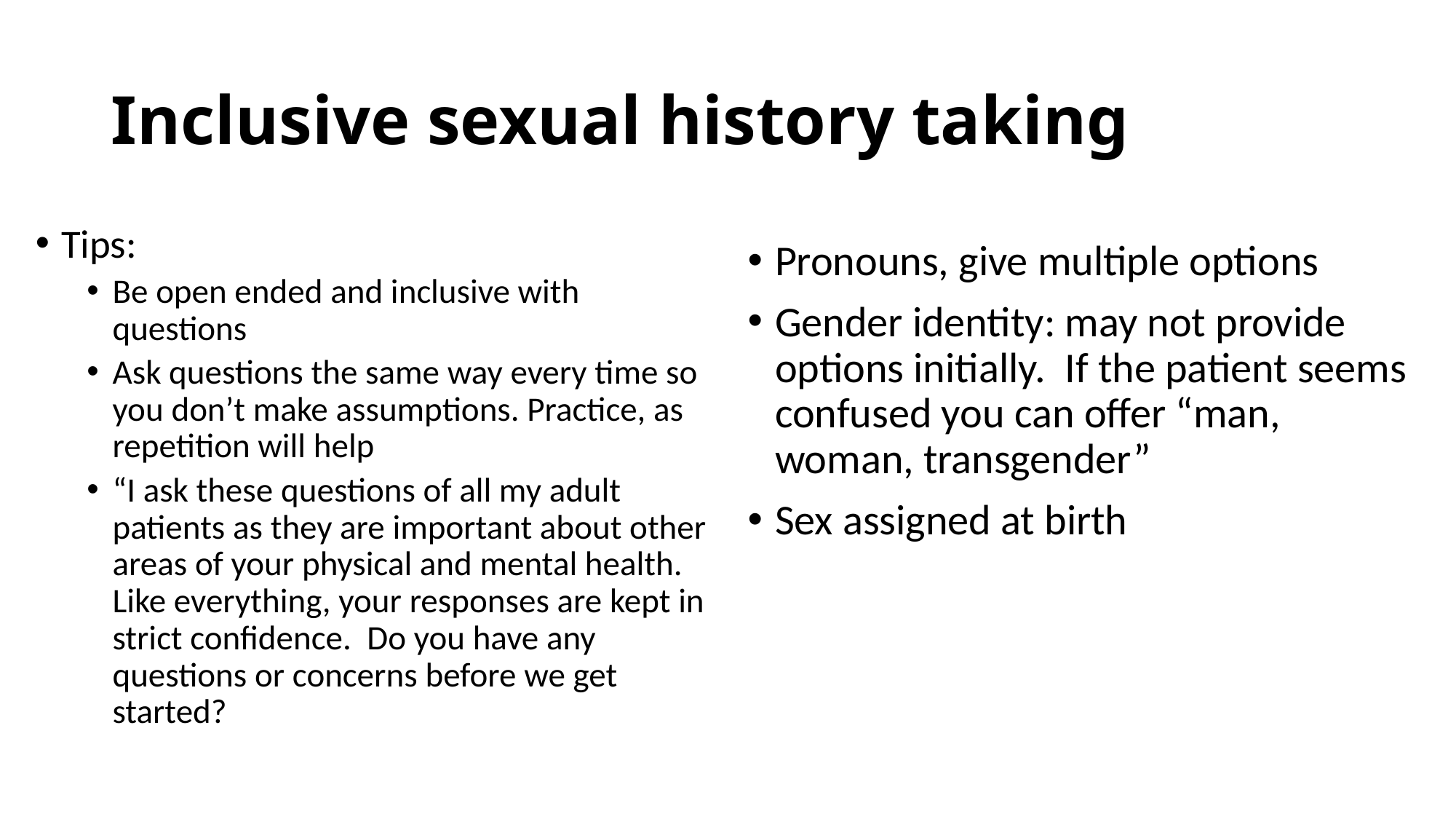

# Inclusive sexual history taking
Tips:
Be open ended and inclusive with questions
Ask questions the same way every time so you don’t make assumptions. Practice, as repetition will help
“I ask these questions of all my adult patients as they are important about other areas of your physical and mental health. Like everything, your responses are kept in strict confidence. Do you have any questions or concerns before we get started?
Pronouns, give multiple options
Gender identity: may not provide options initially. If the patient seems confused you can offer “man, woman, transgender”
Sex assigned at birth

## Slide 11
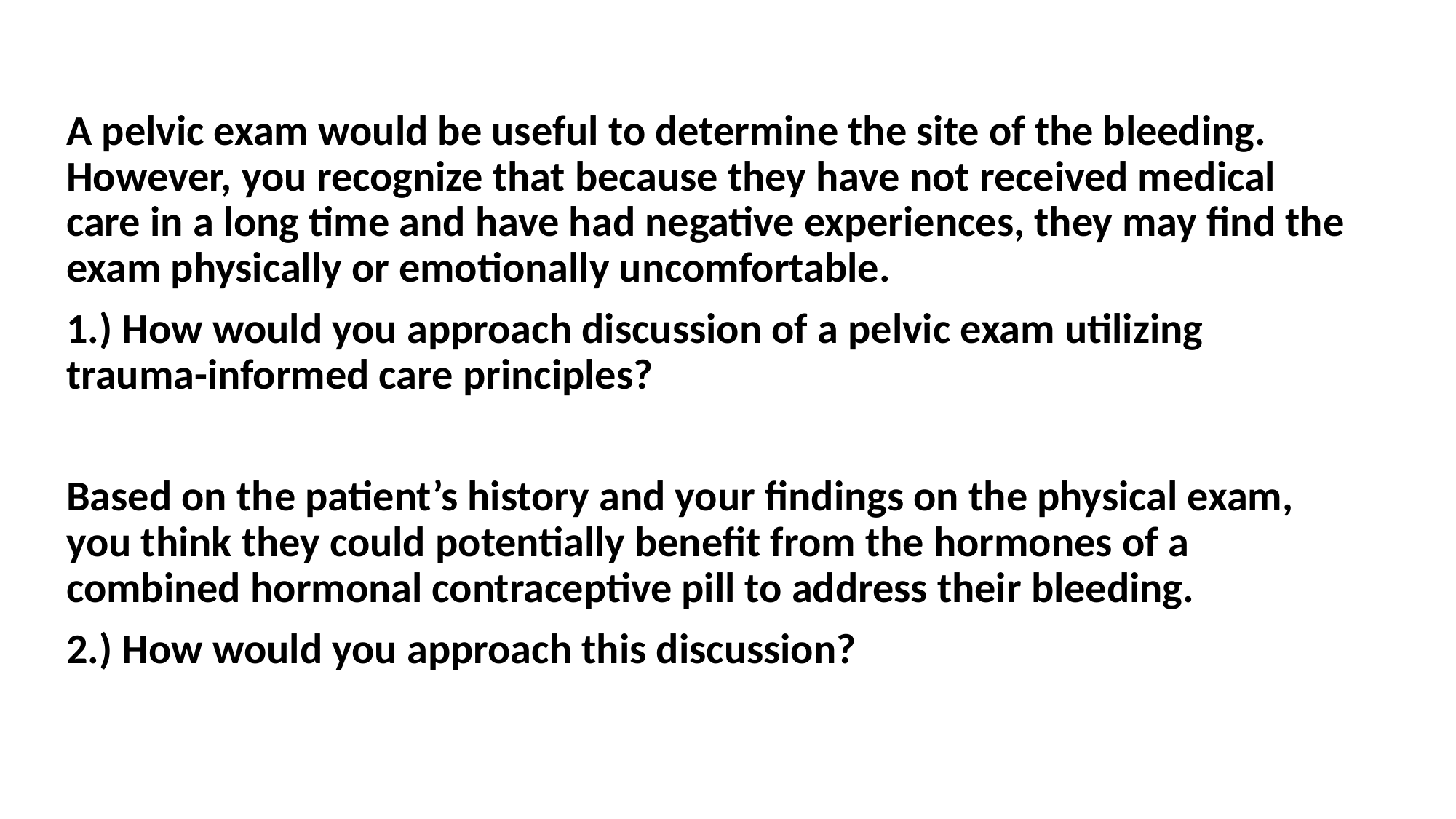

A pelvic exam would be useful to determine the site of the bleeding. However, you recognize that because they have not received medical care in a long time and have had negative experiences, they may find the exam physically or emotionally uncomfortable.
1.) How would you approach discussion of a pelvic exam utilizing trauma-informed care principles?
Based on the patient’s history and your findings on the physical exam, you think they could potentially benefit from the hormones of a combined hormonal contraceptive pill to address their bleeding.
2.) How would you approach this discussion?

## Slide 12
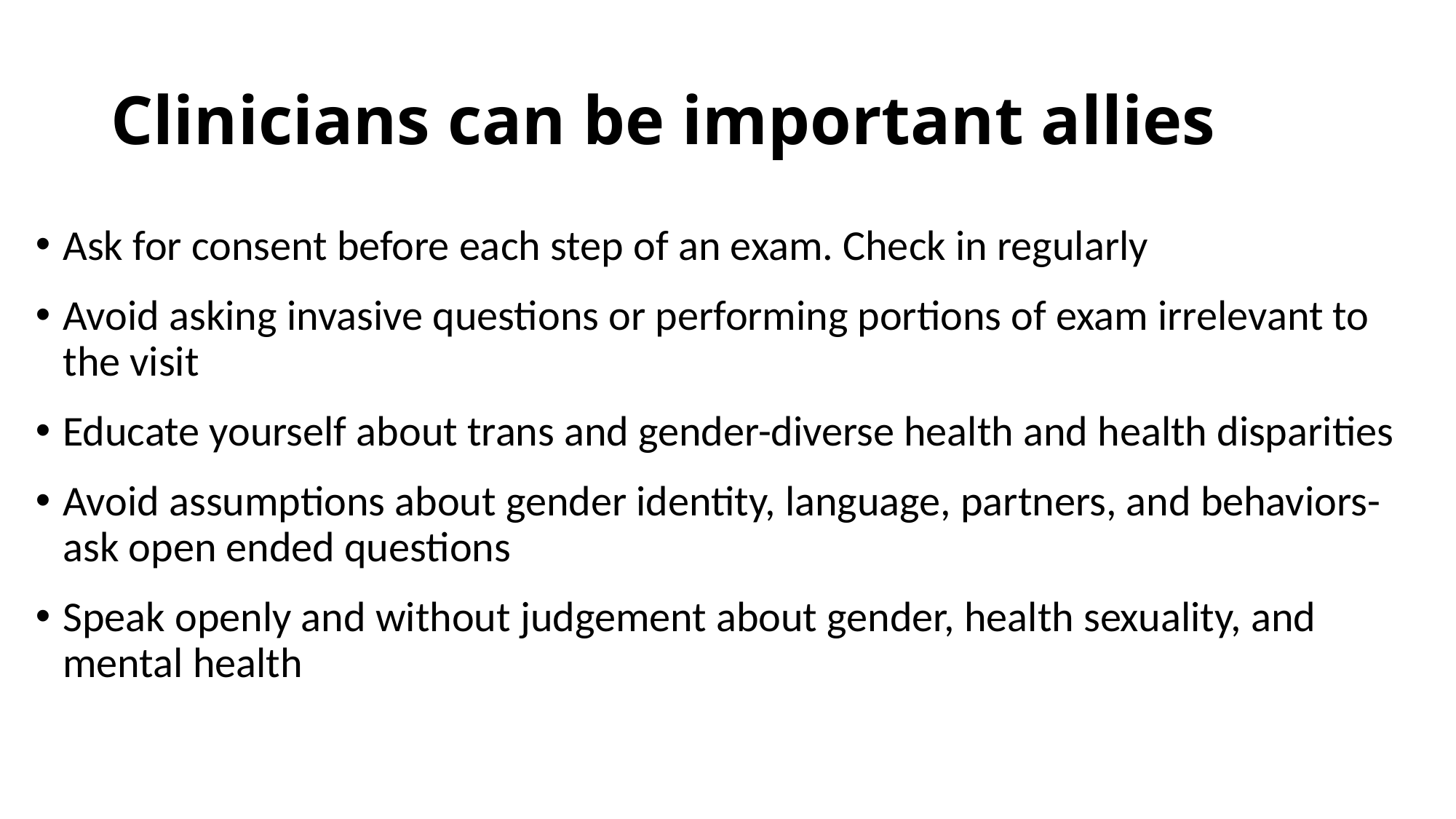

# Clinicians can be important allies
Ask for consent before each step of an exam. Check in regularly
Avoid asking invasive questions or performing portions of exam irrelevant to the visit
Educate yourself about trans and gender-diverse health and health disparities
Avoid assumptions about gender identity, language, partners, and behaviors-ask open ended questions
Speak openly and without judgement about gender, health sexuality, and mental health

## Slide 13
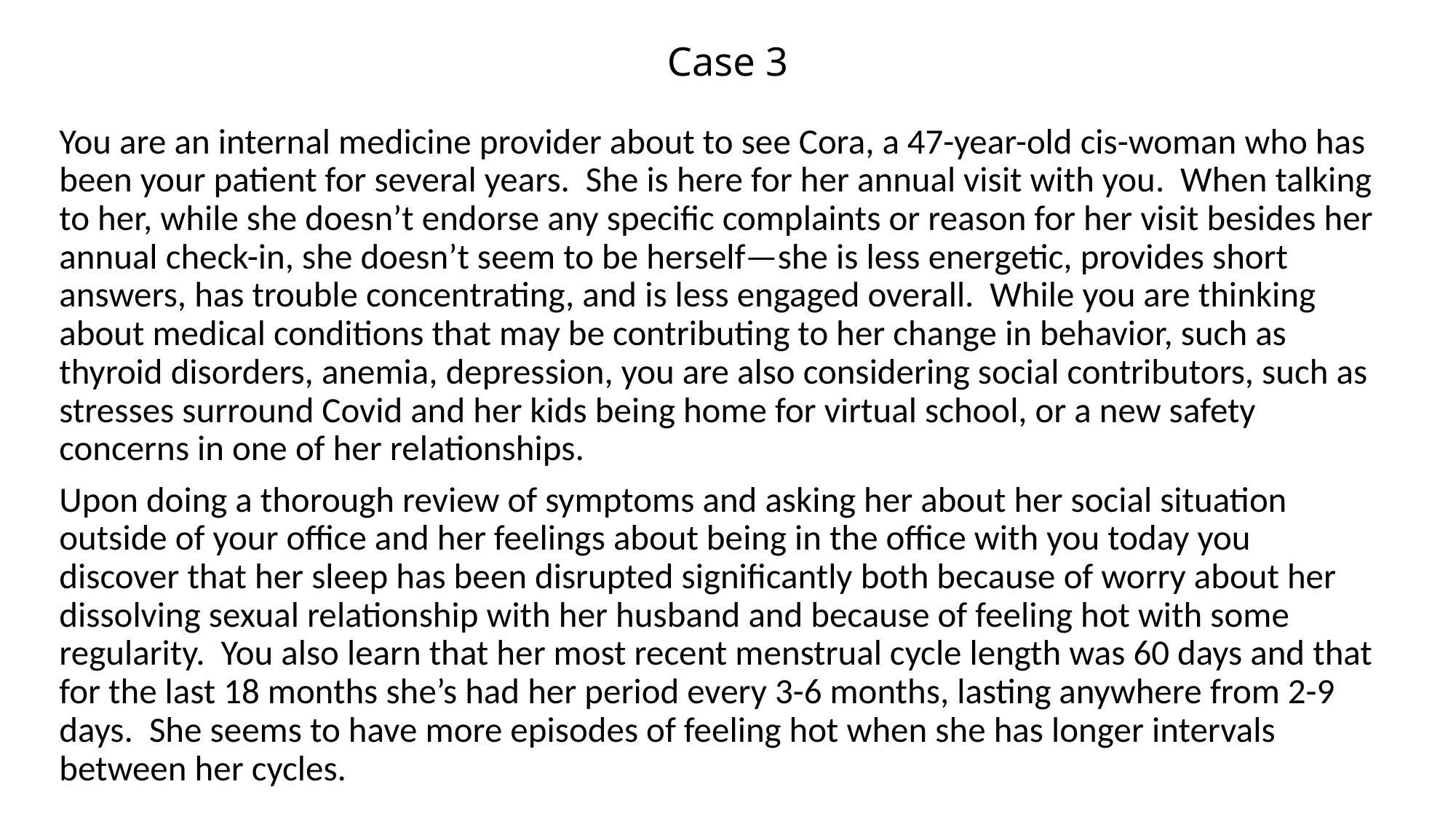

# Case 3
You are an internal medicine provider about to see Cora, a 47-year-old cis-woman who has been your patient for several years. She is here for her annual visit with you. When talking to her, while she doesn’t endorse any specific complaints or reason for her visit besides her annual check-in, she doesn’t seem to be herself—she is less energetic, provides short answers, has trouble concentrating, and is less engaged overall. While you are thinking about medical conditions that may be contributing to her change in behavior, such as thyroid disorders, anemia, depression, you are also considering social contributors, such as stresses surround Covid and her kids being home for virtual school, or a new safety concerns in one of her relationships.
Upon doing a thorough review of symptoms and asking her about her social situation outside of your office and her feelings about being in the office with you today you discover that her sleep has been disrupted significantly both because of worry about her dissolving sexual relationship with her husband and because of feeling hot with some regularity. You also learn that her most recent menstrual cycle length was 60 days and that for the last 18 months she’s had her period every 3-6 months, lasting anywhere from 2-9 days. She seems to have more episodes of feeling hot when she has longer intervals between her cycles.

## Slide 14
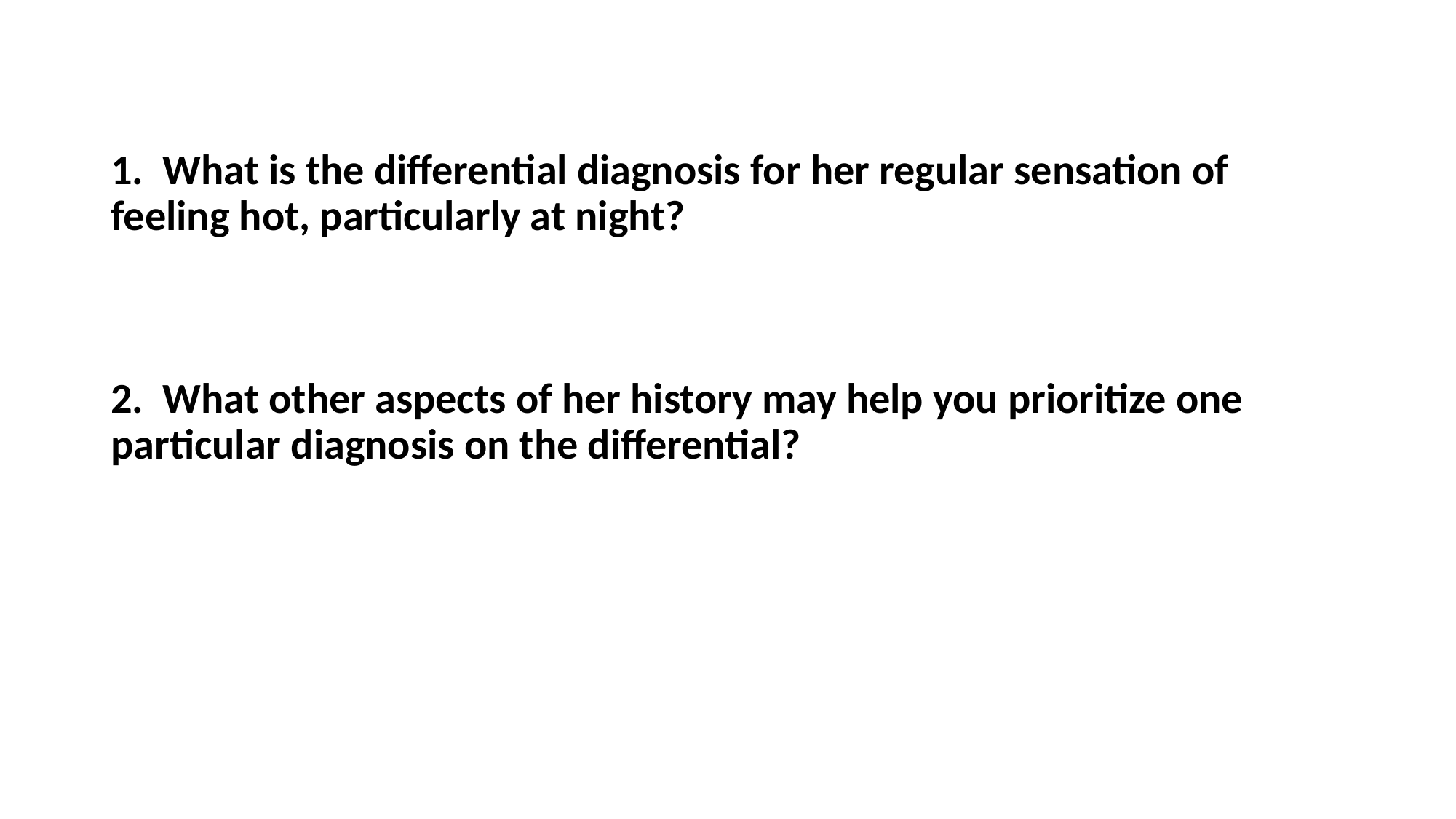

1. What is the differential diagnosis for her regular sensation of feeling hot, particularly at night?
2. What other aspects of her history may help you prioritize one particular diagnosis on the differential?

## Slide 15
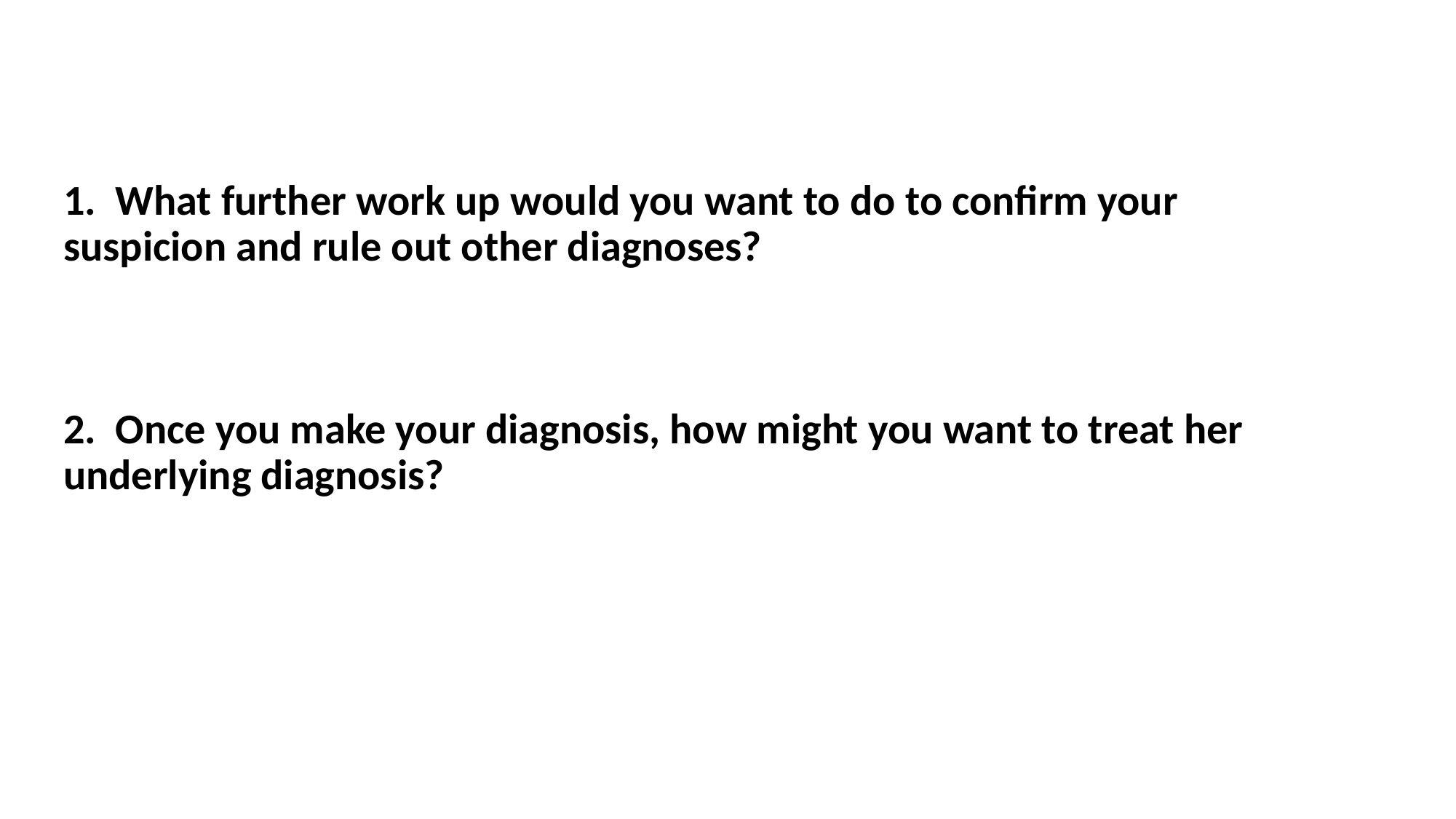

1. What further work up would you want to do to confirm your suspicion and rule out other diagnoses?
2. Once you make your diagnosis, how might you want to treat her underlying diagnosis?

## Slide 16
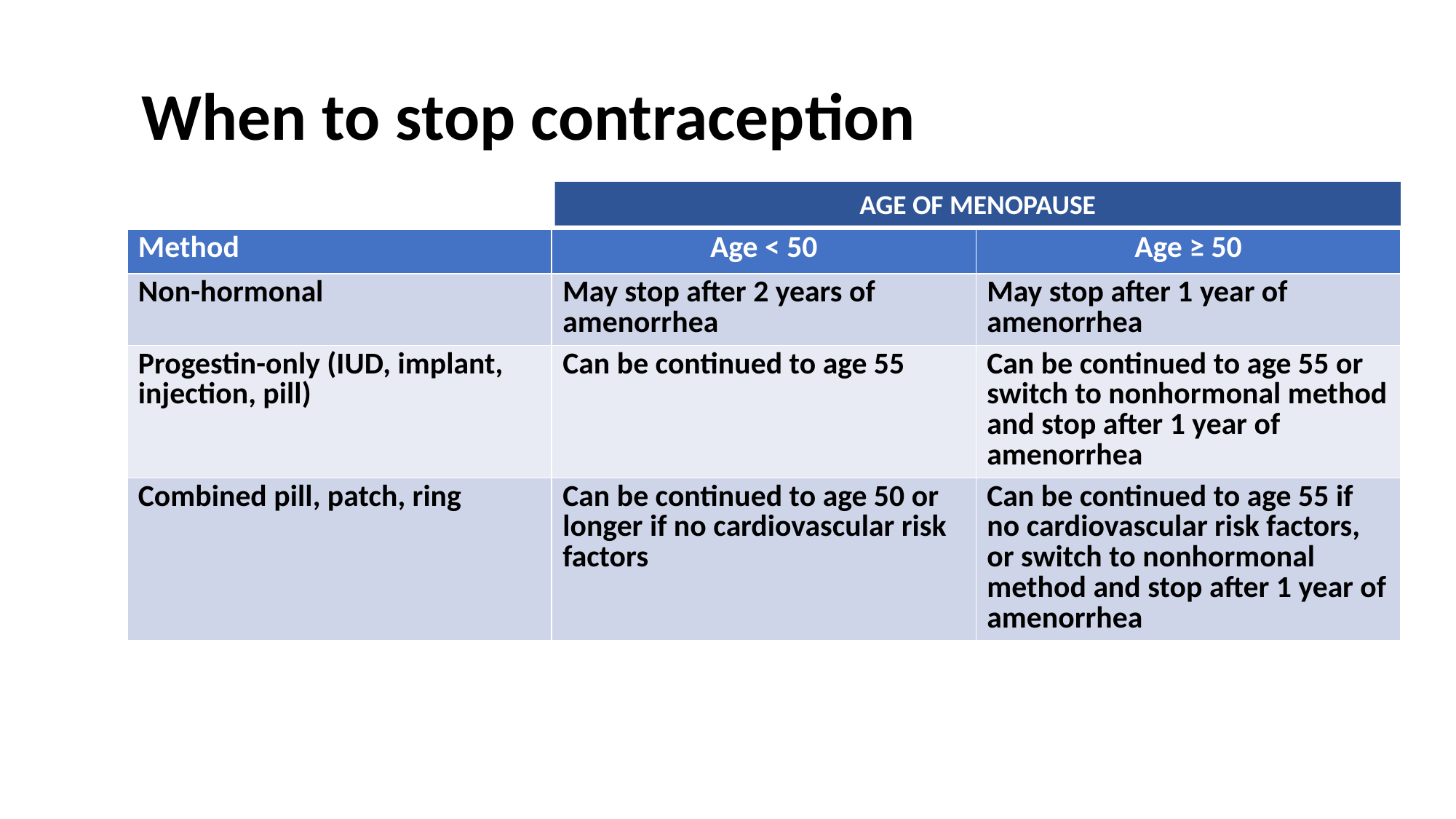

# When to stop contraception
AGE OF MENOPAUSE
| Method | Age < 50 | Age ≥ 50 |
| --- | --- | --- |
| Non-hormonal | May stop after 2 years of amenorrhea | May stop after 1 year of amenorrhea |
| Progestin-only (IUD, implant, injection, pill) | Can be continued to age 55 | Can be continued to age 55 or switch to nonhormonal method and stop after 1 year of amenorrhea |
| Combined pill, patch, ring | Can be continued to age 50 or longer if no cardiovascular risk factors | Can be continued to age 55 if no cardiovascular risk factors, or switch to nonhormonal method and stop after 1 year of amenorrhea |

## Slide 17
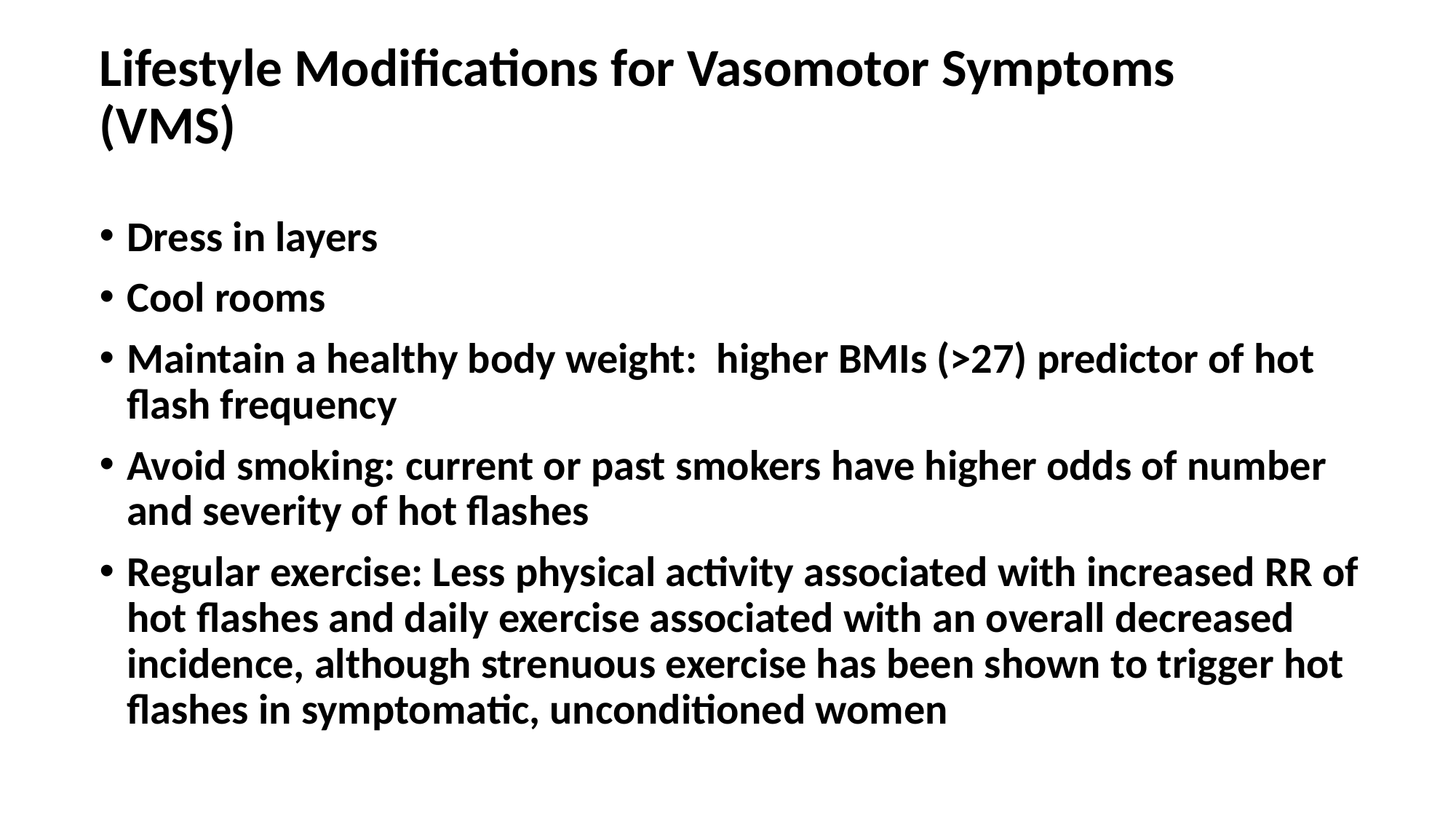

# Lifestyle Modifications for Vasomotor Symptoms (VMS)
Dress in layers
Cool rooms
Maintain a healthy body weight: higher BMIs (>27) predictor of hot flash frequency
Avoid smoking: current or past smokers have higher odds of number and severity of hot flashes
Regular exercise: Less physical activity associated with increased RR of hot flashes and daily exercise associated with an overall decreased incidence, although strenuous exercise has been shown to trigger hot flashes in symptomatic, unconditioned women

## Slide 18
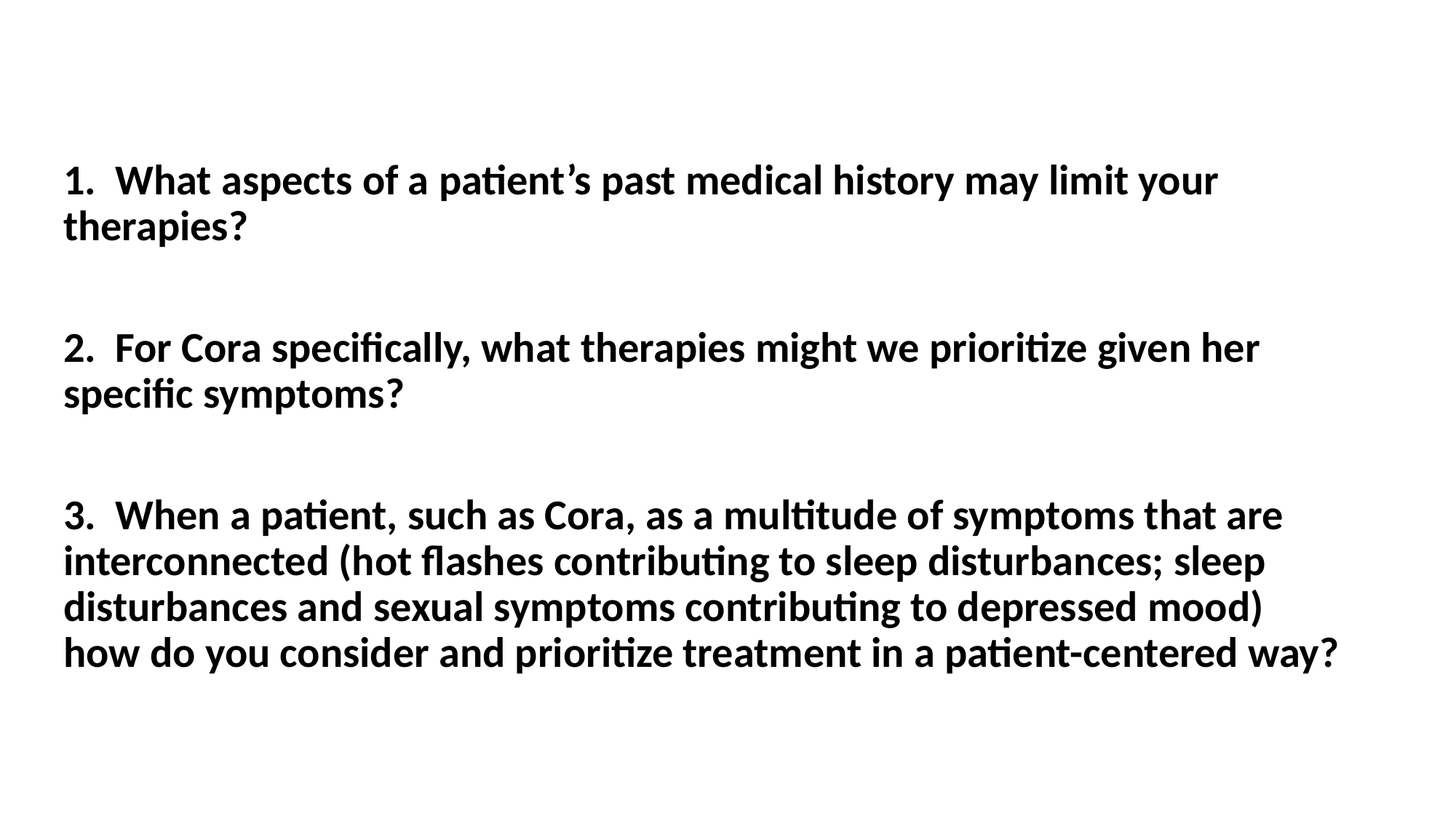

1. What aspects of a patient’s past medical history may limit your therapies?
2. For Cora specifically, what therapies might we prioritize given her specific symptoms?
3. When a patient, such as Cora, as a multitude of symptoms that are interconnected (hot flashes contributing to sleep disturbances; sleep disturbances and sexual symptoms contributing to depressed mood) how do you consider and prioritize treatment in a patient-centered way?

## Slide 19
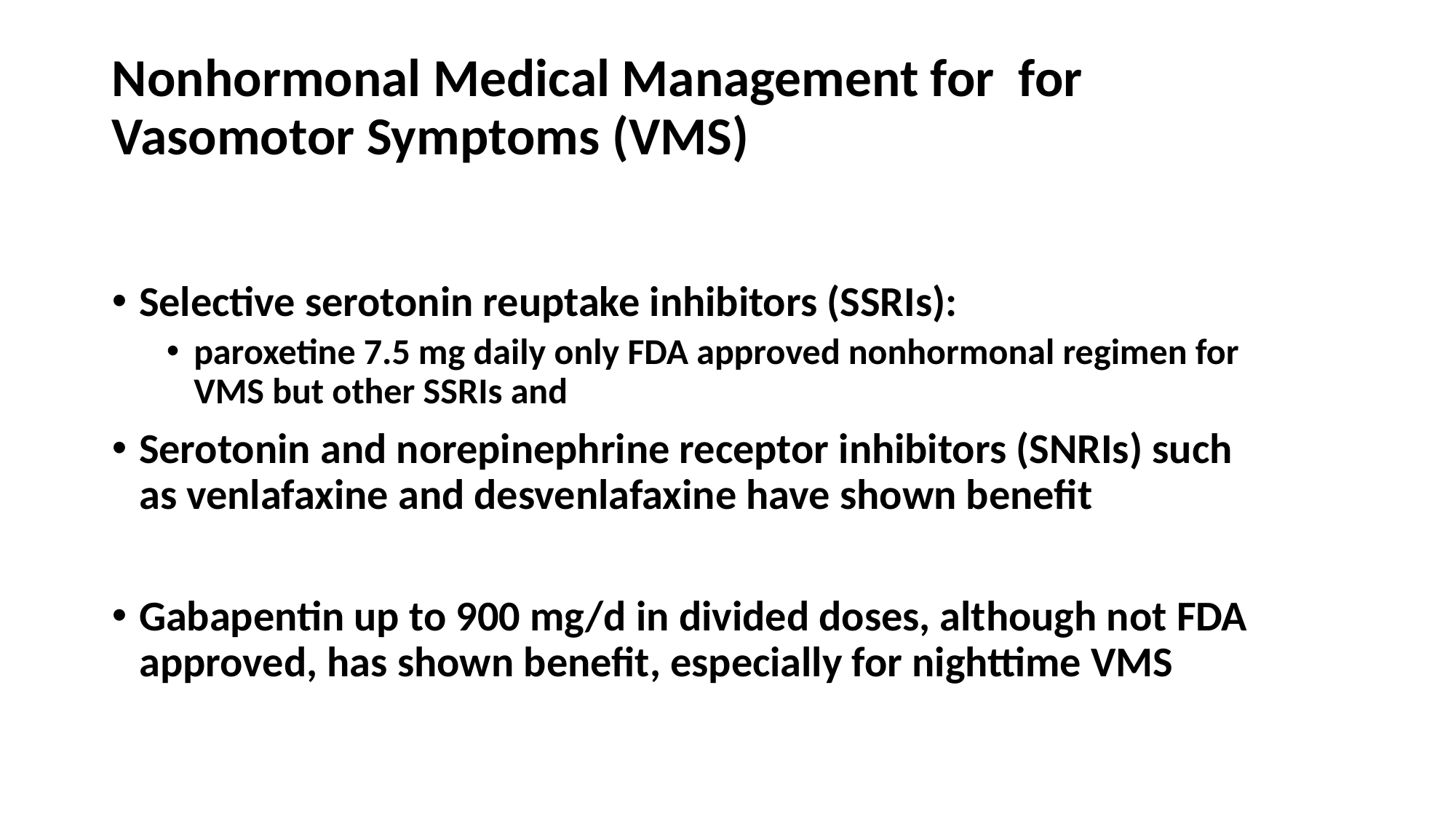

# Nonhormonal Medical Management for for Vasomotor Symptoms (VMS)
Selective serotonin reuptake inhibitors (SSRIs):
paroxetine 7.5 mg daily only FDA approved nonhormonal regimen for VMS but other SSRIs and
Serotonin and norepinephrine receptor inhibitors (SNRIs) such as venlafaxine and desvenlafaxine have shown benefit
Gabapentin up to 900 mg/d in divided doses, although not FDA approved, has shown benefit, especially for nighttime VMS
