# Supplementary material for: Benefits of Hormonal Contraception Across the Lifespan: A Case-Based, Interactive Curriculum
Source: MedEdPORTAL. 2025 Apr 4;21:11512. doi: 10.15766/mep_2374-8265.11512 (PMC11968450; doi:10.15766/mep_2374-8265.11512)
Supplement: Supplementary file 1 — Student Guide and Case 1.docxCase 2.docxCase 3.docxCDC Eligibility Criteria for Contraceptive Use.pdfBN How Well Does Birth Control Work.pdfRHAP Birth Control Across the Gender Spectrum.pdfCounseling for the Hormones Found in Contraceptives.pptxCase-Based Collaborative Learning.pptxFaculty Guide.docxLongitudinal Assessment Questions.docx [file mep_2374-8265.11512-s001.zip › J. Longitudinal Assessment Questions.docx]

Appendix J: This document provides the two qualitative questions we ask our learners in the three to twelve months after the curriculum to study retention, impact on their clerkship experience, and learner perspective on curricular improvements. These questions were asked once, on a paper self-assessment, midway through their OB/GYN clerkship rotation.

**Appendix J. Longitudinal assessment questions**

**Asked on the OB/GYN clerkship**

During your OB/GYN clerkship was your approach to contraceptive counseling of patients guided by the *Reproductive Potential and Gynecologic Needs: A Patient-Centered Approach* didactic session in the Transition to the PCE course? If so, how?

With regards to contraceptive counseling, what did you feel ill-prepared to do on your OB/GYN clerkship that you wish you had better prior instruction on?
