# Supplementary material for: Benefits of Hormonal Contraception Across the Lifespan: A Case-Based, Interactive Curriculum
Source: MedEdPORTAL. 2025 Apr 4;21:11512. doi: 10.15766/mep_2374-8265.11512 (PMC11968450; doi:10.15766/mep_2374-8265.11512)
Supplement: Supplementary file 1 — Student Guide and Case 1.docxCase 2.docxCase 3.docxCDC Eligibility Criteria for Contraceptive Use.pdfBN How Well Does Birth Control Work.pdfRHAP Birth Control Across the Gender Spectrum.pdfCounseling for the Hormones Found in Contraceptives.pptxCase-Based Collaborative Learning.pptxFaculty Guide.docxLongitudinal Assessment Questions.docx [file mep_2374-8265.11512-s001.zip › B. Case 2.docx]

**Small-Group Case 2**

Appendix B: This document serves as the learner guide to the second case-based collaborative learning case dedicated to the noncontraceptive benefits of the hormones in modern contraceptive methods and to trauma-informed care for patients with sex- and gender-minority status. Learners should have access to this case and accompanying questions in time to review in advance of the session. We provide this to our preclinical second-year medical students 2 weeks in advance.

**Cory is a 34-year-old nonbinary patient (assigned female sex at birth, uses they/their/them pronouns) whom you are seeing for the first time. They present to you, their new PCP, because they have been experiencing irregular and heavy menstrual bleeding for 5 months now. Menstrual cramps are typical for them, but they are worsening. Though they have not been to a doctor since they were age 21 years, their periods have gotten to the point that they feel tired and need to do something about it. They do not report any other medical issues. Cory currently works in the food industry. Their only medication is ibuprofen as needed for cramps, but no hormonal therapy. They smoke about 1/2ppd and drink about 5 nights a week.**

**Cory has not seen a physician in 14 years—why might they not be receiving regular medical care?**

**How would you ask them about their main concern today—the heavy, irregular vaginal bleeding and cramping?**

**Because this patient is new to the practice and because it would be helpful to know if they have ever used hormones that could help or worsen these symptoms, you want to ask some questions about sexual identity. When you begin to ask them questions, they say that they do not want to talk about that right now. How might you respond?**

**With time, Cory tells you that they only have cis-gender female partners. They express that the reason they have not been to a doctor since they were age 21 years was because doctors assumed they were “straight” and wanted them to be on birth control pills. Cory is “out” to their family, friends, and coworkers but has avoided medical care. With this new information, can you think again why they may not have received regular medical care since age 21 years?**

**A pelvic exam would be useful to determine the site of the bleeding. However, you recognize that because they have not received medical care in a long time and have had negative experiences surrounding intimate health, they may find the exam physically or emotionally uncomfortable. How would you approach discussion of a pelvic exam utilizing trauma-informed care principles?**

**Based on the patient’s history and your findings on the physical exam, you think they could potentially benefit from the hormones of a hormonal contraceptive method to address their bleeding and cramping. How would you approach this discussion?**

**What other aspects of their health history would you want to discuss further with them?**
