# Supplementary material for: Benefits of Hormonal Contraception Across the Lifespan: A Case-Based, Interactive Curriculum
Source: MedEdPORTAL. 2025 Apr 4;21:11512. doi: 10.15766/mep_2374-8265.11512 (PMC11968450; doi:10.15766/mep_2374-8265.11512)
Supplement: Supplementary file 1 — Student Guide and Case 1.docxCase 2.docxCase 3.docxCDC Eligibility Criteria for Contraceptive Use.pdfBN How Well Does Birth Control Work.pdfRHAP Birth Control Across the Gender Spectrum.pdfCounseling for the Hormones Found in Contraceptives.pptxCase-Based Collaborative Learning.pptxFaculty Guide.docxLongitudinal Assessment Questions.docx [file mep_2374-8265.11512-s001.zip › F. RHAP Birth Control Across the Gender Spectrum.pdf]

Appendix F. Birth control across the gender spectrum infographic about modern birth control method use for those with sex- and gender-minority status. This resource was given to the learners ahead of the curriculum for their review. Learners were encouraged to use this resource throughout their clerkship year as a visual aid during contraceptive counseling visits.

BIRTH CONTROL ACROSS THE GENDER SPECTRUM

CAN YOU GET PREGNANT?

If you have a uterus and ovaries, you can get pregnant. This is true even if you take testosterone. Although it may stop your monthly bleeding, testosterone does not keep you from getting pregnant.

CAN YOU GET SOMEONE PREGNANT?

If you have a penis and testes, you can get someone pregnant. This is true even if you take estrogen. Estrogen may lower your sperm count, but it does not keep you from getting someone pregnant.

BIRTH CONTROL FOR PEOPLE TAKING TESTOSTERONE

People who have a uterus and ovaries and who take testosterone can use any birth control method. The progestin pill, implant, IUD, and shot may help decrease monthly bleeding. Some people use one of these methods just to control bleeding, even if they don't need birth control. Progestin does not interact with testosterone. Many people want to avoid methods with estrogen (pill/patch/ring). It is unclear if estrogen interacts with testosterone. The copper IUD prevents pregnancy and contains no hormones. Condoms prevent pregnancy and sexually transmitted infections (STIs).

BIRTH CONTROL FOR PEOPLE TAKING ESTROGEN

People who have a penis and testes and who take estrogen can use condoms. Their partners can choose any birth control method.

PERMANENT OPTIONS

Permanent methods are great for people who don't ever want to get pregnant. These include tubal ligation, hysterectomy, orchiectomy, and vasectomy.

DON'T FORGET ABOUT SEXUALLY TRANSMITTED INFECTIONS!

Condoms can prevent human immunodeficiency virus (HIV) and other STIs. There are two types of condoms, internal and external. Both types help to prevent pregnancy and infections.

METHODS WITH PROGESTIN

Method	How to Use	Impact on Bleeding	Things to Know	How well does it work?
**Emergency Contraception Pills**<br>Progestin EC (Plan B One-Step® and others) and ulipristal acetate (ella®)<br>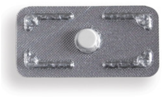	Works best the sooner you take it after unprotected sex.<br>You can take EC up to 5 days after unprotected sex.<br>If pack contains 2 pills, take both together.	May cause stomach upset or nausea.<br>Your next monthly bleeding may come early or late.<br>May cause spotting.	Available at pharmacies, health centers, or health care providers: call ahead to see if they have it.<br>People of any age can get progestin EC without a prescription, and it doesn't interact with testosterone.<br>May cause stomach upset or nausea.<br>Ulipristal acetate EC requires a prescription and we don't know whether or not it interacts with testosterone.<br>May cost a lot.<br>Ulipristal acetate EC works better than progestin EC if your body mass index (BMI) is over 26.<br>Ulipristal acetate EC works better than progestin EC 3-5 days after sex.	58-94%

over →

Method	How to Use	Impact on Bleeding	Things to Know	How well does it work?
**Hormonal IUD**<br>Liletta®, Mirena®, Skyla® and others<br>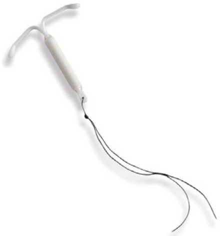	A clinician places it in the uterus.<br>It is usually removed by a clinician.	May cause spotting.<br>It may improve monthly bleeding and cramps.<br>After 1 year, you may have no monthly bleeding at all.	It works for 3 to 8 years, depending on which IUD you choose.<br>You may become pregnant right after removal.<br>It may lower the risk of uterine lining cancer, ovarian cancer, and polycystic ovary syndrome (PCOS).	> 99%
**The Implant**<br>Nexplanon®<br>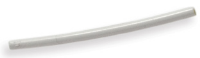	A clinician places it under the skin of the upper arm.<br>It must be removed by a clinician.	May cause spotting.<br>After 1 year, you may have no monthly bleeding at all.	It may last up to 5 years.<br>It often decreases cramps.<br>It may lower the risk of uterine lining cancer, ovarian cancer, and polycystic ovary syndrome (PCOS).<br>It may cause mood changes.	> 99%
**Progestin-Only Pills**<br>Camila, Nor-QD®<br>Micronor<br>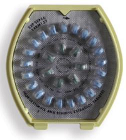	Take the pill at the same time daily.	Can make monthly bleeding more regular and less painful.<br>May cause spotting the first few months.	You can become pregnant right after stopping the pills.<br>It may cause depression, hair or skin changes, or change in sex drive.<br>It may lower the risk of uterine lining cancer, ovarian cancer, and polycystic ovary syndrome (PCOS).	93%
**The Shot**<br>Depo-Provera®<br>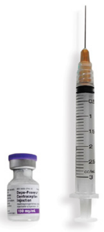	Get a shot every 3 months.<br>You can get the shot at a health care office, or you can give yourself the shot.	It usually decreases monthly bleeding.<br>After 1 year, you may have no monthly bleeding at all.	Each shot works for 12 weeks.<br>It may lower the risk of uterine lining cancer, ovarian cancer, and polycystic ovary syndrome (PCOS).<br>It may cause spotting, weight gain, depression, hair or skin changes, or change in sex drive.<br>It may cause delay in getting pregnant after you stop the shots.<br>Side effects may last up to 6 months after you stop the shots.	96%

continued →

BIRTH CONTROL ACROSS THE GENDER SPECTRUM

METHODS WITH NO HORMONES

Method	How to Use	Impact on Bleeding	Things to Know	How well does it work?
**External Condom** 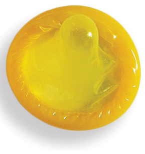	Use a new condom each time you have sex.<br>Use a polyurethane condom if allergic to latex.	None	Can buy at many stores.<br>Can put on as part of sex play/foreplay.<br>Can help prevent early ejaculation.<br>Can be used for oral, vaginal, and anal sex.<br>Can decrease sensation.<br>Can cause loss of erection.<br>Can break or slip off.	87%
**Internal Condom** 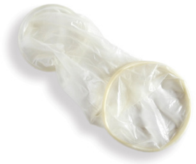	Use a new condom each time you have sex.<br>Use lubrication as needed.	None	Can put in as part of sex play/foreplay.<br>Can be used for anal and vaginal sex.<br>May increase pleasure.<br>Good for people with latex allergy.<br>Can decrease sensation.<br>May be noisy.<br>May be hard to insert.<br>May slip out of place during sex.<br>May need to purchase from distributor or get a prescription from your clinician.	79%
**Diaphragm**<br>Caya® and Milex® 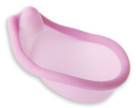	Put in vagina each time you have sex.<br>Use with spermicide every time.	None	Can last several years.<br>Costs very little to use.<br>May protect against some infections, but **not HIV.**<br>Using spermicide may raise risk of getting HIV.<br>Should not be used with vaginal bleeding or infection.<br>Raises risk of bladder infection.	78%
**Copper IUD**<br>ParaGard® 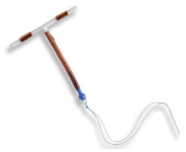	A clinician places it in the uterus.<br>It is usually removed by a clinician.	May cause spotting (if you are taking testosterone, this may not be an issue for you).	May be left in place for up to 12 years.<br>You can become pregnant right after removal.<br>It may lower the risk of uterine lining cancer, ovarian cancer, and polycystic ovary syndrome (PCOS).	> 99%
**Vaginal Acidifying Gel**<br>Phexxi® 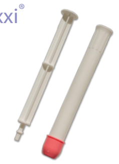	Insert gel each time you have sex.	None	Can be put in as part of sex play/foreplay.<br>Does not have any hormones.<br>Requires a prescription.<br>May irritate vagina, penis.<br>Should not be used with urinary tract infection.	86%

over →

**Vaginal Spermicide**<br>Cream, gel, sponge, foam, inserts, film<br>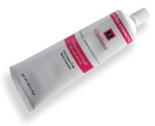	Insert spermicide each time you have sex.	None	Can buy at many stores.<br>Can insert as part of sex play/foreplay.<br>Comes in many forms: cream, gel, sponge, foam, inserts, film.<br>May raise the risk of getting HIV.<br>May irritate vagina, penis.<br>Cream, gel, and foam can be messy.<br>Does not require a prescription.	79%
**Withdrawal**<br>Pull-out	Pull penis out of vagina before ejaculation (that is, before coming).	None	Costs nothing.<br>Less pleasure for some.<br>Does not work if penis is not pulled out in time.<br>Must interrupt sex.	80%

METHODS WITH ESTROGEN

Some people prefer to avoid methods with estrogen, but they are perfectly safe to use – even if you are taking testosterone.

Method	How to Use	Impact on Bleeding	Things to Know	How well does it work?
**The Patch**<br>Ortho Evra®<br>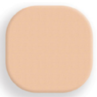	Apply a new patch once a week for three weeks.<br>No patch in week 4.	Can make monthly bleeding more regular and less painful.<br>May cause spotting the first few months.	You can become pregnant right after stopping the patch.<br>Can irritate skin under the patch.	93%
**The Pill**<br>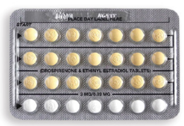	Take the pill daily.	Often causes spotting, which may last for many months.	Can improve PMS symptoms.<br>Can improve acne.<br>Helps prevent cancer of the ovaries.<br>You can become pregnant right after stopping the pills.<br>May cause nausea, headaches, change in sex drive – some of these can be relieved by changing to a new brand.	93%
**The Ring**<br>ANNOVERA®,<br>Nuvaring®<br>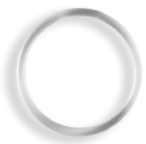	Insert a small ring into the vagina.<br>Monthly Ring: Change ring each month.<br>Yearly Ring: Change ring each year.	Can make monthly bleeding more regular and less painful.<br>May cause spotting the first few months.<br>Can increase vaginal discharge.	There are 2 types: a monthly ring and a yearly ring.<br>One size fits all.<br>Private.<br>You can become pregnant right after stopping the ring.	93%

Image by Reproductive Health Access Project, retrieved from: https://www.reproductiveaccess.org/resource/birth-control-across-the-gender-spectrum/on 11/11/2023. Creative Commons License associated: https://creativecommons.org/licenses/by-nc-sa/4.0/.

reproductive health access project
