# Supplementary material for: Benefits of Hormonal Contraception Across the Lifespan: A Case-Based, Interactive Curriculum
Source: MedEdPORTAL. 2025 Apr 4;21:11512. doi: 10.15766/mep_2374-8265.11512 (PMC11968450; doi:10.15766/mep_2374-8265.11512)
Supplement: Supplementary file 1 — Student Guide and Case 1.docxCase 2.docxCase 3.docxCDC Eligibility Criteria for Contraceptive Use.pdfBN How Well Does Birth Control Work.pdfRHAP Birth Control Across the Gender Spectrum.pdfCounseling for the Hormones Found in Contraceptives.pptxCase-Based Collaborative Learning.pptxFaculty Guide.docxLongitudinal Assessment Questions.docx [file mep_2374-8265.11512-s001.zip › I. Faculty Guide.docx]

**Faculty Guide**

Appendix I: This document serves as the faculty guide to the curriculum overall, with specific details given to assist in facilitating the case-based collaborative learning in the small group (40-50 student) learning studios. This document perfectly mirrors the Student Guide (Appendix A, B, and C) with extra content that includes 1.) teaching tips overall, 2.) answers to the discussion questions, and 3.) some broad, concluding remarks. Faculty should familiarize themselves with this content ahead of time and should have it available as a reference during the case-based collaborative learning session.

**Reproductive Potential and Gynecologic Needs: A Patient-Centered Approach**

**Overview**: Patients’ reproductive and gynecologic needs affect their lives, their health, and their interface with the healthcare system. The specific aim of this course will be to examine common patient encounters and health care needs that are currently often situated within gynecology alone but must instead be considered within multiple health care disciplines. Within this learning we will emphasize a patient-centered approach, with an emphasis on individual patient social contexts that may affect the health care encounter and health care decision-making.

**By the end of this activity, learners will be able to:**

1. Identify the need for and the considerations involved in providing family planning as part of the medical care plan for pregnancy-capable individuals at time of a new diagnosis of a chronic medical condition.
2. Using a trauma-informed care approach, describe assumptions, language, and gynecology health care practices which may contribute to medical mistrust.
3. Describe practice improvements to mitigate medical mistrust for a diversity of patients across the gender identity spectrum.
4. Describe the approach to history taking for perimenopausal patients.
5. Describe the role of exogenous hormones in perimenopausal symptom management.

**Agenda:**

1:00-1:40 Contraceptive Counseling: A Patient-Centered Approach

1:40-2:00 BREAK to get to Learning Labs

2:00 to 4:00 Case-based Collaborative Learning within Learning Studios

2:00-2:35 Case 1: Reproductive potential in the setting of chronic disease

2:40-3:15 Case 2: Building trust and a patient-centered practice in gynecology

3:20-3:55 Case 3: Care in patients at the end of the reproductive life course

**Preparatory Work:**

1. Read the three small group cases and their associated questions

Additional Resources for the Principal Clerkship Experience:

1. CDC. 2024 Summary Chart of US Medical Eligibility Criteria for Contraceptive Use.^1^ Please recognize this resource is updated on occasion. Look for updates every 2 years when using this resource in your future clinical practice. (Appendix D)
2. Bedsider Birth Control Network. Birth Control Methods Comparative Chart.^2^ (Appendix E)
3. Reproductive Health Access Project. Birth Control Across the Gender Spectrum.^3^ (Appendix F)

^1^Image by CDC, retrieved on November 11, 2023 from: https://www.cdc.gov/contraception/media/pdfs/2024/07/us-mec-summary-chart-color-508.pdf?mc_cid=d8d9bc1493&mc_eid=69a2a30a0f. Image is in the public domain. Data summarized from Nguyen AT, Curtis KM, Tepper NK, et al. U.S. Medical Eligibility Criteria for Contraceptive Use, 2024. MMWR Recomm Rep 2024;73(No. RR-4):1–126. DOI: <http://dx.doi.org/10.15585/mmwr.rr7304a1>

^2^Image by UCSF School of Medicine Bixby Center, retrieved on November 11, 2023 from: https://www.bedsider.org/. Creative Commons attribution NonCommercial - NoDeriv 3.0 Unported License.

^3^Image by Reproductive Health Access Project, retrieved on November 11, 2023 from: https://www.reproductiveaccess.org/resource/birth-control-across-the-gender-spectrum/. Creative Commons License associated: https:// creativecommons.org/licenses/by-nc-sa/4.0

**Case-Based Collaborative Learning Teaching Tips for Faculty:**

- Students have been given the cases and questions ahead of time with the recommendation that they start to think through them to facilitate the small-group discussions
- Prior to the small-group session you will be leading, the students have had a lecture about contraception counseling and provision.
- The student copy of the cases does not have the bulleted content embedded in the cases like your copy has below. That is specifically for you as teaching faculty to help facilitate the learning.
- You will have 2 hours with students who know each other well.
- Students are used to working through problems and cases with their peers in these learning studios.
- Time is short. You likely will not have time to get introductions from the students, but you can introduce yourself.
- Each case will have 35 minutes dedicated to it.
- Be strict with time. Try to move on to the next case at the 25-minute mark.
- Consider trying to use gender-neutral language, since many students advocate for greater inclusivity of the full spectrum of patients who need OB/GYN care. However, mistakes are going to be made. We often start these teaching sessions with this disclosure, “I am going to try to use gender-neutral terms but I sometimes forget. If I do, I apologize as our whole specialty is trying to convert to greater inclusivity and this is an ongoing learning process for me.”
- To this last point, if someone corrects you or challenges you, start with “thank you for bringing this to my attention” as a way to pause and think for a moment. If their point is valid, apologize and/or thank them for bringing whatever this issue is up. If you feel the need to push back, do so without being defensive or coming from a place of emotion. If they are just plain wrong and you feel confident in that, you can do some teaching on their point. However, please do this in the most open way possible so they are not embarrassed. If you do not know the answer to what they are saying or you are not completely confident, it is okay to say you are going to investigate something further/look something up. You can even offer to be in touch after the class and keep it an ongoing conversation.
- More teaching tips are included in the Speaker Notes of the CBCL slides (Appendix H: CBCL.Slides)

**Small-Group Case 1**

**Suyin is a 28-year-old cis-gender female who comes to the clinic with her husband. As she explains her symptoms, she appears nervous. She describes how a few weeks ago, she started to experience joint stiffness in her fingers, wrists, and knees. She experiences pain upon bending the affected joints on both sides of the body. The stiffness and pain are worst in the morning and take about 2 to 3 hours to dispel. She is concerned because she is feeling very fatigued, and it is difficult for her to go about her daily activities.**

**Her history, a physical exam, and abnormal findings in bloodwork are consistent with a diagnosis of rheumatoid arthritis (RA).** **What do you think about this diagnosis in this patient? Does it seem plausible? Are there any special considerations to be made for this particular patient with this particular diagnosis?**

- Consider sex: Females are at higher risk for autoimmune disease than males, and most autoimmune diseases have a female preponderance. For example, systemic autoimmune erythematosus occurs in a 9:1 female:male ratio, and multiple sclerosis at a 3:1 ratio. RA is also female-predominant at a ratio of 2-3:1. Genetic factors contribute to risk—several pro-inflammatory genes are on the X-chromosome.
- Consider age: Autoimmune diseases are most commonly diagnosed during the reproductive years. Sex hormone fluctuation is thought to play a role. Generally, androgens are anti-inflammatory. Estrogen differentially affects different arms of the immune system, but the high estrogen environment of pregnancy tends to be anti-inflammatory. Pregnancy has protective effects against a number of autoimmune diseases including multiple sclerosis and rheumatoid arthritis but can worsen symptoms in systemic lupus erythematosus.
- Overall, the diagnosis of RA seems plausible.

**You consider prescribing methotrexate, a common first-line treatment for RA. Methotrexate begins to relieve symptoms relatively quickly and has a lower cost than most other options. Adverse drug events are relatively rare, although patients should limit alcohol intake. When considering whether to prescribe the drug, what information would further help you decide?**

- Reproductive plans and risk of pregnancy is the main concern here regarding putting this patient on a teratogenic medication.
- Other considerations: whether the patient has a history of hepatic disease or alcoholism; whether the patient is currently pregnant or breastfeeding; whether the patient is open to medication; if there is any influence or concern for abuse by partner (to influence taking or not taking of meds); cultural beliefs/history of medicine for the patient historically, by culture, race, and gender; patient knowledge of her body and side effect tolerance; full disclosure on side effects and questions—make time for this (collaboration, empowerment, transparency).

**You suggest starting on methotrexate to Suyin, and she asks about side effects. You explain that GI symptoms are the most frequent adverse effects, that the drug could affect liver function at high doses, and that it is teratogenic. When you explain teratogenicity, the patient looks concerned. How would you continue the conversation?**

- Here, it would be beneficial to pause and ask the patient about her concerns. It is important to have a conversation with her about her reproductive life plans, including whether she desires pregnancy in the future or not. Ascertaining her desire to become pregnant in the future is important, as it can influence how you recommend managing her chronic disease and medications. Keep in mind that thinking about pregnancy in the context of chronic disease can be anxiety-inducing for the patient–instead of pressuring her one way or the other, it is important to explain the full range of possibilities for her so she can make informed decisions.
- Take time here, patient learning something new and overwhelming, build in time for reaction, tears, thoughts and questions, be ready for a possible trauma reaction. Be ready to schedule follow-up to address further.

**You ask Suyin if she thinks she might like to become pregnant in the next year, and she responds that she is not sure. She is not currently trying to become pregnant.**

**Given this information, would you still prescribe a teratogenic medication? How would you counsel her?**

- Preconception counseling considerations
  - Review stage and activity of the disease. Patients with active disease may consider postponing pregnancy until they are in remission, or their disease is stable.
  - Review all medications the patient may be taking, including but not limited to the teratogens, and potentially adjust therapy. Some drugs to manage RA symptoms may pose less of a risk during pregnancy as compared to active disease. Pregnancy can lessen some symptoms of RA, which may affect considerations for drug type and dose. However, women can experience flares in the postpartum.
  - Review the possibility of reduced fertility. Has she had trouble becoming pregnant in the past?
- Contraception counseling considerations
  - Women suffering from chronic diseases are less likely to use contraception and less likely to receive contraceptive counseling than healthy women (Schwarz JGIM 2012, Steinkellner Am J Med 2010, Yazdany AC&R 2011)
  - In the lecture before these students hear an example from seizure clinics—women with seizure suffer from unintended pregnancy at the same rate as the general population indicating they are not being told to prevent pregnancy any better than baseline despite being on teratogenic meds and having pregnancy worsen their primary disease of seizure frequency.
  - Ask the patient if she is currently using contraception, keeping in mind that she can still benefit from contraception counseling even if she is already using a method of birth control.
  - If yes: Consider whether there are contraindications to this form of contraception and her chronic disease (for RA, no form of contraception, including emergency contraception, is contraindicated unless the patient has antiphospholipid antibodies). Ask if she is comfortable continuing her current method or if she would like to learn about other options. If taking a teratogenic drug, she may feel more comfortable using one of the more effective LARC methods.
  - If no: If the patient is not using birth control and does not wish to start, and she is at risk of pregnancy, explore different options for therapy aside from teratogenic drugs for her primary RA disease.

**Because she is not comfortable using hormonal birth control, Suyin states that she always uses condoms. Would your counseling change if she decided she did not want to become pregnant in the next year but only would like to use barrier methods of contraception?**

- Ethical considerations in balancing reproductive autonomy and risk management–case of Accutane–want to avoid contraceptive coercion but also want to make sure that the patient is fully informed about risk. Patient-centered contraceptive counseling should always be the goal, though it may sometimes be important to stress the importance of a highly effective birth control method. Options for continued care in the setting of a desire for a third-tier birth control method while on a teratogenic medication include 1) switching the care plan to a less concerning medication than methotrexate if possible or 2) give the patient more information for her to review about highly effective birth control methods and ask to see her back within a short time frame (such as 1 month) to check in on symptoms and her new methotrexate regimen, but also to readdress birth control options.
- One counseling consideration may be to let her know that uncontrolled chronic medical conditions may worsen in pregnancy or may have mal-effects on the pregnancy/baby itself (not so much RA, but conditions such as a seizure disorder or diabetes may lead to increased risk of stillbirth/miscarriage or fetal malformations, respectively). Therefore, we usually like to get new diagnoses “under control” before a patient gets into the more complicated state of having a new diagnosis AND a pregnancy.
- You could also talk a bit about reproductive autonomy. That while a decision on whether or not to get pregnant at this time might be seen as “bad” by us as health care providers, many patients prioritize things differently. Furthermore, many patients “plan” less than Type A physicians/clinicians and many have the attitude of “if it happens, it happens” surrounding pregnancy. As physicians we really like to “get control of situations” as a means of trying to get to the best outcome. Patients’ decisions surrounding reproductive planning may be in direct conflict with our desire to “control” the situation and we need let the patient make what we might deem as a “bad” decision, but we need to keep those visits “patient centered.” However, that may be a circumstance where methotrexate falls down on the list of RA treatment options to minimize risk of exposure in the setting of an unplanned pregnancy.

*****Bottom line to impress on the students**: unless they are going into neonatology or geriatrics, these future doctors are going to be taking care of women and pregnancy-capable sex- and gender-minority patients of reproductive age. Pregnancy affects the primary disease, and the primary disease often affects the pregnancy. Therefore, pregnancy planning and contraceptive management needs to be a part of ALL care plans when they make a new medical diagnosis!

**Small-Group Case 2**

**Cory is a 34-year-old nonbinary patient (assigned female sex at birth, uses they/their/them pronouns) whom you are seeing for the first time. They present to you, their new PCP, because they have been experiencing irregular and heavy menstrual bleeding for 5 months now. Menstrual cramps are typical for them, but they are worsening. Though they have not been to a doctor since they were age 21 years, their periods have gotten to the point that they feel tired and need to do something about it. They do not report any other medical issues. Cory currently works in the food industry. Their only medication is ibuprofen as needed for cramps, but no hormonal therapy. They smoke about 1/2ppd and drink about 5 nights a week.**

Tips to share with the students

- Trauma exposure is highly prevalent in trans and gender diverse (TGD) communities.
- TGD patients with identities subject to additional oppressions (race, class, immigration status, etc.) are at increased risk for trauma exposure.
- Exposure to trauma and gender-minority stress may be associated with both adverse health effects and development of resilience.
- Trauma-informed care (TIC) provides a model for responding to trauma appropriately, reducing retraumatization, and setting the stage for healing and recovery
- TIC is guided by 6 principles: safety; trustworthiness and transparency; peer support; collaboration and mutuality; empowerment, voice, and choice; and awareness of cultural, historical, and gender issues.
- Universal application of TIC principles is essential when caring for TGD patients and includes:
  - Affirming the patient’s gender throughout the encounter
  - Attending to power dynamics throughout the encounter
  - Obtaining the history in a patient-led manner
  - Asking about trauma in a manner that resists retraumatization
  - Responding appropriately to trauma disclosure
  - Performing the physical examination in a collaborative manner that resists retraumatization
  - Recognizing symptoms and exam findings that may suggest a history of trauma
  - Recognizing and responding effectively when the patient becomes distressed
  - Co-developing care plans that are patient empowering and enable mutual respect, safety, and ongoing engagement.
  - Facilitating connection to TGD-sensitive trauma recovery services
  - Recognizing, celebrating, and building on the patient’s strengths over time
  - Continuously attending to the self care of the clinician
- TIC principles apply to both TGD patient/clinician encounters as well as to institutional and community/societal responses to TGD patient populations.
- Clinicians can play a key role in advocating for TIC at the systems level to ensure that the health care sector is fully responsive to the needs of TGD patients.

**Cory has not seen a physician in 14 years – why might they not be receiving regular medical care?**

- There are myriad barriers to care that can result in patients’ restricted access to care or intentional decision to avoid health systems: issues of insurance coverage, financial hardship, difficulty with transportation, language barriers.
- However, negative past experiences with the medical system are among the social and cultural influences that could contribute, especially in patients who may have gender identities that do not conform to their organs and sex assigned at birth.
- This is especially true if a physician just makes assumptions about gender identity and sexual behaviors based on organs and sex assigned at birth. Asking all patients this information early on (such as at registration) normalizes this practice for all your patients, minimizes feelings of stigma and bias by just asking those questions to patients who “look” like they may have a gender identity that is different from sex assigned at birth, and minimizes the risk of missing patients who have identities different than your assumptions.
- Since the practice of medicine with an eye towards recognizing gender diversity may be a new practice for many physicians, gender-diverse patients often find themselves in the position of “teaching” their physicians. Physicians may think they are being “kind, interested, compassionate” by asking a lot of questions about how to provide best care to an individual patient, but patients with sex- and gender-minority status can get fatigued teaching physicians and feeling like a “test” patient or “Guinea pig” who has to represent all of gender diversity.
- Use TIC to be open, aware, and transparent, do not try to be knowledgeable if you are not, be upfront, be open, correct mistakes if they happen in real time.

**How would you ask them about their main concern today—the heavy, irregular vaginal bleeding and cramping?**

- There are many causes of abnormal vaginal bleeding. To approach the diagnosis, it is helpful to narrow down the possibilities by ascertaining a) what portion of the menstrual cycle the patient is in, b) whether the bleeding is occurring in the context of an ovulatory cycle, and c) the pattern of abnormal bleeding. At 35 years old, it is unlikely that the patient is premenopausal. If the patient does not describe bleeding outside of their menstrual cycle and has menstrual cycles of 21-45 days, they are likely experiencing ovulatory cycles. To learn more about the bleeding pattern, possible questions include how often the patient has their period, how long the period lasts, and how often they have to change pad/tampon during heaviest flow. Knowing if they have ever had these symptoms before would be helpful.
- ask what anatomical terms the pt prefers rather than assuming vagina is the right term, be aware talking about menses, ovaries can be triggering to patient both if history of sexual abuse, and for body dysmorphic people in TGD communities.

**Because this patient is new to the practice and because it would be helpful to know if they have ever used hormones that could help or worsen these symptoms, you want to ask some questions sexual identity. When you begin to ask them questions, they say that they do not want to talk about that right now. How might you respond?**

- TIC-“Thank you for letting me know, I will not ask about this now. If you want to know the medical reasons I am interested in this history let me know. This is your visit and your body, how else can I help today?”
- Making assumptions about patients’ gender identity and sexual health can have a negative impact on your relationship with the patient and your ability to best address their problem. Emphasize to the patient that you are asking these questions because they are important to overall health and it is something you ask of everyone. Knowing aspects of their gender identity and sexual history could help you provide better care, as it may help you know how you can individualize an exam and a treatment plan that may be as individualistic/targeted to Cory themself. Emphasizing that you ask these questions to all of your patients so that they do not feel singled out.
- Tips for establishing trust with patients:
  - Don’t ask invasive questions that are not necessary and, if you think they are necessary, explain to the patient why you are asking them
  - Educate yourself about health care for sex and gender minorities as a means of minimizing bias and disparate care
  - Avoid assumptions about gender identity, language, patterns and behaviors by asking open-ended questions
  - Speak openly and without judgment about gender, health, sexuality and mental health
  - Ask for consent before each step of an exam or procedure. Check-in with your patient as much as possible; place the control in your patient’s hands and tell them very overtly that they are in control

**With time, Cory tells you that they only have cis-gender female partners. They express that the reason they have not been to a doctor since they were age 21 years was because doctors assumed they were “straight” and wanted them to be on birth control pills. Cory is “out” to their family, friends, and coworkers but has avoided medical care. With this new information, can you think again why they may not have received regular medical care since age 21 years?**

- Many patients who identify as queer face overt discrimination from health care systems and providers, and high numbers report being turned away from care due to their sexual or gender identity. For those with access to care, implicit bias and microaggressions can negatively affect their experiences by reinforcing stereotypes or communicating disapproval. Cory’s previous provider assumed that they were having sex with men. Cory may have lost trust in the provider, contributing to their decision not to return. Even if they had continued to receive care, the bias might lead their provider to not offer appropriate screening or counseling regarding sexual health.

They might also have had a past traumatic experience that would make a future pelvic exam challenging (i.e., sex- and gender-minority individuals are more likely to have experienced childhood sexual abuse and intimate partner violence).

They might also have had a past traumatic experience during a pelvic exam itself. Sex- and gender-minority individuals in particular may find having a pelvic exam to be a gender dysphoric experience and may utilize gender-neutral terms when referring to body parts. It is a good idea to ask what terms a patient uses and remember to use those terms throughout the encounter, using the same terms they use for their own anatomy.

- Because of this avoidance, sex- and gender-minority patients have higher rates of undiagnosed a) cervical dysplasia that could lead to cancer and b) STIs that could lead to worse upper genital track sequelae if not treated. Lack of diagnosis of these conditions due to both health care avoidance and due to a patient and provider lack of understanding of risk. Unplanned pregnancy can also happen within this population for the same reasons.
  - A relationship of trust and transparent communication can help mitigate this risk

**A pelvic exam would be useful to determine the site of the bleeding. However, you recognize that because they have not received medical care in a long time and have had negative experiences surrounding intimate health, they may find the exam physically or emotionally uncomfortable. How would you approach discussion of a pelvic exam utilizing trauma-informed care principles?**

- Trauma-informed care is a universal approach, so the techniques can be employed with all patients, including those who have not discussed a history of trauma. There are a number of measures you can take to help the patient feel safer during a pelvic exam or pap test. Some measures that can increase Cory’s comfort are: letting them know they can make a follow-up appointment to have the exam and bring a support person, using a chaperone, allowing patients to handle a speculum beforehand, and keeping patients as covered as possible, including letting them know they can keep their shirts on. Let the patient know that you can stop the exam at any time if they ask or raise their hand. When asking patients about their preferences, it is important to ask open-ended questions so the patient can explain beyond yes/no.

**Based on the patient’s history and your findings on the physical exam, you think they could potentially benefit from the hormones of a hormonal contraceptive method to address their bleeding and cramping. How would you approach this discussion?**

- The patient has already expressed frustration with previous providers for prescribing them birth control under the assumption that they were at risk of pregnancy. Validating and communicating that you understand the patient’s sexual and/or gender identity is important. You can emphasize that the hormones that are in these contraceptive methods can be used for pregnancy prevention but are often used for many different reasons. Frequently they are used for contraception but that we often use them in individuals who are abstinent from sex or who are having sex that would not put them at risk for pregnancy. These noncontraceptive indications for the use of hormones include acne, heavy or painful periods (as in Cory’s case), menstrual migraines, cyclic seizures, endometriosis, adenomyosis, or troublesome ovarian cyst formation (pain and/or bleeding from the cyst).
- While in Cory’s situation we may not be using a contraceptive method for pregnancy prevention, the nice thing about contraceptive drug development is that there are many different dosing regimens of these hormones that go beyond just pills. The patch, shot, ring, or IUD all should improve the bleeding and cramping from many etiologies while remaining discrete and not requiring daily dosing with a pill.
- Having a discussion regarding how these hormones may lighten or even take away periods as well as positively affect secondary sex characteristics (e.g., improve acne) may be a welcomed conversation to someone who is gender fluid or nonbinary.

**What other aspects of their health history would you want to discuss further with them?**

- As mentioned above, just because this patient currently has female partners doesn’t mean that they are not at risk for cervical dysplasia, STIs, and even unplanned pregnancy. Discussing the need for pap tests at regular intervals, as well as possible screening and/or prevention of STIs and pregnancy would be important.
- Suggestions for further discussion with the patient: smoking cessation, questioning further about alcohol use, sexual history, etc. If asking about past experiences, use open-ended questions so that the patient can fully explain themself.
- However, it may be that the lack of medical care/lack of trust in the medical system that set Cory up to avoid medical care in the past may be directly related to prior physicians getting too “preachy” or paternalistic about lifestyle choice.
- Therefore, sometimes it can be good to address the primary concern of the patient on the first visit and, maybe in a second visit or after a relationship of trust has been established, it would then be good to bring up these other health habits that may need further discussion. This may not mean that you need to ignore the smoking and alcohol use nearly entirely on the first visit, but you could alert them that you see the smoking and alcohol use as part of their health history, ask them if they have anything they would talk about today specifically to those two, and, if not, ask permission to address at another visit.

**Small-Group Case 3**

**You are an internal medicine provider about to see Cora, a 47-year-old cis-gender woman who has been your patient for several years. She is here for her annual visit with you. When talking to her, while she does not endorse any specific complaints or reason for her visit besides her annual check-in, she does not seem to be herself—she is less energetic, provides short answers, has trouble concentrating, and is less engaged overall. While you are thinking about medical conditions that may be contributing to her change in behavior, such as thyroid disorders, anemia, or depression, you are also considering social contributors, such as stresses surrounding Covid and her kids being home for virtual school, or new safety concerns in one of her relationships.**

**Upon doing a thorough review of symptoms and asking her about her social situation outside of your office and her feelings about being in the office with you today, you discover that her sleep has been disrupted significantly both because of worry about her dissolving sexual relationship with her husband and because of feeling hot with some regularity. You also learn that her most recent menstrual cycle length was 60 days and that for the last 18 months she has had her period every 3-6 months, lasting anywhere from 2 days to 9 days. She seems to have more episodes of feeling hot when she has longer intervals between her cycles.**

**What is the differential diagnosis for her regular sensation of feeling hot, particularly at night?**

- Vasomotor symptoms of perimenopause
- Hyperthyroidism
- Medications (e.g., Calcium channel blockers)
- Alcohol-related flushing
- Pheochromocytoma
- Carcinoid syndrome
- Emotional flushing: In case you need to know what this is: Skin flushing or blushing describes feelings of warmth and rapid reddening of your neck, upper chest, or face. Blotchiness or solid patches of redness are often visible when blushing. Flushing happens as a result of increased blood flow. Whenever there is more blood flow to an area of skin (such as your cheeks), the blood vessels enlarge to compensate. This enlargement is what gives skin the “flushed” effect. Flushed skin is a common physical response to anxiety, stress, embarrassment, anger, or another extreme emotional state. Facial flushing is often more of a social worry than a medical concern.
- Autonomic epilepsy: In case you need to know what this is: focal autonomic seizures with predominantly subjectively experienced phenomena are one type of epileptic 'aura'. An 'aura' is a subjective experience (which may be sensory, emotional, autonomic, or cognitive) felt by the individual having a seizure. The 'aura' reflects the initial seizure discharge in the brain. It may be an isolated phenomenon or progress to a focal motor seizure, to a focal impaired awareness seizure, or to a focal to bilateral tonic-clonic seizure. An aura is also known as a "warning".

**What other aspects of her history may help you prioritize one particular diagnosis on the differential?**

- Her age: In the U.S., 52 years is the average age of menopause (defined retrospectively at the final menstrual period followed by a year of amenorrhea). The Menopause Transition refers to the span of time when menstrual cycle and endocrine changes occur, beginning with variation in the length of the menstrual cycle and ending with the final menstrual period.
- Sexual symptoms: If the diagnosis is perimenopause, Cora has multiple potential contributors to these symptoms, including a) mood changes from both her lack of sleep, concentration, and from her potential relationship difficulties as a result of these sexual symptoms, b) Hormonal fluctuations with erratic ovulatory cycles that may affect sexual desire, and c) any genito-urinary symptoms from decreased estrogen, such as vaginal dryness, that may be affecting sexual pleasure.
- Sleep disturbances: Perimenopause contributors of this include: vasomotor symptoms (which can be very uncomfortable), depressed mood, and other issues that may be causing worry as mentioned in mood disturbances directly below.
- Mood disturbances: As mentioned above, erratic fluctuations in hormones as the ovaries spit out the last few eggs can significantly affect mood. Relationships may also be affected as well, as mentioned under sexual symptoms above. Some women may have mixed emotions or even frank depression about moving into menopause, aging, and/or losing their fertility.

**What further work-up would you want to do to confirm your suspicion and rule out other diagnoses?**

- The Abnormal Uterine Bleeding needs to be evaluated in order to rule out a) pregnancy, b) ovarian dysfunction (i.e. ,anovulation, PCOS), c) structural growths (i.e., Leiomyoma, polyps), d) endometrial hyperplasia/cancer, e) Thyroid dysfunction
  - Work-up includes: Physical exam, pregnancy test, cervical cytology, endometrial biopsy, pelvic u/s, TSH, prolactin, testosterone, DHEAS
- There is no role for routinely checking FSH and/or estradiol levels in patients who are over age 40 years and have a symptom constellation suggestive of menopause. After ruling out other diagnoses through the work-up above, the diagnosis of menopause is usually empiric.
  - Patients under the age of 40 years with the symptoms suggestive menopause should have an FSH checked to make the diagnosis of premature ovarian failure. Similarly, if patients are undergoing an infertility work-up, FSH may be drawn either before or after the age of 40 years in order to give an indication of ovarian reserve.

**Once you make your diagnosis, how might you want to treat her underlying diagnosis?**

- Nonhormonal, nonprescription options: Depending on symptoms, this is often tried first as a way to make symptoms manageable, especially because many women have vasomotor symptoms for 10+ years. These may be prioritized in Cora, specifically as should improve sleep and mood as well as hot flashes.
  - Moderate alcohol use or less, avoid smoking (current and post smokers have higher odds of number and severity of hot flashes)
  - Maintain a healthy body weight: higher BMIs (>27) are a predictor of hot flash frequency
  - Regular exercise
  - Dress in layers, maintain low ambient temp (though limited supporting data)
  - Paced respiration, mindfulness-based stress reduction, clinical hypnosis, cognitive behavioral therapy
- Oral contraceptives: Can use in healthy, nonsmoking, normotensive perimenopausal patients up until the age of 55 years. This would be a good option for Cora since she is only 47 years old.
  - Additional benefits include contraception (pregnancy is possible in the perimenopausal time) and management of irregular bleeding
- Nonhormonal prescription medications
  - SSRIs (Paxil 75 mg daily only FDA approved nonhormonal regimen for vasomotor symptoms, but other SSRIs and SNRIs such as venlafaxine and desvenlafaxine have shown benefit)
  - Gabapentin up to 900 mg/d in divided doses, although not FDA approved, has shown benefit, especially for nighttime vasomotor symptoms
- Hormonal prescription medication
  - Estrogen/Progestin vs. Estrogen alone
    - Estrogen alone shown to decrease vasomotor symptoms by up to 70%
    - Progestin needed for endometrial protection if the uterus is present
    - Some benefit with micronized progesterone alone in decreasing vasomotor symptoms (for patients in whom estrogen is contraindicated)
    - Options: oral, transdermal, vaginal
  - Once HRT is started in patients:
    - There should be ongoing discussions about continued need for HRT
    - Treatment should be at the lowest possible dose for the shortest possible time
    - WHI data showed increased breast CA risk in patients taking CEE and Medroxyprogesterone but newer data suggests that micronized progesterone carries less breast CA risk
    - You should do continued assessment for development of new risk factors (new heart disease, Cancers, VTE, etc.)

**What aspects of a patient’s past medical history may limit your therapies?**

- Hormone replacement therapy is contraindicated in patients with a history of breast or endometrial cancer, risk for VTE, some liver disease, and undiagnosed vaginal bleeding
- Age: Coronary heart disease risk is increased with hormone therapy if they initiate hormone therapy after the age of 60 years or > 10 years after menopause onset

**For Cora specifically, what therapies might we prioritize given her specific symptoms?**

- For Cora, since she is having multiple symptoms that might be interrelated, she would likely benefit from the lifestyle modification, relaxation, and CBT nonhormonal, non-Rx options described above, as these techniques would improve hot flashes, mood, sleep—all the things that are interconnected—simultaneously
- Also, Cora may benefit from use of a hormonal contraceptive as a way to manage both the symptoms related to hormonal fluctuations, and also her irregular periods and bleeding profile.
- We also want to dive in deeper in our history to find out about her sexual relationship discord to find out what all may be contributing to this: decreased desire (hormones), decreased interest (mood), and/or genito-urinary symptoms. If the latter, consider if she should be started on a vaginal lubricant to help any genito-urinary symptoms, at least until the hormonal contraceptives start to provide greater support for that tissue (this tends to be a symptom of more prolonged low hormonal environment, so less common in perimenopause, but certainly is possible and happens for many patients.
- Also check for intimate partner violence: a patient may not name it as such, especially emotional abuse, sexual coercion, cultural, religious belief about patient role or sexual duties—patient response to these impacts can vary person by person. Use TIC to address any shame, embarrassment, assumptions you might make as a clinician; also the meaning to women of losing menses is important, can bring up emotions that need addressing including past trauma, shame, loss, grief, or relief—need a trusting relationship to discuss this, as it has implications for adherence to treatment and treatment choices as well—DO NOT just tell a patient to go to therapy, it’s in her mind, or it’s just a mind-body issue—women face stigma in medical system (pain ignored, disbelieved, etc.). This is an important milestone for a woman and should be treated accordingly.

**When a patient, such as Cora, has a multitude of symptoms that are interconnected (hot flashes contributing to sleep disturbances; sleep disturbances and sexual symptoms contributing to depressed mood), how do you consider and prioritize treatment in a patient-centered way?**

- As mentioned above, several therapies may help several of Cora’s symptoms, which makes management a little tricky. Apart from the management recommendations listed above, a patient-centered approach should be applied to the treatment of her depressed mood specifically. Conveniently, SSRIs such as Paxil can treat both depressed mood and hot flashes. Depending on how many other medications or therapies that are given to the patient, a discussion regarding the use of an SSRI to treat her mood symptoms should be had. Follow-up with this patient should be relatively soon, such as < 3 months, so it may be okay to delay and see if the other recommendations and therapies result in improved mood. But, it may be reasonable to start an SSRI at this visit as well, with again, quick follow-up given all the therapies that have been started. If a patient chooses a hormonal therapy, such as a levonorgestrel-containing IUD, to control the bleeding but that may minimally affect the systemic symptoms of hormonal fluctuations, use of an SSRI to treat mood and vasomotor symptoms may be more warranted.
