# Supplementary material for: Benefits of Hormonal Contraception Across the Lifespan: A Case-Based, Interactive Curriculum
Source: MedEdPORTAL. 2025 Apr 4;21:11512. doi: 10.15766/mep_2374-8265.11512 (PMC11968450; doi:10.15766/mep_2374-8265.11512)
Supplement: Supplementary file 1 — Student Guide and Case 1.docxCase 2.docxCase 3.docxCDC Eligibility Criteria for Contraceptive Use.pdfBN How Well Does Birth Control Work.pdfRHAP Birth Control Across the Gender Spectrum.pdfCounseling for the Hormones Found in Contraceptives.pptxCase-Based Collaborative Learning.pptxFaculty Guide.docxLongitudinal Assessment Questions.docx [file mep_2374-8265.11512-s001.zip › A. Student Guide and Case 1.docx]

**Student Guide**

Appendix A: This document serves as the student guide to the curriculum overall and to the first case-based collaborative learning case dedicated to pregnancy prevention in a medically complex patient. Learners should have access to this guide in advance of the session to be able to prepare. We give this to our preclinical second-year students 2 weeks before the session.

**Reproductive Potential and Gynecologic Needs: A Patient-Centered Approach**

**Overview**: Patients’ reproductive and gynecologic needs affect their lives, their health, and their interface with the health care system. The specific aim of this course will be to examine common patient encounters and health care needs that are currently often situated within gynecology alone but must instead be considered within multiple health care disciplines. Within this learning we will emphasize a patient-centered approach, with an emphasis on individual patient social contexts that may affect the health care encounter and health care decision-making.

**By the end of this activity, learners will be able to:**

1. Identify the need for and the considerations involved in providing family planning as part of the medical care plan for pregnancy-capable individuals at time of a new diagnosis of a chronic medical condition.
2. Using a trauma-informed care approach, describe assumptions, language, and gynecology health care practices which may contribute to medical mistrust.
3. Describe practice improvements to mitigate medical mistrust for a diversity of patients across the gender identity spectrum.
4. Describe the approach to history taking for perimenopausal patients.
5. Describe the role of exogenous hormones in perimenopausal symptom management.

**Agenda:**

1:00-1:40 Contraceptive Counseling: A Patient-Centered Approach

1:40-2:00 BREAK to get to Learning Labs

2:00 to 4:00 Case-based Collaborative Learning within Learning Studios

2:00-2:35 Case 1: Reproductive potential in the setting of chronic disease

2:40-3:15 Case 2: Building trust and a patient-centered practice in gynecology

3:20-3:55 Case 3: Care in patients at the end of the reproductive life course

**Preparatory Work:**

(1) Read the three small group cases and their associated questions (Appendix A–C)

Additional Resources for the Principal Clerkship Experience:

(1) CDC. 2024 Summary Chart of US Medical Eligibility Criteria for Contraceptive Use.^1^ Please recognize this resource is updated on occasion and you should look for an updated version every 2 years when using this resource in the future. (Appendix D)

(2) Bedsider Birth Control Network. Birth Control Methods Comparative Chart.^2^ (Appendix E)

(3) Reproductive Health Access Project. Birth Control Across the Gender Spectrum.^3^ (Appendix F)

^1^Image by CDC, retrieved on November 11, 2023 from: https://www.cdc.gov/contraception/media/pdfs/2024/07/us-mec-summary-chart-color-508.pdf?mc_cid=d8d9bc1493&mc_eid=69a2a30a0f. Image is in the public domain. The images summarizes data from: Nguyen AT, Curtis KM, Tepper NK, et al. U.S. Medical Eligibility Criteria for Contraceptive Use, 2024. MMWR Recomm Rep 2024;73(No. RR-4):1–126. DOI: <http://dx.doi.org/10.15585/mmwr.rr7304a1>

^2^Image by UCSF School of Medicine Bixby Center, retrieved on November 11, 2023 from: https://www.bedsider.org/. Creative Commons attribution NonCommercial - NoDeriv 3.0 Unported License.

^3^Image by Reproductive Health Access Project, retrieved on November 11, 2023 from: https://www.reproductiveaccess.org/resource/birth-control-across-the-gender-spectrum/. Creative Commons License associated: https://creativecommons.org/licenses/by-nc-sa/4.0/.

**Small-Group Case 1**

**Suyin is a 28-year-old cis-gender female who comes to the clinic with her husband. As she explains her symptoms, she appears nervous. She describes how a few weeks ago, she started to experience joint stiffness in her fingers, wrists, and knees. She experiences pain upon bending the affected joints on both sides of the body. The stiffness and pain are worst in the morning and take about 2 to 3 hours to dispel. She is concerned because she is feeling very fatigued, and it is difficult for her to go about her daily activities.**

**Her history, a physical exam, and abnormal findings in bloodwork are consistent with a diagnosis of rheumatoid arthritis (RA).** **What do you think about this diagnosis in this patient? Does it seem plausible? Are there any special considerations to be made for this particular patient with this particular diagnosis?**

**You consider prescribing methotrexate, a common first-line treatment for RA. Methotrexate begins to relieve symptoms relatively quickly and has a lower cost than most other options. Adverse drug events are relatively rare, although patients should limit alcohol intake. When considering whether to prescribe the drug, what information would further help you decide?**

**You suggest starting on methotrexate to Suyin, and she asks about side effects. You explain that GI symptoms are the most frequent adverse effects, that the drug could affect liver function at high doses, and that it is teratogenic. When you explain teratogenicity, the patient looks concerned. How would you continue the conversation?**

**You ask Suyin if she thinks she might like to become pregnant in the next year, and she responds that she is not sure. She is not currently trying to become pregnant.**

**Given this information, would you still prescribe a teratogenic medication? How would you counsel her?**

**Because she is not comfortable using hormonal birth control, Suyin states that she always uses condoms. Would your counseling change if she decided she did not want to become pregnant in the next year but only would like to use barrier methods of contraception?**
